# Supplementary material for: Activity-Dependent Exocytosis of Lysosomes Regulates the Structural Plasticity of Dendritic Spines
Source: Neuron. 2017 Jan 4;93(1):132–46. doi: 10.1016/j.neuron.2016.11.013 (PMC5222721; doi:10.1016/j.neuron.2016.11.013)
Supplement: Document S1. Supplemental Experimental Procedures and Figures S1–S9 [file mmc1.pdf]

Neuron, Volume *93*

Supplemental Information

Activity-Dependent Exocytosis of Lysosomes Regulates the Structural Plasticity of Dendritic Spines

Zahid Padamsey, Lindsay McGuinness, Scott J. Bardo, Marcia Reinhart, Rudi Tong, Anne Hedegaard, Michael L. Hart, and Nigel J. Emptage

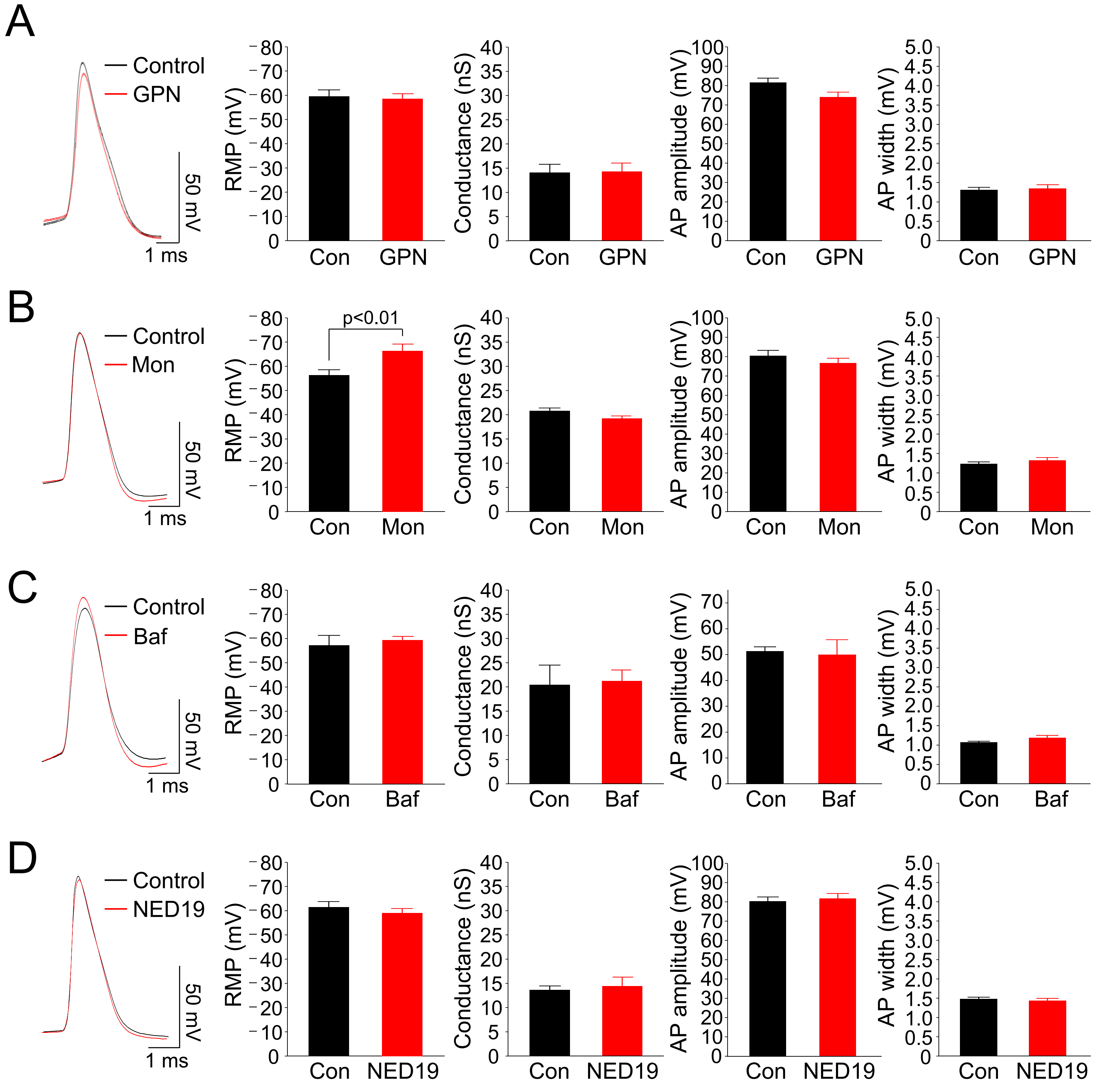

Supplementary Figure 1

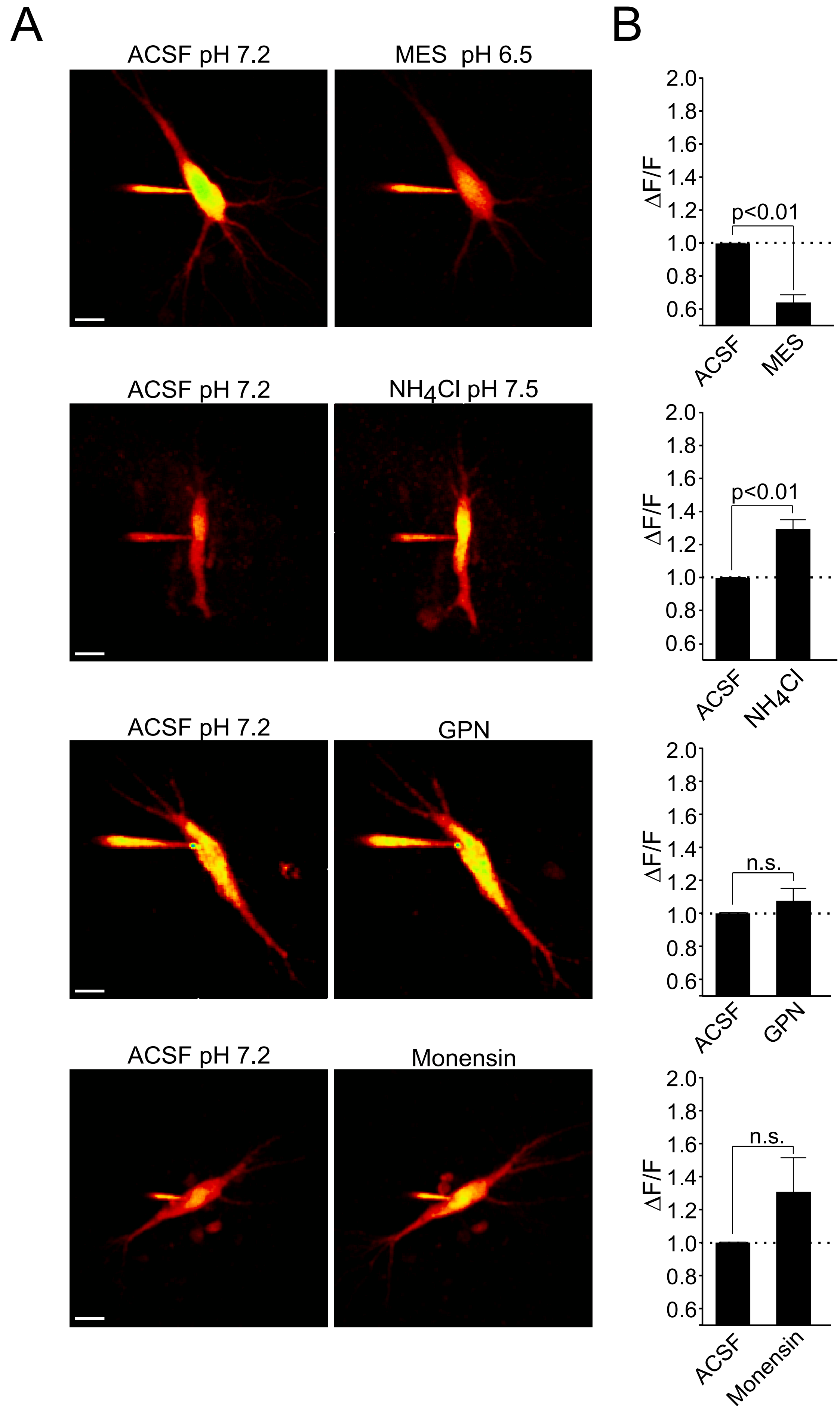

Supplementary Figure 2

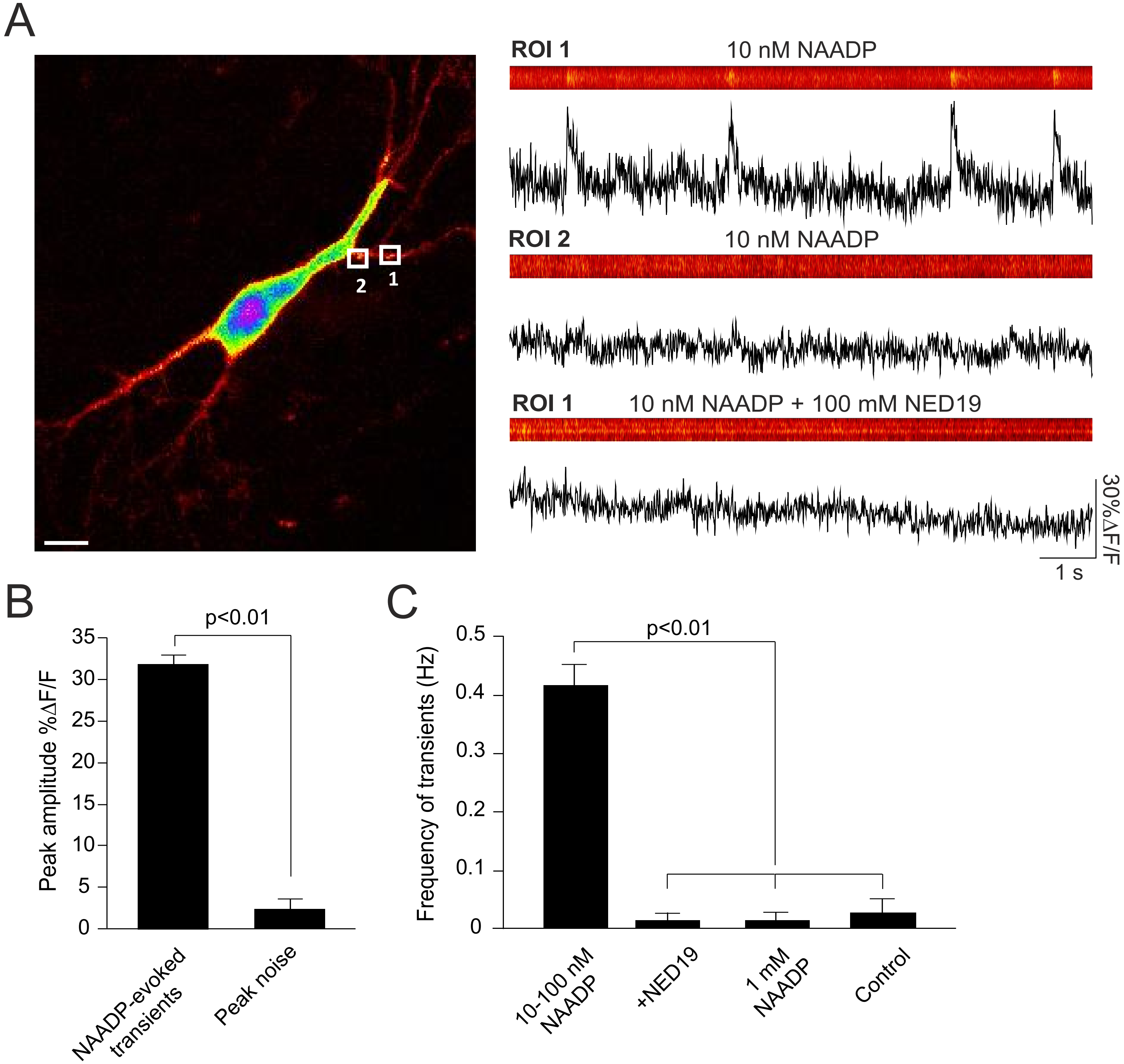

Supplementary Figure 3

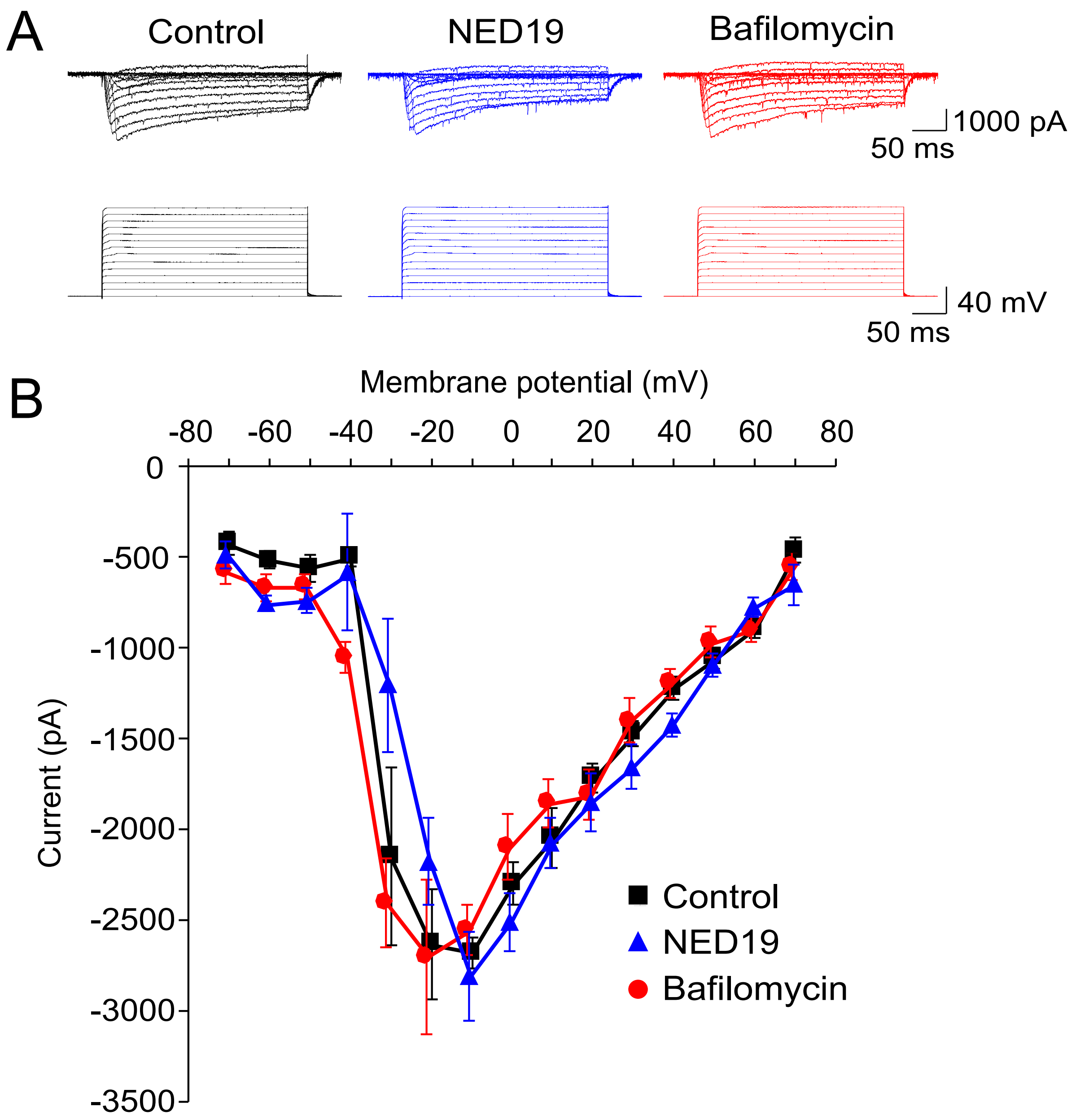

Supplementary Figure 4

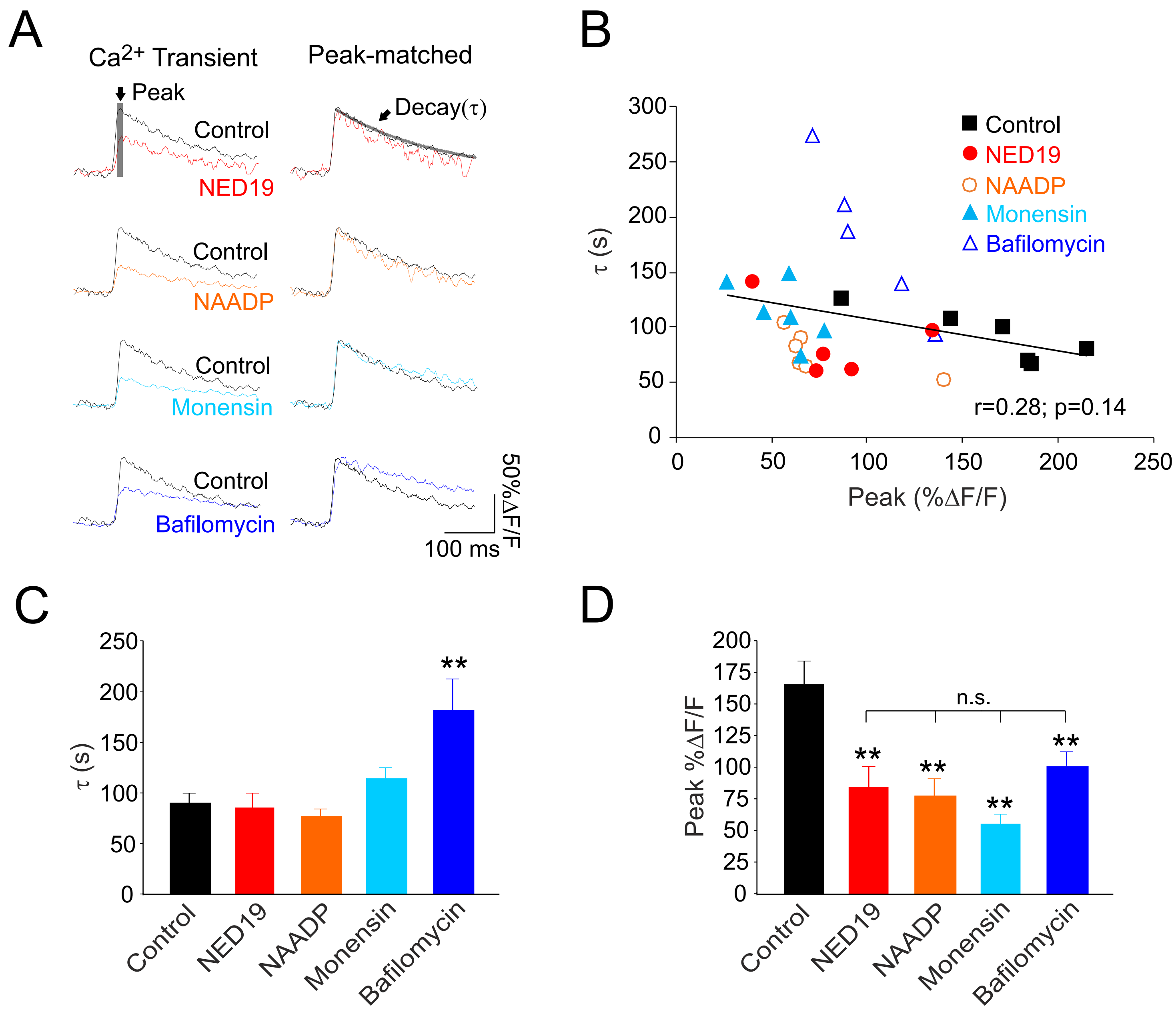

Supplementary Figure 5

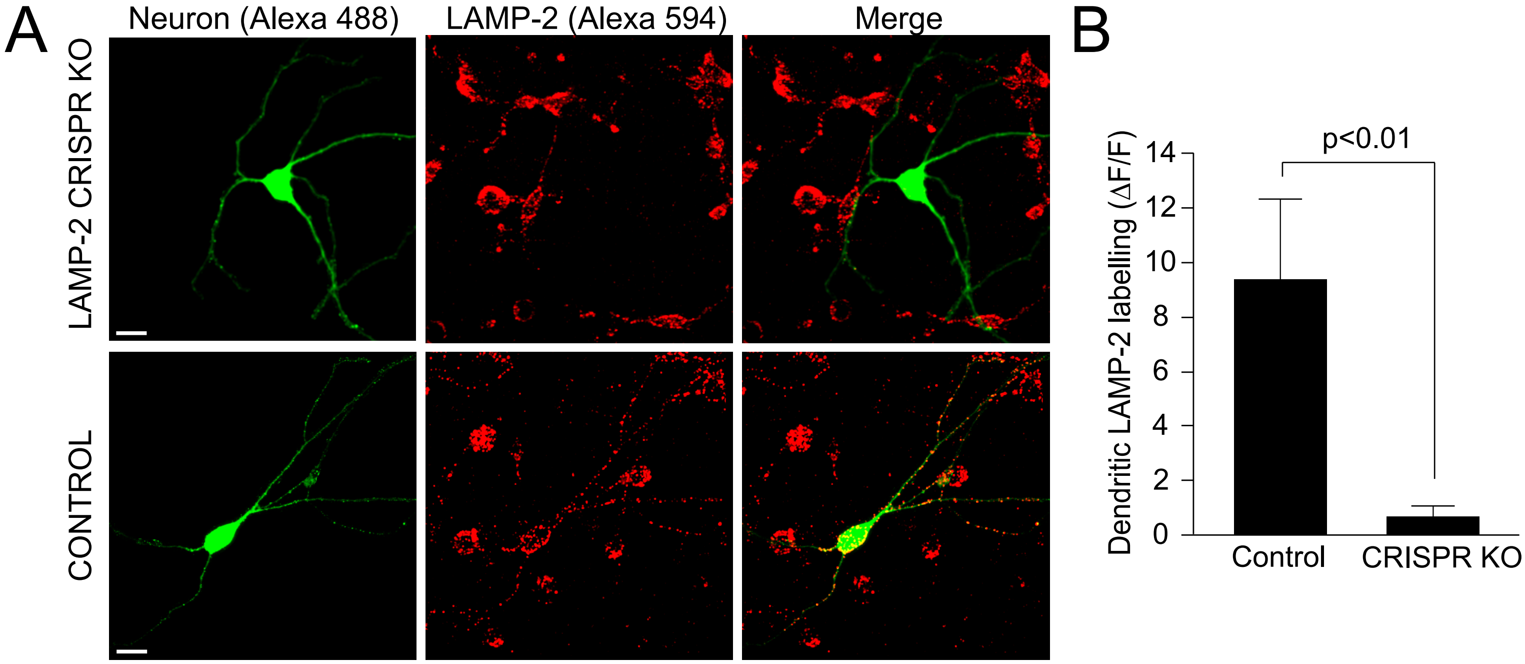

Supplementary Figure 6

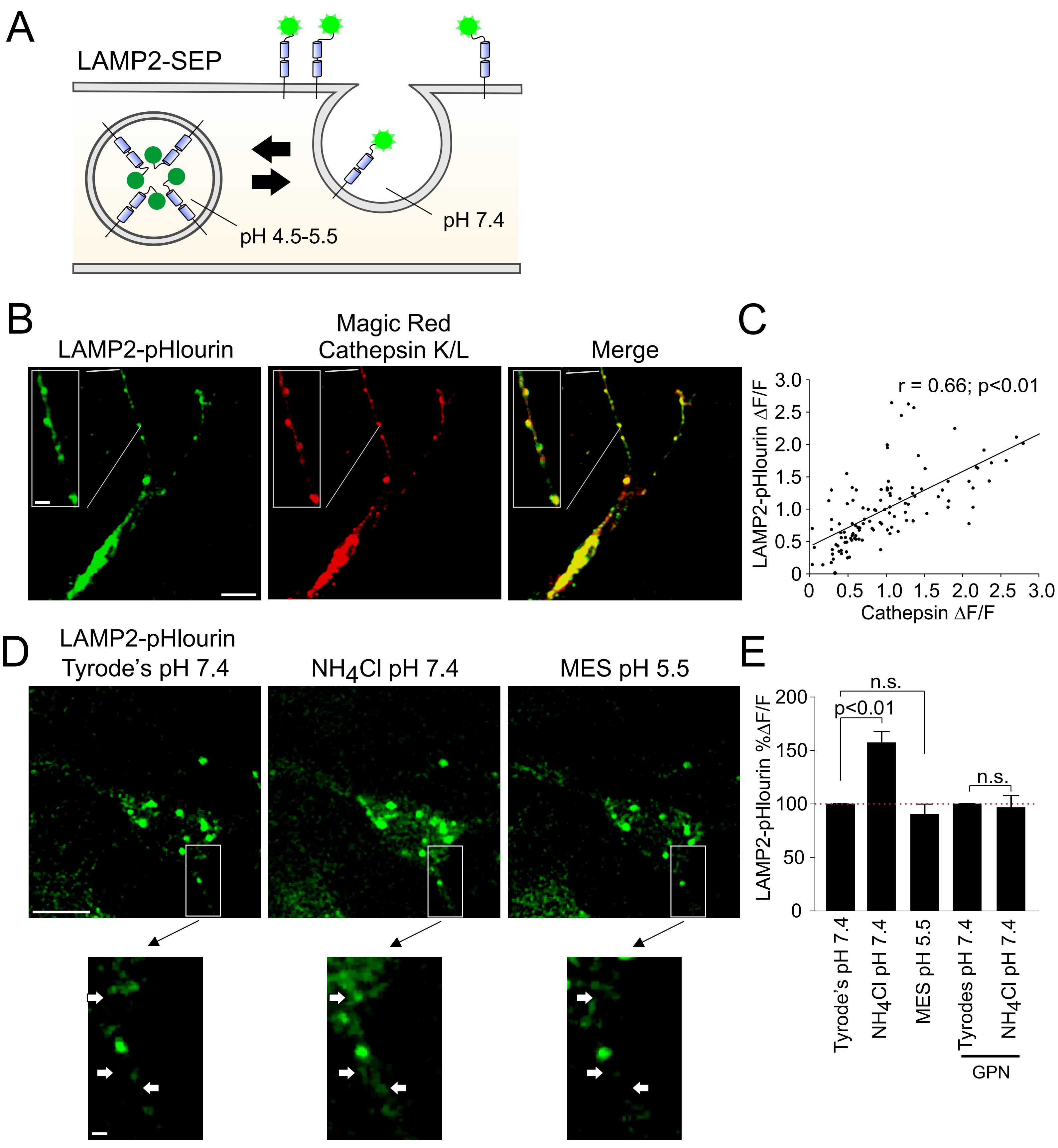

Supplementary Figure 7

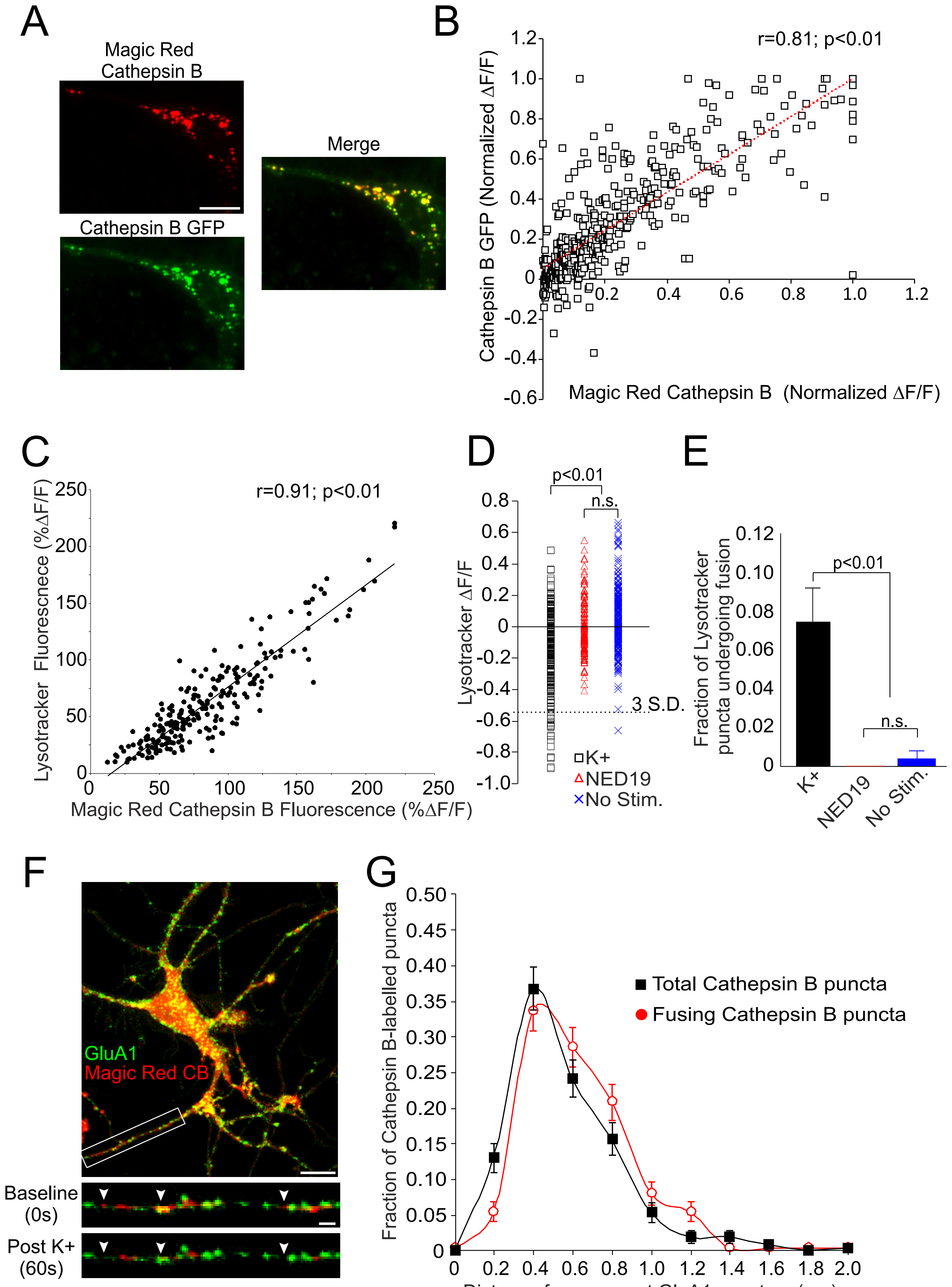

Supplementary Figure 8

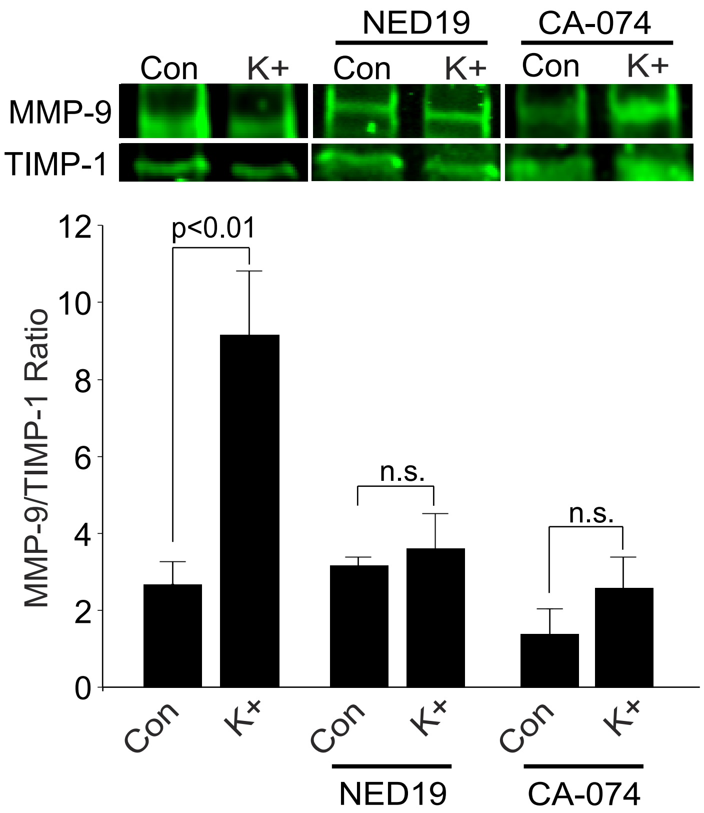

Supplementary Figure 9

Figure S1. Electrophysiological characterization of hippocampal neurons and action potential waveform in the presence of pharmacological inhibitors of lysosomal function. Related to Figure 1 and 2. Electrophysiological analysis of **(A)** GPN, **(B)** monensin (mon), **(C)** bafilomycin (baf), and **(D)** NED-19 are shown. For each drug, a sample action potential waveform is shown, along with group averages of cell resting membrane potential (RMP), membrane conductance, action potential (AP) amplitude, and AP width, calculated at AP half-height. None of these parameters are altered by the drug, with the exception of monensin, which results in significant hyperpolarization of the resting membrane potential. All pair-wise comparisons are non-significant, unless otherwise noted (n=5-7 cells/condition). Error bars represent S.E.M. Significance was assessed with Mann-Whitney tests.

Figure S2. Characterization of intracellular pH in the presence of pharmacological inhibitors of lysosomal function. Related to Figure 1 and 2. Drug-induced changes in intracellular pH were assessed using fluorescein, the fluorescence of which is pH sensitive. **(A)** Sample images of neurons patch-loaded with fluorescein (scale bars=10 µm). Images were taken in ACSF (pH=7.2) and following bath application of various solutions. Bath application of an acidic MES-buffered ACSF solution (pH 6.5) resulted in a significant reduction in fluorescence, whereas bath application of an alkaline NH_4Cl-buffered ACSF solution (pH 7.5) resulted in a significant increase in fluorescence. Fluorescein fluorescence was therefore bidirectionally sensitive to changes in pH. Bath application of GPN, which pharmacologically permeablizes the lysosome, had no substantial effect on fluorescein fluorescence. Bath application of the Na^+/H^+ ionophore monensin, however, had a tendancy an increase in fluorescence, consistent with an increase in intracellular pH that has previously been reported with the drug (Erecinska et al., 1991). This increase, however, was not significant. **(B)** Group averages are shown (n=5-6 cells/condition). Error bars represent S.E.M. Non-significant group comparisons are denoted by n.s. Significance was assessed with Mann-Whitney tests.

Figure S3. Imaging of lysosomal Ca^{2+} release in response to NAADP. Related to Figure 2 and 3. **(A)** Right. Representative image of a hippocampal pyramidal neuron (scale bar=10 µm) loaded with Oregon Green BAPTA-1 (OGB-1) and with NAADP (10-100 nM). Two regions of interest (ROI 1 and ROI 2) were examined (white boxes) along the apical dendrite by restricting laser scanning to a line along both ROIs. Left. The resulting line scans, along with their quantified fluorescence (%ΔF/F), are shown. In ROI 1, small and rapid Ca^{2+} transients were observed. These transients were often rhythmic, highly localized, and did not spread to ROI 2. The addition of the NAADP-antagonist NED-19 abolished these transients. **(B)** Average amplitude of NAADP-evoked Ca^{2+} transients (n=36 events recorded across 3 cells in 6 regions of interest), compared with peak fluorescence amplitude of noise. **(C)** Average frequency of NAADP-evoked Ca^{2+} transients (n= 6 regions of interest recorded across 3 cells/condition). In the presence of high concentrations of NAADP (1 mM), NED-19, or in the absence of NAADP (control), Ca^{2+} transients were rarely observed (<2 across experiments), and when detected, were isolated events showing no rhythmicity.

Figure S4. Voltage-gated Ca^{2+} channel currents are unaffected by inhibition of lysosomal Ca^{2+} signalling. Related to Figure 2. **(A)** Sample of voltage-gated Ca^{2+} channel (VGCC) currents recorded from CA1 neurons during 10 mV step depolarizations between -70 and +70 mV in control, NED-19 treated, and bafilomycin treated slices. **(B)** Group average current-voltage plots for peak VGCC currents (n=7-8 cells/conditions). Significance was assessed using a two-way ANOVA. VGCC currents recorded in control, NED-19, and bafilomycin treated cells were not significantly different from one another.

Figure S5. Characterization of Ca^{2+} handling in the presence of pharmacological inhibitors of lysosomal signalling. Related to Figure 2. **(A)** Ca^{2+} handling was quantified by examining the decay of back-propagating action potential (bpAP)-evoked Ca^{2+} transients recorded in CA1 dendrites. Left. Sample Ca^{2+} transients recorded across experimental conditions. Shaded box and arrow denote the window in which the peak value of the transient was calculated. The control trace (black) has been

duplicated in each example. Right. Ca^{2+} transients have been peak-matched to controls in order to calculate the time constant of decay (τ). **(B)** The peak amplitude and τ are plotted for each experiment across experimental conditions. There is no significant correlation between the two variables suggesting that measurements of drug-induced effects on peak Ca^{2+} influx are not likely to be effected by potential drug-induced effects on Ca^{2+} handling. Significance was assessed using the Pearson correlation coefficient. **(C)** Average τ is plotted across experimental conditions (n=5-7/condition). Bafilomycin led to a significant increase in τ, suggesting that it impaired the ability of the cell to extrude Ca^{2+} following the bpAP. **(D)** Average peak Ca^{2+} amplitude is plotted across experimental conditions (n=5-7/condition). All pharmacological inhibitors of lysosomal Ca^{2+} signalling reduced peak Ca^{2+} influx relative to control, independent of their effects on Ca^{2+} handling (C). Error bars represent S.E.M. Asterisks (**) denote significance at p<0.01 from control levels, unless otherwise noted. Significance was assessed using ANOVA and post-hoc Bonferroni tests.

Figure S6. Confirmation of anti-LAMP2 antibody specificity. Related to Figure 4. **(A)** (Green) Sample image of a neuron targeted for CRISPR/Cas9-mediated knock-out of LAMP-2 and a control neuron. (Red) Cultures were live-cell immunolabelled with anti-LAMP2 antibodies. Neurons expressing the CRIPSR/Cas9 plasmid showed negligible levels of surface labelling, confirming the specificity of the antibody in our experimental setup. **(B)** Quantification of LAMP-2 immunolabelling in control and CRISPR/Cas9-transfected neurons. Error bars reflect S.E.M. Significance was assessed using a Mann-Whitney test.

Figure S7. Characterization of LAMP2-SEP. Related to Figure 5. **(A)** Schematic of LAMP2-SEP. Superecliptic pHlourin (SEP) was tagged to the lumenal domain of lysosome-associated protein LAMP-2. At rest, LAMP2-SEP is quenched by the acidic intralumenal pH of the lysosome (pH=4.5-5.5), but fluoresces following lysosomal fusion with the plasma membrane, when it is exposed to the pH-neutral environment of the extracellular fluid. During endocytosis, the retrieval and re-acidification of the lysosome quenches LAMP2-SEP fluorescence. **(B)** *In vitro* characterization of LAMP2-SEP localization. Representative image of dissociated hippocampal neuron (scale bar=20 µm) transfected with LAMP2-SEP (green channel) and loaded with red Cathepsin K and L fluorogenic substrates (red channel) to identify lysosomal compartments; the use of LysoTracker red is not recommended for lysosomal labelling in co-localization studies (Freundt et al., 2007). Magnified images (scale bar=4 µm) of the dendrite (white) are shown. LAMP2-SEP co-localized with lysosomes (yellow channel). **(C)** Analysis of LAMP-2-colocalization with lysosomal compartments shown in (B) across cells (n=5 cells). There is a significant correlation (r=0.66; n=115 puncta; p<0.01) between Cathepsin-associated fluorescence and LAMP-2-pHlouin associated fluorescence. **(D)** *In vitro* assessment of LAMP2-SEP pH-sensitivity. Representative images of a dissociated hippocampal neuron transfected with LAMP2-SEP (scale bar=20 µm) along with magnified images (scale bar=2 µm) of a dendrite (white box). Application of NH_4Cl (pH 7.4) to neutralize acidic compartments increased LAMP2-SEP fluorescence and resulted in the emergence of new fluorescent puncta in the dendrites (white arrows), whereas application of acidic MES (pH 5.5) returned fluorescence to baseline levels, previously recorded in neutral Tyrode's solution (pH 7.4). These data confirm that LAMP2-SEP is pH-sensitive and trafficked to acidic organelles. **(E)** Group averages (n=5 cells) of data shown in (D). Cultures were also pre-treated with GPN, which specifically permeabilizes lysosomal stores, resulting in the dissipation of their proton gradient. Under these conditions, application of NH_4Cl (pH 7.4) had no effect on LAMP2-SEP fluorescence, confirming that increases in LAMP2-SEP fluorescence required de-acidification specifically of lysosomal compartments. Non-significant group comparisons are denoted by n.s.

Figure S8. Magic Red Cathepsin B labels Cathepsin B-containing puncta, which co-localize with LysoTracker staining and undergo fusion in the vicinity of synapses. Related to Figure 6. **(A)** Sample image of a neuron loaded with Magic Red Cathepsin B (red) and expressing Cathepsin B-GFP (scale bar=10 µm). Magic Red fluorescence localized well with GFP-labelled puncta. **(B)** Plot of Magic Red fluorescence against GFP fluorescence for labelled puncta (440 puncta imaged across 8

cells). There is a strong and significant correlation between Magic Red fluorescence and Cathepsin B-GFP fluorescence, suggesting that the intensity of Magic Red fluorescence accurately reflects the levels of Cathepsin B for a given puncta. Significance was assessed using the Pearson correlation co-efficient. **(C)** Plot of LysoTracker Green fluorescence against Magic Red fluorescence for labelled puncta (254 puncta from 5 cells) from experiments shown in Figure 6A. There is a strong and significant correlation between Magic Red fluorescence and LysoTracker Green fluorescence. Significance was assessed using the Pearson correlation co-efficient. **(D)** Activity-dependent LysoTracker de-staining is similar to that of Magic Red Cathepsin B de-staining (shown in Figure 6B,C). Change in LysoTracker fluorescence was examined after 1) K^+ treatment of cultures 2) K^+ treatment in cultures pre-treated with NED-19 and 3) unstimulated cultures (no stim.) (n=152-254 puncta from 5-6 cells/condition). Dashed line marks 3 standard deviations from average fluorescent changes recorded in unstimulated controls (no stim.). K+ stimulated cultures showed greater levels of destaining than NED-19-treated cultures or unstimulated cultures. Significance was assessed with Kruskal-Wallis and post-hoc Dunn's tests. **E)** Fraction of LysoTracker-labelled puncta undergoing fusion, as defined by a loss of fluorescence greater or equal to 3 standard deviations from the average fluorescent changes recorded in unstimulated controls (no stim.). K+ stimulated cultures showed greater fraction of fusing vesicles than NED-19-treated cultures or unstimulated cultures. Significance was assessed with z-tests (Boneferroni correction). **(F)** Confocal image of a dissociated neuron (scale bar=5 µm) labelled with Magic Red Cathepsin B substrate (red) and immunostained for GluA1 (green) to label synapses. The dendrite in the white box is magnified below (scale bar=2 µm). K^+ treatment resulted in loss of Cathepsin B-associated fluorescence from several puncta in the vicinity of GluA1 labelled puncta (white arrow heads). **(G)** Distribution of Cathepsin B puncta from GluA1 labelled puncta. The majority of Cathepsin B puncta (approx. 95%), including those undergoing activity-dependent fusion, were found within 1 µm of a GluA1 puncta.

Figure S9. Neuronal depolarization increases MMP-9/TIMP-1 ratios in a manner dependent on lysosomal Cathepsin B. Related to Figure 6. Top. Sample western blots of MMP-9 and TIMP-1 expression levels in cultured hippocampal slices. Bottom. Quantification of the MMP-9/TIMP-1 ratio (n=5-7 slices/condition). Inhibition of lysosomal Ca^{2+} signalling and fusion with NED-19 or inhibition of extracellular Cathepsin B activity with CA-074 prevented activity-induced increases in the MMP-9/TIMP-1 ratio. Error bars represent S.E.M. Significance was assessed with ANOVA and post-hoc Bonferroni tests.

Movie S1. Imaging activity-dependent mobilization of lysosomes at the cell surface. Related to Figure 4. Sample video clip of TIRF imaging experiments of lysosomes shown in Figure 4A. Two dissociated hippocampal neurons (soma are on the bottom right) were loaded with LysoTracker (green fluorescence) to label lysosomes. The experiment starts with a baseline recording. After which, the culture is stimulated with 45 mM K^+; this period is denoted with a label reading "K^+" on the top left corner of the video. During stimulation, there is a rapid emergence of fluorescence puncta throughout the dendritic arbour, which are not seen during baseline imaging. Frames were acquired at the rate of 1/s and are played at a rate of 4/s.

Movie S2. Imaging activity-dependent lysosomal fusion using LAMP2-SEP. Related to Figure 5. Sample video clip from experiments shown in Figure 5B. A dissociated hippocampal neuron (soma on the top left) was transfected with LAMP2-SEP (green fluorescence). The experiment starts with a period of photobleaching (denoted by the label "photobleach" on the top right corner of the clip), in order to eliminate baseline fluorescence of LAMP-2 that is already present on the cell surface. Following photobleaching, there is a baseline recording, after which the culture is stimulated with 45 mM K^+ (denoted by the label "K^+" on the top right corner of the clip). During stimulation, there is a rapid emergence of fluorescence puncta throughout the dendritic arbour, which are not seen during baseline imaging. Frames were acquired at the rate of 1/s and are played at a rate of 4/s.

Supplemental Experimental Methods

Acute hippocampal slices

Acute hippocampal slices (350-400 µm) were prepared from 2-3 week old male Wistar rats (Harlan). A sucrose-based ACSF solution (in mM: 85 NaCl, 65 sucrose, 26 $NaHCO_3$, 10 glucose, 7 $MgCl_2$, 2.5 KCl, 1.2 NaH_2PO_4, and 0.5 $CaCl_2$; pH=7.2-7.4) was used for the dissection. The whole brain was sliced along a coronal plane using a Microm HM 650V vibratome (Thermo Scientific), and dorsal hippocampi was dissected out from each slice. Hippocampal slices were recovered for at least 1 hour prior to use at room temperature in ACSF (in mM: 120 NaCl, 2.5 KCl, 2 $CaCl_2$, 1 $MgCl_2$, 1.2 NaH_2PO_4, 26 $NaHCO_3$, and 11 glucose; pH=7.2-7.4), bubbled with 95% O_2 and 5% CO_2. Slices were then transferred to the recording chamber and perfused (3 mL/min) with heated (32-34°C) and carbogenated (95% O_2 and 5% CO_2) ACSF, containing ascorbic acid (0.2 mM) and Trolox (1 mM) to minimize photodynamic damage during imaging.

Cultured hippocampal slices

Transverse 350µm hippocampal slice cultures were prepared from male Wistar rat pups, postnatal day 7 (Harlan UK) as previously described (Emptage et al., 1999). Slices were maintained 7-14 days prior to use at 37°C and 5% CO_2. Culture medium was replaced every 2-3 days and was composed of 50% Minimum Essential Media, 25% heat-inactivated horse serum, 23% Earl Balanced Salt Solution, and 2% B-27 (Invitrogen) with added glucose (6.5 g/L). During experimentation, slices were perfused (1-2mL/min) with heated (32-34°C) and carbogenated (95% O_2 and 5% CO_2) ACSF composed of (in mM): 145 NaCl, 16 $NaHCO_3$, 11 glucose, 2.5 KCl, 2-3 $CaCl_2$, 2 $MgCl_2$, 1.2 NaH_2PO_4 (pH=7.2-7.4). Additionally, 0.2 mM ascorbic acid and 1 mM Trolox were added to minimize photodynamic damage during imaging

Dissociated hippocampal cultures

Dissociated rat hippocampal neurons were prepared from E18 or P1 male Wistar pups. Hippocampi were dissected in cold Hank's Balanced Salt Solution (ThermoFisher) containing no Mg^{2+} or Ca^{2+}. Meninges were removed and tissue was incubated with trypsin (0.05%) at 37°C for 10-15 minutes. Hippocampi was then rinsed and re-suspended in serum-containing culture medium, comprising of NeuroBasal-A supplemented with 2% heat-denatured fetal calf serum, 2% B27 supplement, 1% GlutaMAX (200 mM stock), and 1% penicillin/streptomycin (10 000U/mL stock) (Thermo Fisher). The tissue was then gently triturated 30 times using a standard P1000 pipette tip, and viable cells counted in Trypan Blue (0.2%) using a hemocytometer. Cells were plated onto 18mm coverslips, pre-coated with poly-D-lysine (0.1 mg/mL) and fibronectin (0.05 mg/mL), at a density of 30 000-60 000 cells/coverslip. Hippocampal neurons were left to adhere to the coverslips for approximately 2 hours, before being placed in 12-well plates with 2mL/well of fresh serum-containing medium. Cultures were fed twice a week by replacing half of the medium with serum-free medium comprising of NeuroBasal-A supplemented with 2% B27 supplement and 1% GlutaMAX (200 mM stock). Cultures were used at DIV 10-21 and imaged at room temperature in Tyrode's buffer (in mM: 128 NaCl, 5 KCl, 1 $MgCl_2$, 2 $CaCl_2$, 4.2 $NaHCO_3$, 20 glucose and 15 HEPES buffer; pH=7.2-7.4). K^+ stimulation was carried out using a Tyrode's buffer containing either 30 or 45 mM K^+ (in mM: 88 or 103 NaCl, 45 or 30 KCl, 1 $MgCl_2$, 2 $CaCl_2$, 4.2 $NaHCO_3$, 20 glucose, and 25 HEPES buffer; pH=7.2-7.4).

Confocal imaging

Confocal imaging was conducted on an Olympus BX50WI microscope equipped with a 60x water immersion objective (NA=0.9 or 1.1) and a BioRad Radiance 2000 confocal scanhead (BioRad/Zeiss) using a 488 nm argon laser and/or a 543 nm helium-neon laser. Images were acquired using Lasersharp software either as xy images, z-stacks, or line scans (xt). Quantitative assessment of the image data was performed offline in Image J (NIH). Fluorescence were either calculated as absolute changes measured

in arbitrary units after background subtraction, or as fractional changes (%ΔF/F=100 x (F-$F_{initial}$)/($F_{initial}$-$F_{background}$)).

Total Internal Reflection Fluorescence Microscopy (TIFRM)

Dissociated hippocampal neurons (DIV 14-21) were labelled with LysoTracker or LAMP2-SEP and imaged using TIRFM (Cairn Research UK) with a 60X water immersion objective (Olympus; NA=0.90), CCD camera (Andor Luca) and Andor SOLIS software. TIRFM images were recorded over a time-course of 45-75 seconds with an exposure taken each second, during which a 45 mM K^+ Tyrode's solution was applied. Images were analysed using Image J as fractional changes (%ΔF/F=100 x (F-$F_{initial}$)/($F_{initial}$-$F_{background}$)).

Ca^{2+} imaging and electrophysiology

Hippocampal CA3 or CA1 pyramidal cells in acute or cultured hippocampal slices were recorded from using either sharp microelectrodes (70-120MΩ) or high-resistance patch electrodes (16-25 MΩ) to minimize intracellular dialysis. Sharp microelectrodes were tip filled with the Ca^{2+}-sensitive dye Oregon Green 488 BAPTA-1 (0.5-1 mM; Thermo Fisher) dissolved in 200 mM potassium acetate, and back-filled with 400 mM potassium acetate. Patch electrodes contained Oregon Green 488 BAPTA-1 (0.1-0.2 mM) dissolved in standard internal solution (in mM: 135 KGluconate, 10 KCl, 10 HEPES, 2 $MgCl_2$, 2 Na_2ATP and 0.4 Na_3GTP; pH=7.2-7.4). Cells were filled with fluorescent indicator, and regions of apical dendrite, within 100 µm of the soma, were visualised by confocal laser scanning microscopy using a 488 nm argon laser. The scan axis was aligned to bisect a dendrite of interest and line-scans consisting of 256 successive sweeps, at 2 ms intervals, were acquired, during which back-propagating action potentials (bpAPs) were triggered with somatic current injection (1-2 nA, 5-10 ms) and recorded with an AxocLAMP-2B amplifier (Axon Instruments). At least 3 bpAPs, evoked at 15-30 second intervals, were sampled both before and following pharmacological manipulation. In some experiments, 3 line scans were additionally acquired during a 200 ms depolarization pulse of 2-3nA, which depolarized the cell to >20 mV. Quantitative assessment of the image data was performed off-line in Image J (NIH). The amplitude of bpAP-evoked Ca^{2+} transients were calculated as percentage fractional change in fluorescence: %ΔF/F=100 x (F-$F_{initial}$)/($F_{initial}$-$F_{background}$). The decay constant (tau) was calculated by first peak-matching Ca^{2+} transients to control, and then fitting the falling phase of the transient (200 ms time window following its peak) with an exponential decay function. Membrane conductance was measured from a current pulse that was delivered throughout the experiment. Action potential amplitude and width at half-height were calculated using Axoclamp software.

VGCC current recordings

VGCC currents were recorded in whole-cell patch clamp from CA1 pyramidal neurons. Patch electrodes (4-6 MΩ electrodes) contained a cesium based internal solution (in mM: 115 Cs-methanesulphonate, 25 TEA-Cl, 10 HEPES, 4 MgATP, 1 EGTA and 0.4 Na_3GTP; pH=7.2-7.4). Recordings were conducted in standard ACSF solution, containing 2 mM $CsCl_2$, 1 mM 4-AP, and 2 µM TTX in order to block voltage-gated K^+ and voltage-gated Na^+ channels. Currents were recorded during 10 mV voltage steps, 400 ms in duration, from -70 mV to + 70 mV. For each voltage step, leak and capacitive currents were determined by averaging the currents recorded during 4 negative voltage steps, each with $1/4^{th}$ the amplitude of the test pulse, and subtracted from the test pulse. The liquid junction potential was experimentally measured and subtracted online. Capacitance was compensated by 70%. Access resistance (<20 MΩ) was monitored throughout the recordings. Experiments were omitted if access resistance increased greater than 25 MΩ or varied more than 20% during the recording.

NAADP-evoked Ca^{2+} transients

Lysosomal Ca^{2+} transients were evoked in hippocampal pyramidal neurons in slice culture. CA3 or CA1 neurons were loaded with NAADP (10-1000 nM) and Ca^{2+}-sensitive dye Oregon Green 488 BAPTA-1 (50-100 µM). Cells were imaged with confocal microscopy using a 488 nm argon line.

Regions of dendrite were scanned for 10 seconds at a time to record spontaneous NAADP-evoked Ca^{2+} transients. Experiments were conducted in the presence of 1 µM TTX to prevent activity-dependent Ca^{2+} signalling from contaminating lysosomal Ca^{2+} release.

Synaptogmin VII recombinant protein

The cytosolic domain (C-350 amino acids) of human synaptotagmin VII (sytVII) recombinant protein was commercially obtained (513376; Novoprolabs). The protein was purified and concentrated (100-200 µg/mL) in standard patch internal solution using protein concentrators (10kDa cut off; Thermofisher). sytVII was loaded into neurons by dialysis through a patch electrode (7-10 MΩ) for 5-10 minutes prior to the experiment. The patch was then removed except in structural plasticity experiments.

Structural plasticity and spine imaging

Structural plasticity was examined in hippocampal slice cultures. CA1 pyramidal neurons were recorded using a sharp patch electrode (16-25 MΩ) to minimize intracellular dialysis, and loaded with Alexa Fluor 488 (0.1-0.2 mM; Thermo Fisher) for visualization purposes. A dendritic spine with a head diameter of <1 µm within 100 µm of the soma was targeted for glutamate photolysis using a 405 nm laser (Photonics) equipped with a rapid shutter (LS6; Uniblitz). MNI glutamate (10 mM; Tocris) was focally delivered through a glass pipette (4-8 MΩ) using a picosprtizer (custom made) and uncaged in a ~1 µm-diameter spot above the spine head with a ~1 ms laser pulse. Laser intensity (0.5-2 mW) was adjusted to trigger a ~1 mV EPSP. Photolysis was paired with a 50 ms somatic current injection (2nA) in order to trigger 3-5 action potentials at 50-100 Hz, with the first action potential following uncaging by 7-10 ms. Pairing was repeated 60 times at 5 Hz. Images of the target and neighbouring spines were acquired with 512 x 512 resolution as a z-stack with a step size of 0.5 µm at -5, -2.5, 0, 1, 15, 30, 45, and 60 minutes post-pairing. In some experiments, freshly prepared natural human Cathepsin B (5 µg/mL; abcam) or recombinant human MMP-9 (5 µg/mL; Merck Millipore) was applied 10 minutes after the start of paired stimulation. Cathepsin B was bath applied whilst MMP-9 was locally perfused through a glass pipette (4-8 MΩ) using a picosprtizer (custom made). Fractional changes ($\%\Delta F/F=100 \times (F-F_{initial})/(F_{initial}-F_{background})$) in fluorescence of the spine head, standardized to that of the underlying dendrite, were used to estimate fractional changes in spine volume, as previously described (Matsuzaki et al., 2004, Kasai et al., 2008).

Fluorescein-based pH imaging

To examine drug-induced changes in intracellular pH, CA1 pyramidal neurons in hippocampal slice cultures were patched and loaded with 50 µM fluorescein (Sigma), which exhibits pH-dependent changes in fluorescence. Fluorescence was imaged in normal ACSF and following the addition of either a NH_4Cl buffer (in mM: 95 NaCl, 50 NH_4Cl, 16 $NaHCO_3$, 11 glucose, 2.5 KCl, 2-3 $CaCl_2$, 1-2 $MgCl_2$, 1.2 NaH_2PO_4; pH=7.5), a MES buffer (in mM: 130 NaCl, 30 MES, 16 $NaHCO_3$, 11 glucose, 2.5 KCl, 2-3 $CaCl_2$, 1-2 $MgCl_2$, 1.2 NaH_2PO_4; pH=6.5), or ACSF containing either GPN or monensin.

Lipofectamine transfection

Dissociated primary hippocampal cell cultures (DIV 7) were transfected with genetic constructs using Lipofectamine-2000 (Thermo Fisher) that had been pre-incubated with DNA at a ratio of 2:1. The complex of Lipofectamine and DNA was applied to cells for 1 hour in 1 mL of medium before being replaced with conditioned media. Cells were imaged at DIV 10-14.

Lysosomal labelling

To label lysosomes, dissociated neuronal cells were incubated with 50-75 nM LysoTracker Green or Red (Thermo Fisher) and/or 0.4% of fluoregenic Magic Red Cathepsin K and L substrates (ImmunoChemistry Technologies) in the dark for 1-2 hours at 37°C and 5% CO_2 prior to imaging, as per manufacturer's instructions. For visualization in cultured slice, single CA3 or CA1 neurons were loaded with Alexa Fluor 488 (0.1-0.2 mM) using a patch electrode.

Live-cell LAMP-2 immunolabelling

Live-cell immunolabelling of LAMP-2 was used to assess activity-dependent lysosomal fusion. Dissociated neuronal cultures (DIV 14-21) were first incubated with an unlabelled rabbit polyclonal antibody targeting the lumenal epitope of LAMP-2 (H-207; Santa Cruz Biotechnology) for 1-2 hours in order to block any LAMP-2 epitopes already present at the cell surface. The antibody was used at a concentration of 1:20, diluted in Tyrode's buffer with 2% fetal calf serum (FCS). Cultures were then washed with the same Tyrode's buffer with 2% FCS before a fluorescently-labelled variant of the same LAMP-2 antibody was applied at the same concentration (1:20). Fluorescent labelling was achieved by biotinylating the antibody (biotinylation kit; Abcam) and pre-treating it with streptavidin conjugated to Alexa Fluor 488 (1:100) (Thermo Fisher). Cultures were exposed to either standard Tyrode's buffer with 2% FCS, or to Tyrode's buffer containing 45 mM K^+ for 60 seconds. Cultures were then immediately placed on ice to prevent the endocytosis of fused lysosomes and subsequent enzymatic degradation of internalized antibodies. Cells were washed again with Tyrodes buffer containing 2% FCS and subsequently imaged using confocal microscopy in standard, ice-cold Tyrode's buffer.

The specificity of the LAMP-2 antibody was assessed by examining immunolabelling LAMP-2 knock-out (KO) cells. KO cells were generated by transfecting primary hippocampal cultures (DIV 7) with a CRISPR/Cas9 lentivector (K6833205; NBS Biologicals LTD) containing a targeting sequence (AGCCCGTAACCGGAGAGAGG) for rat LAMP-2. Cells were imaged using confocal microscopy 10 days later.

LAMP2-SEP

Superecliptic pHluorin (SEP) was amplified from template DNA, kindly provided by Dr Colin Akerman, (University of Oxford). NheI and BspE1 restriction sites were used to clone the SEP tag into the pEGFP-C1 vector (Clontech) in place of EGFP, creating a pSEP-C1 vector. LAMP-2 DNA was amplified from a mouse brain cDNA library (Life technologies) and subcloned into pSEP-C1 vector using EcoRI/SacII restriction sites tagging SEP to the N-terminus of LAMP-2.

Dissociated primary hippocampal cell cultures were transfected with LAMP2-SEP. The localization of LAMP2-SEP was assessed by loading cells with Magic Red Cathespin K and L fluorescent substrates (ImmunoChemistry Technologies) as per manufactures instructions, and examining the co-localization of red and green fluorescence with confocal microscopy. To assess the pH sensitivity of the construct, transfected cells were imaged in standard Tyrode's buffer, MES-based buffer (in mM: 128 NaCl, 5 KCl, 1 $MgCl_2$, 2 $CaCl_2$, 4.2 $NaHCO_3$, 20 glucose and 25 MES; pH=5.5), and NH_4Cl-based buffer (in mM: 78 NaCl, 50 NH_4Cl, 5 KCl, 1 $MgCl_2$, 2 $CaCl_2$, 4.2 $NaHCO_3$, 20 glucose and 25 HEPES buffer; pH=7.4).

For assessment of activity-dependent lysosome fusion, LAMP2-SEP transfected neurons were imaged either using TIRFM or confocal microscopy with a 60X water immersion objective (NA=0.90), CCD camera (Andor Luca) and Andor SOLIS software. TIRFM images were recorded over a time-course of 75 seconds with an exposure taken each second, during which a 45 mM K^+ Tyrode's solution was applied. Prior to imaging, LAMP2-SEP fluorescence was photobleached to minimize basal fluorescence in the dendrites. In some experiments, activity was driven electrically by using field stimulation (40 V, 1 ms) to deliver 300 action potentials at 5Hz via a pair of platinum bipolar electrodes, spaced 1 cm apart. Bafilomycin (1 µM) was acutely applied during stimulation to prevent re-acidification of retrieved lysosomes.

Cathepsin B assays

To assess Cathepsin B release in dissociated hippocampal neurons, cells were first loaded with 0.4% Magic Red Cathepsin B fluoregenic substrate (ImmunoChemistry Technologies) and incubated for 1-2 hours at 37°C and 5% CO_2, as per manufacturer's instructions. To confirm correct loading of the

substrate, the distribution of labelled puncta was examined in neurons transfected with Cathepsin B-GFP (pCT-Lyso-GFP; Cambridge Bioscience), and were found to co-localize strongly with GFP labelled compartments. Following loading, cultures were then stimulated with 45 mM K^+ Tyrode's solution and puncta were examined for loss of Magic Red fluorescence using confocal microscopy. Puncta exhibiting a fluorescence loss greater than 3 standard deviations from the average fluorescence recorded in unstimulated control cultures were said to have undergone fusion. To examine the distribution of Cathepsin B puncta in the vicinity of synapses, experiments were repeated following live-cell immunolabelling of GluA1-containg receptors. Dissociated neurons were incubated with rabbit anti-GluA1 antibody (PC246 Merck Millipore; 1:50 in Tyrode's solution containing 2% FCS) for 1 hour. Cultures were then washed with Tyrode's solution containing 2% FCS and incubated with goat anti-rabbit secondary antibodies conjugated to Alexa Fluor 488 (Abcam: ab150129; 1:50 in Tyrode's solution containing 2% FCS) for 1 hour, and then washed with Tyrode's solution containing 2% FCS. The shortest distance of Cathepsin B puncta to the nearest GluA1-stained puncta was calculated using ImageJ and Excel macros.

To examine activity-dependent Cathepsin B release in hippocampal slice cultures, slices were first washed in Tyrode's solution and then incubated for 2 hours at 37°C and 5% CO_2 with 200 µL Tyrodes solution. The Tyrodes solution contained either TTX (2 µm) to minimize neuronal activity or 30 mM K^+ to augment neuronal activity. Following the incubation, Cathepsin B levels were assessed in 100µL of the supernatant using a colourimetric human Cathepsin B ELISA kit (ab119684; Abcam), as per manufacture's instructions. Absorbance was measured at 450 nm using a plate reader and expressed in arbitrary units after subtraction of blank controls.

To examine Cathepsin B activity in hippocampal slice cultures, slices were first washed in Tyrode's solution and then incubated for 2 hours at 37°C and 5% CO_2 in 200 µL Tyrodes solution containing the Cathepsin B fluoregenic substrate Z-Arg-Arg-AMC (1:200; JA7740; Merck Millipore). During the incubation, slices were treated either with TTX (2 µm) to minimize neuronal activity or 30 mM K^+ to augment neuronal activity. Following the incubation, fluorescence of 100 µL of the supernatant was assessed using a plate reader (360 nm excitation/ 460 nm emission). Fluorescence was expressed in arbitrary fluorescent units (AFU) after subtraction of blank controls.

MMP activity assay

Matrix metalloproteinase (MMP) activity was assessed using a MMP fluorogenic substrate (PEPDAB0502; Biozyme), which despite being advertised as a specific MMP-9 fluoregenic substrate, actually shows relatively broad selectivity for a number of MMPs (see (Miller et al., 2011)). Cultured hippocampal slices were incubated with 5 µM of the substrate at 37°C and 5% CO_2 for 2 hours in 200 µL of normal Tyrode's solution or a 30 mM K^+ Tyrode's solution. Fluorescence of 100 µL of solution was assessed using a plate reader (480 nm excitation/520 nm emission). Fluorescence was expressed in arbitrary fluorescent units (AFU) after subtraction of blank controls. For assessment of MMP-9 activity under a particular experimental condition, 100 nM of MMP-9 Inhibitor-I was included in half of the experiments. The average fluorescence in the absence and presence of the inhibitor was subtracted to obtain a measure of MMP-9-associated activity. Although MMP-9 Inhibitor I is a potent inhibitor of MMP-9 (K_d=5 nM), it has been reported to act on MMP-13, albeit with 20-fold less affinity (Levin et al., 2001). To rule out the confounding effects of MMP-13, we repeated our experiments in the presence of an MMP-13 selective inhibitor (100 nM WAY 170523). Under these conditions we were still able to robustly detect MMP-9 activity (AFU=1317.91±318.87; n=11 slices; $p<0.01$) following K^+ stimulation, which we were still able to abolish this activity using either CA-074 (AFU=-235.70±377.55; n=8 slices; vs. K^+: $p<0.01$) or NED-19 (AFU=152.32±286.55; n=16 slices; vs. K^+: $p<0.01$).

Western Blot

MMP-9 and TIMP-1 expression levels were examined in cultured hippocampal slices. Slices were first washed in Tyrode's solution and then incubated for 2 hours at 37°C and 5% CO_2 in 200 µL Tyrodes solution, either containing TTX (2 µm) to minimize neuronal activity or 30 mM K^+ to augment neuronal activity. Slices were then homogenised in 24 µL of RIPA buffer. The lysate was boiled at 95 ˚C in 4x Laemmli sample buffer (Biorad) containing 10% 2-mercaptoethanol for 5 minutes and loaded onto a 4-20% polyacrylamide precast gel (Biorad) in standard SDS-containing Tris-glycine running buffer. Since MMP-9/TIMP-1 ratios were being analyzed instead of absolute MMP-9 or TIMP-1 levels, the concentration of protein loaded per well was not controlled for. Gels were then transferred onto nitrocellulose membranes (Whatman) in a Tris-glycine transfer buffer (ThermoFisher). Membranes were blocked with 3% BSA in TBS-T (Tris-buffered saline + Tween 20) and incubated with rabbit primary antibodies against MMP-9 (1:300; ab13458; Merck Millipore) and TIMP-1 (1:200; H-150; Santa Cruz) for 1 hour at room temperature. Proteins were detected with a goat-anti-rabbit IgG conjugated to IRDye® 800 (Li-Cor, 1:20000, 1 h at room temperature). Images were analyzed ImageJ software (National Institutes of Health). Fluorescence intensity of TIMP-1 and MMP-9 protein bands were calculated following background subtraction, and expressed as a ratio (MMP-9/TIMP-1).

Drugs

Ryanodine (20 µM final; Abcam), thapsigargin (15 µM final; Abcam), FCCP (1 µM final; Tocris), GPN (200 µM final; Sigma), trans-NED-19 (10-100 µM final; Tocris), bafilomycin (1-4 µM final; Abam), CA-074 (1 µM final; Tocris), WAY 170523 (100 nM final; Tocris), and MMP-9 Inhibitor-1 (100 nM final; Calbiochem) were dissolved in dimethyl sulfoxide (DMSO). Monensin (20 µM final; Sigma) was dissolved in 50% DMSO/50% methanol. Trolox (1 mM final; Sigma) was dissolved in 100% ethanol. NAADP (10 µM-10 mM final; Sigma), TTX (1-2 µM final; abcam), 4-AP (100 µM final; Sigma); $NiCl_2$ (100 µM final; Sigma), and $CdCl_2$ (100 µM final; Sigma) were dissolved in water. EGTA (1 mM final; Sigma) and ascorbic acid (0.2 mM) were dissolved directly in ACSF. Drugs were bath applied for 10 minutes prior to imaging with the exception of NAADP, which was applied in the internal solution and allowed to dialyze into the cell for 10-20 minutes prior to imaging, and bafilomycin and NED-19, which were applied for at least 1 hours prior to imaging unless otherwise indicated. 0 Ca^{2+} conditions were achieved by washing slices with ACSF solution comprising of 145 mM NaCl, 16 mM $NaHCO_3$, 11 mM glucose, 2.5 mM KCl, 0 mM $CaCl_2$, 4 mM $MgCl_2$, and 1.2 mM NaH_2PO_4, buffered with 1 mM EGTA.

Supplemental References

Erecinska M, Dagani F, Nelson D, Deas J, Silver IA (1991) Relations between intracellular ions and energy metabolism: a study with monensin in synaptosomes, neurons, and C6 glioma cells. J Neurosci 11:2410-2421.

Freundt EC, Czapiga M, Lenardo MJ (2007) Photoconversion of Lysotracker Red to a green fluorescent molecule. CELL RESEARCH-ENGLISH EDITION- 17:956.

Kasai H, Tanaka JI, Horiike Y, Matsuzaki M, Miyazaki T, Ellis-Davies GCR (2008) Protein synthesis and neurotrophin-dependent structural plasticity of single dendritic spines. Science 319:1683-1687.

Levin JI, Chen J, Du M, Hogan M, Kincaid S, Nelson FC, Venkatesan AM, Wehr T, Zask A, DiJoseph J, Killar LM, Skala S, Sung A, Sharr M, Roth C, Jin G, Cowling R, Mohler KM, Black RA, March CJ, Skotnicki JS (2001) The discovery of anthranilic acid-Based MMP inhibitors. Part 2: SAR of the 5-position and P11 groups. Bioorganic & Medicinal Chemistry Letters 11:2189-2192.

Matsuzaki M, Honkura N, Ellis-Davies GC, Kasai H (2004) Structural basis of long-term potentiation in single dendritic spines. Nature 429:761-766.

Miller MA, Barkal L, Jeng K, Herrlich A, Moss M, Griffith LG, Lauffenburger DA (2011) Proteolytic Activity Matrix Analysis (PrAMA) for simultaneous determination of multiple protease activities. Integrative biology : quantitative biosciences from nano to macro 3:422-438.
