# Supplementary figures and images for: Activity-Dependent Exocytosis of Lysosomes Regulates the Structural Plasticity of Dendritic Spines
Source: Neuron. 2017 Jan 4;93(1):132–46. doi: 10.1016/j.neuron.2016.11.013 (PMC5222721; doi:10.1016/j.neuron.2016.11.013)

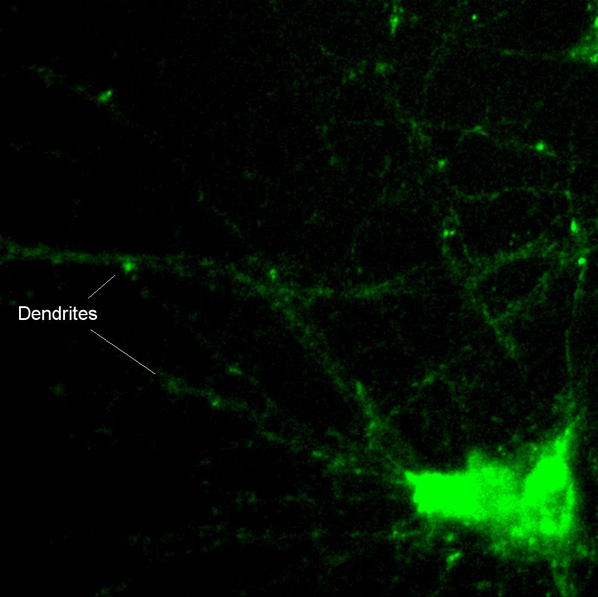

Supplement: Movie S1. Imaging Activity-Dependent Mobilization of Lysosomes at the Cell Surface, Related to Figure 4 — Sample video clip of TIRF imaging experiments of lysosomes shown in Figure 4A. Two dissociated hippocampal neurons (soma are on the bottom right) were loaded with LysoTracker (green fluorescence) to label lysosomes. The experiment starts with a baseline recording. After which, the culture is stimulated with 45 mM K+; this period is denoted with a label reading “K+” on the top left corner of the video. During stimulation, there is a rapid emergence of fluorescence puncta throughout the dendritic arbour, which are not seen during baseline imaging. Frames were acquired at the rate of 1/s and are played at a rate of 4/s. [file mmc2.jpg]

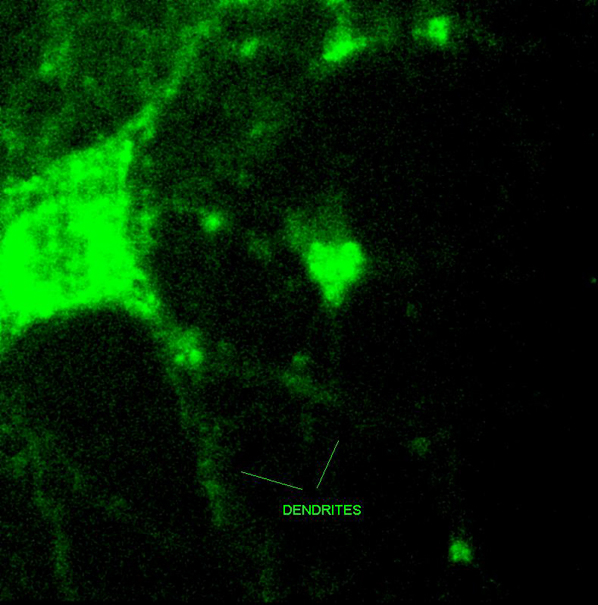

Supplement: Movie S2. Imaging Activity-Dependent Lysosomal Fusion Using LAMP2-SEP, Related to Figure 5 — Sample video clip from experiments shown in Figure 5B. A dissociated hippocampal neuron (soma on the top left) was transfected with LAMP2-SEP (green fluorescence). The experiment starts with a period of photobleaching (denoted by the label “photobleach” on the top right corner of the clip), in order to eliminate baseline fluorescence of LAMP-2 that is already present on the cell surface. Following photobleaching, there is a baseline recording, after which the culture is stimulated with 45 mM K+ (denoted by the label “K+” on the top right corner of the clip). During stimulation, there is a rapid emergence of fluorescence puncta throughout the dendritic arbour, which are not seen during baseline imaging. Frames were acquired at the rate of 1/s and are played at a rate of 4/s. [file mmc3.jpg]
