# Supplementary material for: Activity-Dependent Exocytosis of Lysosomes Regulates the Structural Plasticity of Dendritic Spines
Source: Neuron. 2017 Jan 4;93(1):132–46. doi: 10.1016/j.neuron.2016.11.013 (PMC5222721; doi:10.1016/j.neuron.2016.11.013)
Supplement: Document S2. Article plus Supplemental Information [file mmc4.pdf]

Neuron

Article

Activity-Dependent Exocytosis of Lysosomes Regulates the Structural Plasticity of Dendritic Spines

Highlights

- Back-propagating action potentials induce Ca^{2+} release from lysosomes in neurons
- Lysosomal Ca^{2+} release triggers exocytosis of the lysosomal protease Cathepsin B
- Cathepsin B maintains activity-dependent dendritic spine growth by activating MMP-9

Authors

Zahid Padamsey, Lindsay McGuinness, Scott J. Bardo, ..., Anne Hedegaard, Michael L. Hart, Nigel J. Emptage

Correspondence

nigel.emptage@pharm.ox.ac.uk

In Brief

Padamsey et al. find that back-propagating action potentials trigger Ca^{2+} release from lysosomes in hippocampal pyramidal dendrites. This Ca^{2+} release results in the exocytosis of the lysosomal protease Cathepsin B, which activates MMP-9 to maintain activity-dependent spine growth.

Padamsey et al., 2017, Neuron *93*, 132–146
January 4, 2017 Crown Copyright © 2017 Published by Elsevier Inc.
http://dx.doi.org/10.1016/j.neuron.2016.11.013

Activity-Dependent Exocytosis of Lysosomes Regulates the Structural Plasticity of Dendritic Spines

Zahid Padamsey,[1] Lindsay McGuinness,[1] Scott J. Bardo,[1] Marcia Reinhart,[1] Rudi Tong,[1] Anne Hedegaard,[1] Michael L. Hart,[1] and Nigel J. Emptage[1,2,*]
[1]Department of Pharmacology, University of Oxford, Mansfield Road, Oxford, OX1 3QT, UK
[2]Lead Contact
*Correspondence: nigel.emptage@pharm.ox.ac.uk
http://dx.doi.org/10.1016/j.neuron.2016.11.013

SUMMARY

Lysosomes have traditionally been viewed as degradative organelles, although a growing body of evidence suggests that they can function as Ca^{2+} stores. Here we examined the function of these stores in hippocampal pyramidal neurons. We found that back-propagating action potentials (bpAPs) could elicit Ca^{2+} release from lysosomes in the dendrites. This Ca^{2+} release triggered the fusion of lysosomes with the plasma membrane, resulting in the release of Cathepsin B. Cathepsin B increased the activity of matrix metalloproteinase 9 (MMP-9), an enzyme involved in extracellular matrix (ECM) remodelling and synaptic plasticity. Inhibition of either lysosomal Ca^{2+} signaling or Cathepsin B release prevented the maintenance of dendritic spine growth induced by Hebbian activity. This impairment could be rescued by exogenous application of active MMP-9. Our findings suggest that activity-dependent exocytosis of Cathepsin B from lysosomes regulates the long-term structural plasticity of dendritic spines by triggering MMP-9 activation and ECM remodelling.

INTRODUCTION

Lysosomes are classically viewed as degradative organelles (Luzio et al., 2007b). Several studies, however, have demonstrated that lysosomes, along with other related acidic organelles, comprise additional sources of intracellular Ca^{2+}. Notably, these sources are distinct from the endoplasmic reticulum (ER) and mitochondria (Galione, 2015). In this paper, we use the term lysosome to collectively refer to the lysosome and its related acidic organelles.

Ca^{2+} signaling from lysosomes has now been described in a number of cell types (Galione et al., 2010, 2015). Ca^{2+} storage in the lysosome requires an H^+ gradient, which is generated by a vacuolar H^+-ATPase (V-ATPase), and some form of Ca^{2+}/H^+ exchange (Morgan et al., 2011). Ca^{2+} release from the lysosomes requires the second messenger nicotinic acid adenine dinucleotide phosphate (NAADP), which is synthesized, most likely by ADP-ribosyl cyclases, in response to cell-specific physiological stimuli (Galione et al., 2010, 2015). NAADP-dependent Ca^{2+} efflux from lysosomes is thought to occur either via transient receptor potential cation channels of the mucolipin 1 family (TRPML1) or via two-pore channels (TPC1/TPC2) (Guse, 2012), with the weight of evidence currently suggesting that NAADP acts via TPCs, most likely via an NAADP-associated binding protein (Morgan and Galione, 2014; Morgan et al., 2015b).

NAADP-dependent lysosomal Ca^{2+} signaling is likely to play a role in neurons. NAADP binding sites have been found throughout the brain (Patel et al., 2000), and NAADP synthesis and lysosomal Ca^{2+} release in neurons can be triggered by exogenous stimulation with glutamate (Pandey et al., 2009). Moreover, application of NAADP elicits Ca^{2+} release in brain microsomes (Bak et al., 1999), promotes neuronal differentiation in PC12 cells (Brailoiu et al., 2006), augments neurite outgrowth in developing cortical neurons (Brailoiu et al., 2005), drives membrane depolarization in medullary neurons (Brailoiu et al., 2009), and increases Ca^{2+} influx through N-type voltage-gated Ca^{2+} channels (VGCCs) in cultured hippocampal neurons (Hui et al., 2015). Lysosomes have also been found in axonal boutons, and pharmacologically induced Ca^{2+} release from these organelles can drive spontaneous neurotransmitter release (Brailoiu et al., 2003; McGuinness et al., 2007). Lysosomal Ca^{2+} release, however, has not been investigated in postsynaptic structures or in the context of synaptic plasticity.

In addition to mediating Ca^{2+} signaling, the lysosome itself can also undergo Ca^{2+}-dependent fusion, both with other organelles and with the plasma membrane (Luzio et al., 2007b). In the latter case, lysosomal exocytosis can be important in some cell types for the secretion of chemical signals or enzymes (Blott and Griffiths, 2002; Zhang et al., 2007; Galione et al., 2010; Davis et al., 2012; Galione, 2015) or for promoting lipid turnover and facilitating membrane growth and repair (Reddy et al., 2001; Blott and Griffiths, 2002; Luzio et al., 2007b). Although not well studied, lysosomal fusion has been reported in developing hippocampal and sympathetic neuronal cultures, where it regulates the surface expression of N-type VGCCs and the outgrowth of neurites, respectively (Arantes and Andrews, 2006; Hui et al., 2015). However, it is not clear what physiological trigger drives lysosomes to fuse with the plasma membrane or the significance of this fusion for information processing in neurons.

 Crown Copyright © 2017 Published by Elsevier Inc.
This is an open access article under the CC BY license (http://creativecommons.org/licenses/by/4.0/).

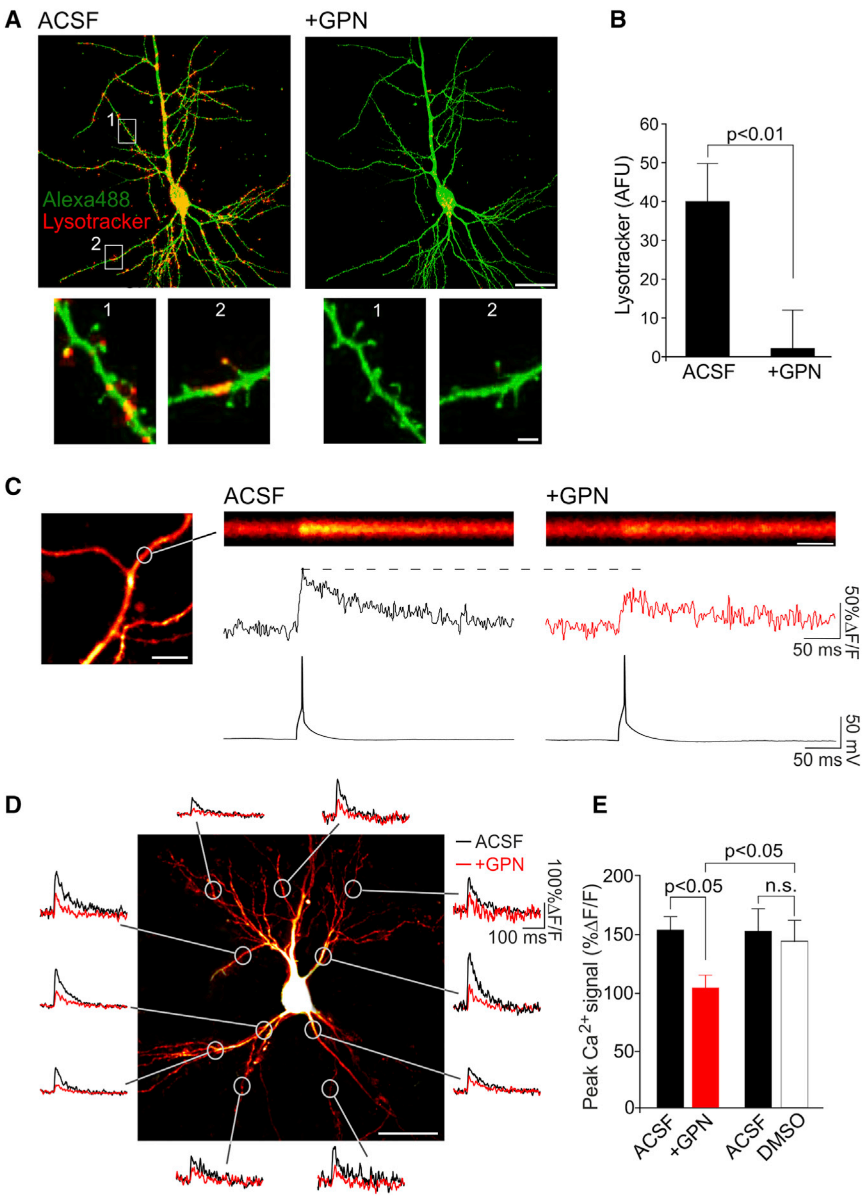

Figure 1. Lysosomes Contribute to bpAP-Evoked Dendritic Ca^{2+} Signaling

(A) Top: CA1 neuron loaded with Alexa 488 (green) and stained with LysoTracker (red) (scale bar, 10 μm). Disruption of the lysosomal membrane with GPN abolished LysoTracker staining. Regions of interest (white boxes) are magnified below (scale bar, 2 μm).

(B) Average LysoTracker staining in ACSF (artificial cerebrospinal fluid) and GPN (n = 5 cells/condition). Significance was assessed with a Mann-Whitney test.

(C) Example bpAP-evoked Ca^{2+} transient in CA1 apical dendrites loaded with OGB-1 (scale bar, 10 μm). Laser scanning was restricted to a line across a region of interest (circle). The resulting line scan (scale bar, 50 ms), along with its quantification (%ΔF/F) is shown time-locked with electrophysiological recordings. GPN reduced the bpAP-evoked Ca^{2+} influx.

(D) CA1 neuron loaded with OGB-1. Ca^{2+} transients evoked before (black traces) and after (red traces) the addition of GPN are shown for regions of interest (scale bar, 20 μm).

(E) Average data (n = 7–18 cells/condition). Significance was assessed with Kruskal-Wallis and post hoc Dunn's tests. Error bars represent SEM. See also Figures S1 and S2.

Here we examined the role of lysosomes in hippocampal pyramidal neurons. We found that lysosomal Ca^{2+} signaling and lysosomal fusion with the plasma membrane were driven by back-propagating action potentials (bpAPs) and were necessary for the long-term structural plasticity of dendritic spines.

RESULTS

Lysosomes Contribute to bpAP-Evoked Dendritic Ca^{2+} Signaling

We began by examining the distribution of lysosomes in hippocampal pyramidal neurons. To do so, we incubated hippocampal slice cultures with LysoTracker red (50 nM, 40–60 min), a fluorescent dye that preferentially labels lysosomal compartments (Chazotte, 2011). We found LysoTracker-labeled puncta throughout the dendritic arbor of CA3 and CA1 neurons and in some dendritic spines (Figure 1A). To confirm that LysoTracker fluorescence was specific for lysosomes, we used glycyl-L-phenylalanine 2-naphthylamide (GPN, 200 μM, 5–10 min) to selectively disrupt lysosomal compartments. GPN is a cell-permeable substrate for the lysosome-specific enzyme Cathepsin C. When cleaved, GPN becomes membrane-impermeable and exerts an osmotic effect that disrupts the lysosomal membrane, leading to the leakage of small molecules out of the organelle (Penny et al., 2014). We found that addition of GPN abolished the vast majority of LysoTracker fluorescence in cells (n = 5 cells; Mann-Whitney test, $p < 0.01$; Figure 1B), suggesting that LysoTracker was predominantly labeling lysosomal compartments. Notably, GPN-induced disruption of the lysosomal membrane was not accompanied by any detrimental effects on neuronal morphology (Figure 1A), resting membrane potential, conductance, and intracellular pH (Figures S1 and S2).

We next examined whether lysosomes contributed to dendritic Ca^{2+} signaling during neuronal activity. We loaded CA3 and CA1 neurons with the Ca^{2+}-sensitive dye Oregon Green BAPTA-1 (OGB-1) and imaged dendritic Ca^{2+} in response to single bpAPs (Figure 1C). bpAP-evoked Ca^{2+} influx is thought to be predominantly, if not exclusively, mediated by VGCCs (Jaffe et al., 1992; Christie et al., 1995; Spruston et al., 1995; Helmchen et al., 1996). However, we found that GPN, despite

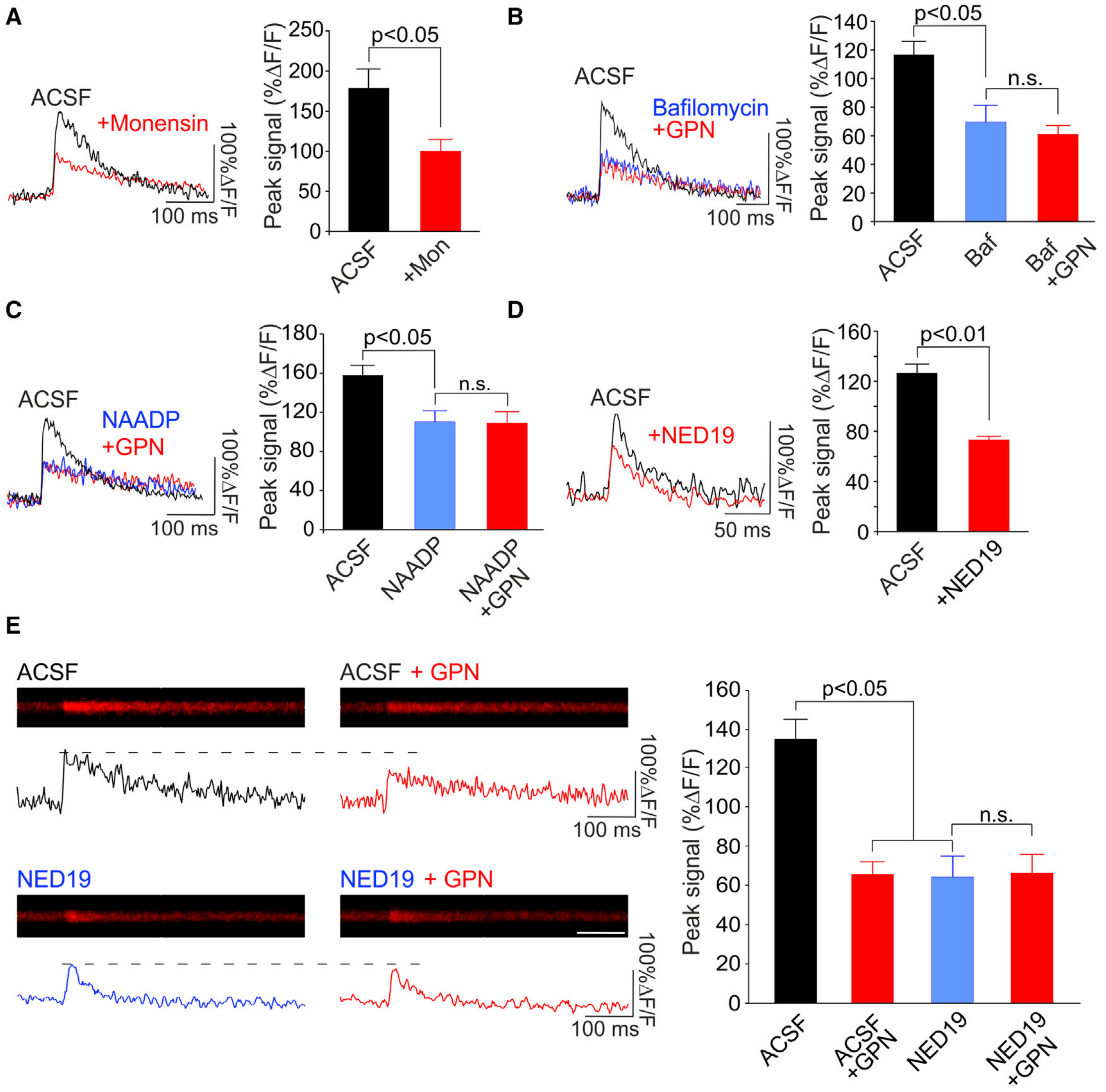

Figure 2. Lysosomal Ca^{2+} Release Requires NAADP

(A–D) Left: sample Ca^{2+} transients evoked by single bpAPs in CA1 apical dendrites. Ca^{2+} transients were reduced following inhibition of lysosomal Ca^{2+} signaling by various reagents. Right: average peak Ca^{2+} signal measured across conditions (n = 5–10 cells/condition). Significance was assessed with Mann-Whitney tests (single comparisons) or Kruskal-Wallis and post hoc Dunn's tests (multiple comparisons).

(E) Left: example of line scan images (scale bar, 100 ms) and quantified traces (%ΔF/F) of Ca^{2+} signals recorded in CA1 apical dendrites in acute hippocampal slices. Right: average peak fluorescence (n = 6–7 cells/condition). Ca^{2+} transients were reduced by GPN and NED-19. Significance was assessed with Kruskal-Wallis and post hoc Dunn's tests.

Error bars represent SEM. See also Figures S1–S5.

having no effect on the action potential waveform (Figure S1), significantly reduced the Ca^{2+} rise evoked by a single bpAP (in %ΔF/F; artificial cerebrospinal fluid [ACSF], 153 ± 11; GPN, 103 ± 10; n = 18 cells; post hoc Dunn's test, $p < 0.01$; Figures 1C–1E); these reductions were found throughout the dendritic arbor (Figure 1D) and were also present in acute hippocampal slices (n = 6 cells; post hoc Dunn's test, $p < 0.05$; Figures 2E and 2F). In contrast, application of vehicle control (DMSO) alone had no effect on bpAP-evoked Ca^{2+} transients (n = 7 cells; post hoc Dunn's test, p = 0.99; Figure 1E).

Lysosomal Ca^{2+} Release Requires NAADP

To confirm that neuronal activity could trigger Ca^{2+} release from the lysosomes, we used additional means of pharmacologically impairing lysosomal Ca^{2+} signaling (Galione, 2015; Morgan et al., 2015a; Figure 8B). We started by specifically inhibiting lysosomal Ca^{2+} storage, which requires a proton gradient to be established by V-ATPases (Morgan et al., 2011). To disrupt this gradient, we bath-applied the membrane-permeable Na^+/H^+ ionophore monensin (20 μM, 5–10 min). Monensin reduced the bpAP-evoked Ca^{2+} transient (in %ΔF/F; ACSF, 179 ± 24; monensin, 100 ± 14; n = 6 cells; Mann-Whitney test, $p < 0.05$; Figure 2A)

despite having no effect on action potential waveform (Figure S1). However, as previously reported, application of monensin resulted in membrane hyperpolarization (Figure S1), likely related to a modest alkalization of intracellular pH (Figure S2; Erecińska et al., 1991). Therefore, we conducted additional experiments in which lysosomal Ca^{2+} storage was disrupted using the V-ATPase inhibitor bafilomycin. Given the poor membrane permeability of the drug, we pre-incubated slices with bafilomycin (4 μM, 1–2 hr). This had no effect on the action potential waveform or on the resting membrane potential (Figure S1) but caused a significant decrease in the bpAP-associated Ca^{2+} signal that was comparable in magnitude to the decrease obtained in monensin (n = 6 cells/condition; bafilomycin versus control; post hoc Dunn's test, $p < 0.05$; Figure 2B). Notably, subsequent addition of GPN resulted in no further reduction in the Ca^{2+} signal (n = 6 cells; bafilomycin versus GPN, post hoc Dunn's test, p = 0.99; Figure 2B), suggesting that the effects of bafilomycin were due to its inhibition of the proton gradient in lysosomes as opposed to other organelles.

We next inhibited lysosomal Ca^{2+} release. Lysosomal Ca^{2+} release requires NAADP, which most likely acts on TPCs (Morgan et al., 2015b). Although low concentrations of NAADP (10–100 nM) evoke Ca^{2+} efflux from the lysosome, higher concentrations (>100 μM) inhibit it via desensitization of the Ca^{2+} release machinery (Berg et al., 2000; Masgrau et al., 2003). Consistent with this, we found that a concentration of 1 mM NAADP in the patch electrode could inhibit lysosomal Ca^{2+} release in hippocampal neurons (Figure S3). We also found that this concentration of NAADP reduced the bpAP-evoked Ca^{2+} signal (in %ΔF/F; ACSF, 158 ± 10; NAADP desensitization, 110 ± 11; n = 5 cells; post hoc Dunn's test, $p < 0.05$; Figure 2C) and occluded the effects of GPN (n = 5 cells; NAADP versus GPN, post hoc Dunn's test, p = 0.94; Figure 2C). Similar reductions in Ca^{2+} influx were also observed with the cell-permeable NAADP antagonist NED-19 (100 μM, 30-to 60-min pre-incubation; n = 10 cells; Mann-Whitney test, $p < 0.01$; Figure 2D). NED-19 also decreased the bpAP-associated Ca^{2+} signal in acute hippocampal slices (n = 6 cells; post hoc Dunn's test, $p < 0.05$; Figure 2E), and, again, such reductions occluded the effects of GPN (n = 6 cells; NED-19 versus GPN, post hoc Dunn's test, p = 0.48; Figure 2E).

Notably, drug-induced decreases in bpAP-evoked Ca^{2+} release did not result from direct inhibition of VGCC currents (Figure S4) or by affecting Ca^{2+} handling (Figure S5) but were likely due to inhibition of lysosomal Ca^{2+} signaling itself.

Ca^{2+} Influx through VGCCs Triggers Lysosomal Ca^{2+} Release

Although NAADP is a necessary trigger for lysosomal Ca^{2+} release, it alone does not explain how an electrical signal, such as a bpAP, triggers Ca^{2+} release from the lysosome. We hypothesized that an additional voltage-sensitive mechanism that gates or modulates lysosomal Ca^{2+} release must be present. One possibility is that the lysosomal Ca^{2+} release machinery may be directly voltage-sensitive (Rybalchenko et al., 2012; Cang et al., 2014; Pitt et al., 2014). Alternatively, lysosomes may sense voltage indirectly via functional coupling with plasma membrane voltage-gated channels. This coupling may be direct, as in the case of the mechanical coupling between ryanodine receptors and VGCCs in striated muscle (Sandow, 1952). This coupling may also be indirect and mediated by a second messenger such as Ca^{2+}. Indeed, it is known that Ca^{2+} can trigger NAADP-dependent lysosomal Ca^{2+} release (Patel et al., 2001; Morgan et al., 2013), potentially by directly activating TPCs (Pitt et al., 2010. 2014). Thus, it is possible that Ca^{2+} influx from VGCCs during bpAPs may trigger Ca^{2+} release from lysosomes.

To distinguish between these possibilities, we first attempted to image lysosomal Ca^{2+} release in the presence of Ni^{2+} (100 μM) and Cd^{2+} (100 μM) to inhibit VGCCs. This would determine whether the lysosomes could release Ca^{2+} independent of VGCC activity. Under these conditions, we could not detect any Ca^{2+} transients, either in response to a single bpAP (n = 6 cells; post hoc Dunn's test, p = 0.99; Figures 3A and 3B) or to a more potent, 200-ms step depolarization to >20 mV (n = 6 cells; post hoc Dunn's test, p = 0.99; Figures 3A and 3B) compared with unstimulated controls (n = 6). These findings suggest that membrane voltage alone cannot elicit lysosomal Ca^{2+} release and that lysosomal Ca^{2+} signaling requires VGCC activation. To examine specifically whether it was the direct mechanical opening of VGCCs or VGCC-mediated Ca^{2+} influx that was triggering lysosomal Ca^{2+} release, we repeated our experiments in 0 Ca^{2+} ACSF, which prevents VGCC-mediated Ca^{2+} influx but, unlike bath application of Ni^{2+} and Cd^{2+}, does so without potential interference of the mechanical opening and closing of VGCCs. Again, under these conditions, we were unable to detect any bpAP-evoked dendritic Ca^{2+} transients, either in response to a single bpAP (n = 5 cells; post hoc Dunn's test, p = 0.99; Figure 3B) or to a step depolarization to >20 mV (n = 5 cells; post hoc Dunn's test, p = 0.99; Figure 3B) compared with unstimulated controls (n = 6). As a positive control, to ensure that our experimental setup was sensitive enough to detect lysosomal Ca^{2+} transients if present, we included low, physiologically relevant concentrations of NAADP (10–100 nM) in our recording electrode to directly trigger Ca^{2+} release from the lysosomes (Calcraft et al., 2009; Figure S3). Under these conditions, we could reliably detect small and rapid Ca^{2+} transients throughout the dendritic arbor that could be abolished by the NAADP antagonist NED-19. Collectively, our findings suggest that Ca^{2+} influx through VGCCs triggers lysosomal Ca^{2+} release. In this way, lysosomal Ca^{2+} stores are indirectly coupled to VGCCs, enabling them to be mobilized by changes in membrane voltage that occur during bpAPs.

In many cell types, lysosomal Ca^{2+} release is functionally coupled to Ca^{2+} release from the ER as opposed to VGCCs (Patel et al., 2001; Galione et al., 2010; Morgan and Galione, 2014). Previous studies, however, have demonstrated that the ER does not contribute to bpAP-evoked Ca^{2+} transients (Markram et al., 1995; Emptage et al., 1999; Kovalchuk et al., 2000), arguing against lysosome-ER coupling in neuronal dendrites. In line with this, we found that blocking ER Ca^{2+} signaling with either ryanodine (20 μM, 20 min) or thapsigargin (15 μM, 20 min) had no effect on the amplitude of bpAP-evoked Ca^{2+} transients (n = 5 cells/condition; Kruskal-Wallis test, $p > 0.05$; Figures 3C and 3D). In a subset of experiments with thapsigargin, subsequent addition of GPN resulted in the expected reduction of

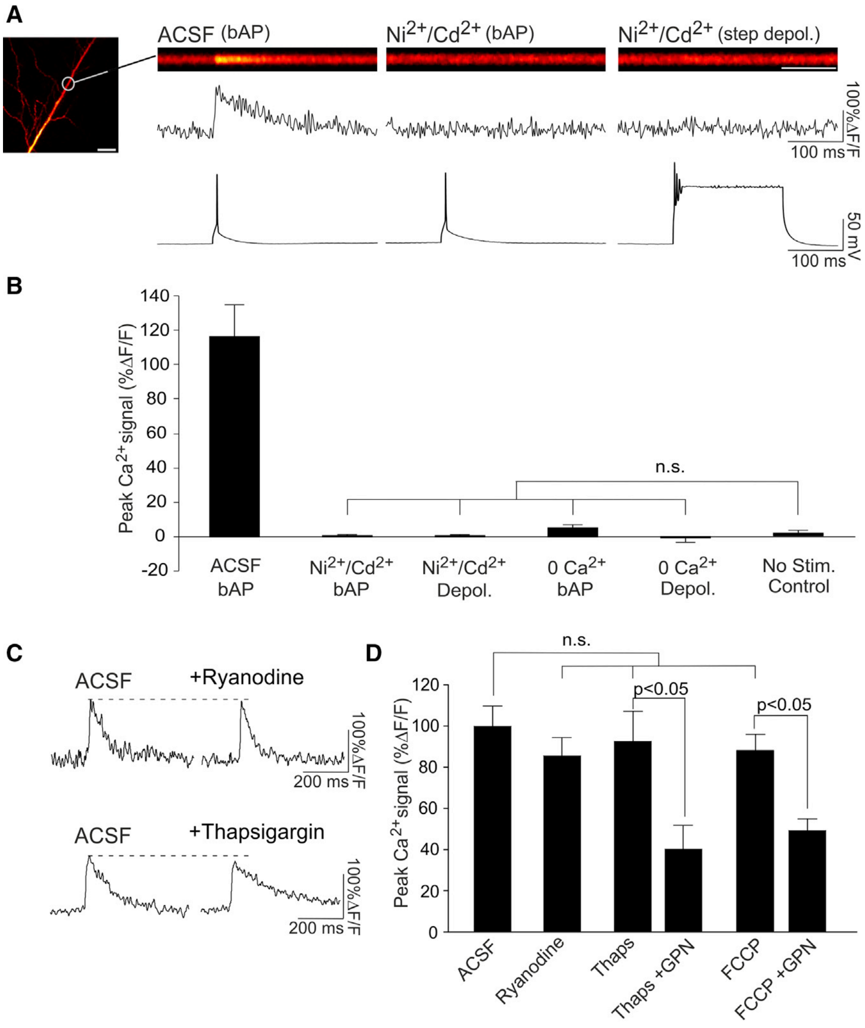

Figure 3. Ca^{2+} Influx through VGCCs Triggers Lysosomal Ca^{2+} Release

(A) Example line scan images (scale bar, 100 ms) and quantified traces (%ΔF/F) of bpAP-evoked Ca^{2+} transients in CA1 apical dendrites (scale bar, 10 μm) time-locked to electrophysiological recordings. Ni^{2+} and Cd^{2+} abolished Ca^{2+} influx evoked either by a single bpAP or a 200-ms step depolarization to >20 mV.

(B) Average data. Ca^{2+} transients were abolished by inhibition of VGCC-mediated Ca^{2+} influx by Ni^{2+}/Cd^{2+} or 0 Ca^{2+} and did not significantly differ from transients recorded in the absence of stimulation (no stim. control) (n = 5–6 cells/condition).

(C) Sample bpAP-evoked Ca^{2+} transients shown before and after the presence of ryanodine or thapsigargin to inhibit Ca^{2+} signaling from the ER.

(D) Average peak Ca^{2+} transients. Inhibition of ER or mitochondrial Ca^{2+} signaling (FCCP) had no effect on bpAP-evoked Ca^{2+} transients and failed to occlude the effects of GPN (n = 3–5 cells/condition). Significance was assessed with paired t tests (single paired comparisons) or Kruskal-Wallis and post hoc Dunn's tests (multiple comparisons). Error bars represent SEM. See also Figure S3.

bpAP-evoked Ca^{2+} signaling (n = 3 cells; paired t test, $p < 0.05$; Figures 3C and 3D). Collectively, these findings suggest that the ER is unlikely to play a role in bpAP-evoked lysosomal Ca^{2+} release.

We also examined the possibility that lysosomal Ca^{2+} release was coupled to mitochondrial Ca^{2+} stores. Consistent with a previous report (Markram et al., 1995), application of the mitochondrial uncoupler FCCP (1 μM, 5 min) had no effect on bpAP-evoked Ca^{2+} transients (n = 5 cells; Kruskal-Wallis test, $p > 0.05$; Figure 3D) and failed to occlude the effects of GPN (n = 5 cells; paired t test, $p < 0.05$; Figure 3D). These findings suggest that, under our experimental conditions, the lysosome is the only internal Ca^{2+} store that contributes to bpAP-evoked Ca^{2+} signaling and is functionally coupled to VGCCs.

Lysosomal Ca^{2+} Signaling Drives Fusion of the Lysosomes with the Plasma Membrane

What might be the function of lysosomal Ca^{2+} release in neurons? The lysosome itself is known to undergo Ca^{2+}-dependent fusion both with other organelles and with the plasma membrane (Luzio et al., 2007b). The source of Ca^{2+} in such instances can be the lysosome itself (Pryor et al., 2000; Luzio et al., 2007a, 2007b; Davis et al., 2012; Galione, 2015). Lysosomal fusion to the plasma membrane has been shown to release chemical messengers or enzymes in several cell types (Luzio et al., 2007b; Galione, 2015). If present in neurons, such a mechanism could have implications for activity-dependent processing in dendrites. Additionally, our finding of the strong functional coupling between VGCC activation and lysosomal Ca^{2+} release in neurons suggests that lysosomes would be ideally situated for activity-dependent fusion with the plasma membrane, akin to synaptic vesicles in the presynaptic terminal.

We investigated whether activity-dependent release of Ca^{2+} from the lysosome could trigger fusion of the lysosome to the plasma membrane in neuronal dendrites. We started by using total internal reflection fluorescence microscopy (TIRFM) to monitor the dynamics of LysoTracker-stained lysosomes near the plasma membrane in dissociated hippocampal cultures. We found weak LysoTracker labeling in the dendrites under baseline conditions (Figure 4A). However, when we elevated extracellular K^+ to 45 mM to drive strong dendritic depolarization and VGCC activity (Wu et al., 2001a, 2001b), we observed a marked increase in the intensity of labeled puncta (Figure 4B) and an overall increase in surface fluorescence (6.50%ΔF/F ± 0.05%ΔF/F; n = 5 cells; z test versus 0, $p < 0.01$; Figure 4A; Movie S1). Depolarization had no such effects on cells pre-incubated with NED-19, and, instead, only decreases in fluorescence were observed because of the effects of photobleaching (−1.62%ΔF/F ± 0.76%ΔF/F; n = 5 cells; NED-19 versus K^+, Mann-Whitney test, $p < 0.05$; Figures 4A and 4B).

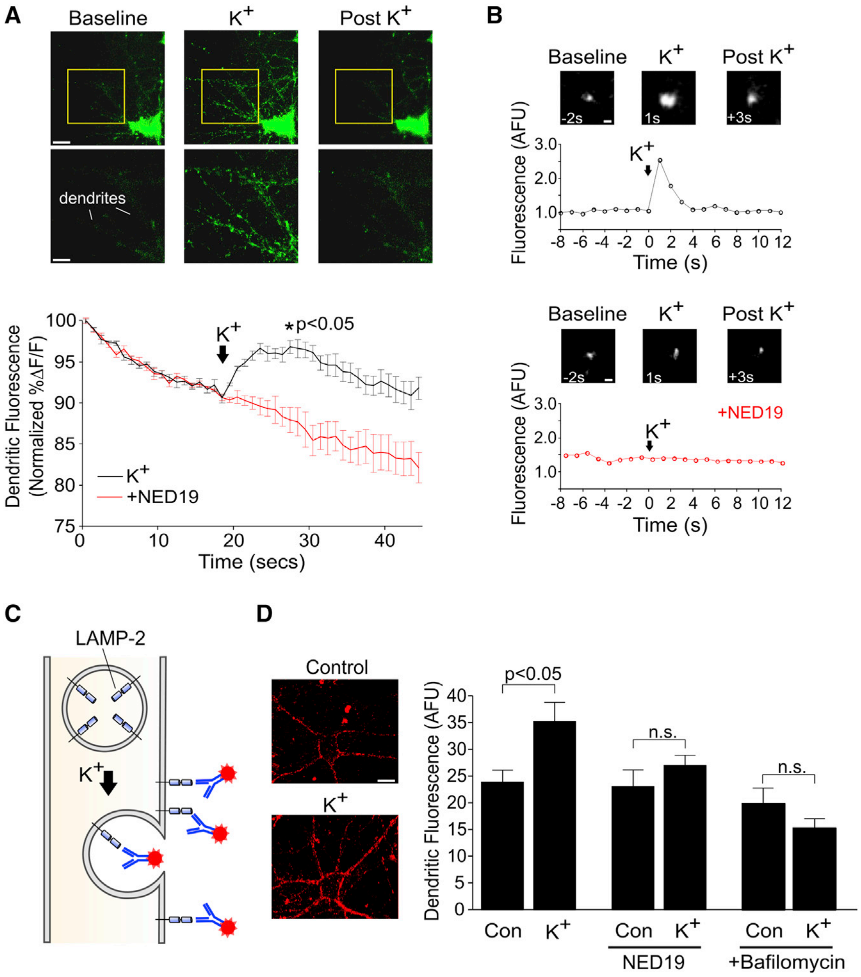

Figure 4. Lysosomal Ca^{2+} Signaling Drives Fusion of the Lysosomes with the Plasma Membrane

(A) Top: TIRFM images of a dissociated hippocampal neuron loaded with LysoTracker. K^+ stimulation resulted in translocation of LysoTracker-stained puncta to the cell surface (scale bar, 10 μm). The imaged area in the yellow box is magnified below (scale bar, 5 μm). Bottom: average change in LysoTracker fluorescence across time (n = 5 cells/condition). K^+-induced increases in fluorescence were abolished by NED-19. Time-dependent decreases in fluorescence reflected photobleaching. Significance was assessed with Mann-Whitney test at the peak of fluorescence.

(B) Sample TIRFM images of individual LysoTracker-stained puncta in (A) (scale bar, 2 μm). Graphs depict fluorescence intensity in AFU against time.

(C) Schematic of live-cell immunolabeling of LAMP-2. Fluorescently tagged antibodies targeting the lumenal domain of LAMP-2 were applied to neuronal cultures during K^+ stimulation. Fluorescent labeling would require fusion of the lysosome with the plasma membrane.

(D) Left: sample images depicting surface LAMP-2 antibody labeling in dissociated hippocampal cultures (scale bar, 10 μm). Labeling in K^+-treated cultures was greater than under control conditions. Right: the graph depicts group averages of LAMP-2 labeling (n = 10–23 cells/condition). NED-19 and bafilomycin prevented activity-dependent increases in LAMP-2 labeling.

Significance was assessed with Kruskal-Wallis and post hoc Dunn's tests. Error bars represent SEM. See also Figure S6 and Movie S1.

These findings suggest that membrane depolarization mobilized lysosomes close to the cell surface and in a manner dependent on lysosomal Ca^{2+} signaling; however, from these data alone, we could not determine whether such mobilization was followed by fusion of the lysosome with the plasma membrane.

We next used live-cell immunolabeling in dissociated neurons to examine whether lysosomes were capable of fusing with the plasma membrane in an activity-dependent manner. We applied a fluorescently labeled antibody targeting the lumenal domain of the lysosome-associated membrane protein LAMP-2 during K^+-mediated depolarization. Under these conditions, fluorescent labeling of LAMP-2 would require lysosomes to fuse with the plasma membrane (Figure 4C). Using this technique, we found that membrane depolarization resulted in significantly greater amounts of immunofluorescence in the dendrite compared with control conditions (in arbitrary fluorescence units [AFU]; K^+, 35 ± 4; control, 24 ± 2; n = 23 cells/condition; post hoc Dunn's test, $p < 0.05$; Figure 4D). Knocking out LAMP-2 using CRIPSR/Cas9 abolished all surface labeling, confirming that immunofluorescence was specifically associated with LAMP-2 labeling (Figure S6). Moreover, pre-incubation of cells with either NED-19 or bafilomycin abolished activity-induced increases in LAMP-2 fluorescence (n = 10 cells; NED-19 versus NED-19 + K^+, post hoc Dunn's test, p = 0.86;; n = 11 cells; bafilomycin versus bafilomycin + K^+, post hoc Dunn's test, p = 0.93; Figure 4D). These findings suggest that neuronal depolarization drives lysosomal fusion with the plasma membrane and in a manner dependent on activity-evoked lysosomal Ca^{2+} release.

To image lysosomal fusion with improved rapidity and sensitivity, we made use of superecliptic pHlourin (SEP), a pH-sensitive GFP that is commonly used as a probe for synaptic vesicle fusion. We tagged SEP to the lumenal domain of LAMP-2 (Figure 5A). The fluorescence of LAMP2-SEP, which would be otherwise quenched in the acidic environment of the lysosome, should be detected upon exposure to the pH neutral environment of the extracellular fluid following lysosomal fusion with the plasma membrane. We expressed LAMP2-SEP in dissociated cultures. LAMP2-SEP localized to lysosomes, retained its pH sensitivity, and exhibited maximal fluorescence upon de-acidification of the lysosome with NH_4Cl (Figure S7). Moreover, upon K^+ stimulation, we observed a large and rapid increase in LAMP2-SEP fluorescence throughout the dendritic arbor (68%ΔF/F ± 17%ΔF/F; n = 5 cells; versus baseline; Mann-Whitney test, $p < 0.05$; Figures 5B–5D). This was followed

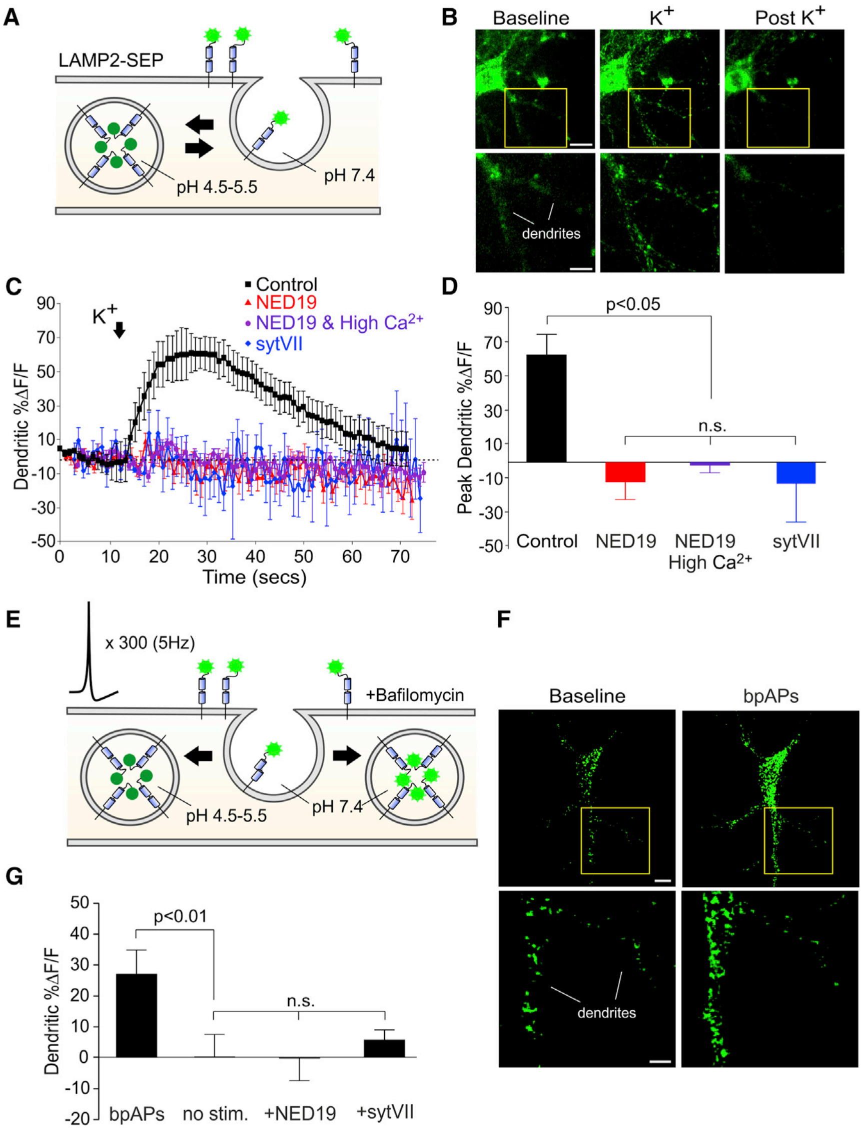

Figure 5. Imaging Lysosomal Fusion with LAMP2-SEP

(A) Schematic of LAMP2-SEP. pH-dependent changes in fluorescence were used to monitor lysosomal fusion.

(B) TIRFM image of a dissociated hippocampal neuron transfected with LAMP2-SEP (scale bar, 10 μm). The imaged area in the yellow box is magnified below (scale bar, 5 μm).

(C and D) Average K^+-evoked changes in LAMP2-SEP fluorescence in time (D, n = 5–7 cells/condition) with peak changes quantified in (D). Activity-dependent increases in fluorescence were abolished by NED-19 or intracellular loading of Syt7.

(E) Schematic of the experiment using the alkaline trap to examine lysosome fusion in response to bpAPs. Neurons were stimulated with 300 action potentials at 5 Hz in the presence of bafilomycin to prevent re-acidification of the lysosome upon internalization.

(F) Confocal image of a dissociated neuron transfected with LAMP2-SEP (scale bar, 10 μm). The imaged area in the yellow box is magnified below (scale bar, 5 μm). bpAPs triggered an increase in fluorescence.

(G) Average bpAP-evoked change in LAMP2-SEP fluorescence (n = 6–7 cells/condition). No activity-dependent increases in fluorescence occurred following extracellular application of NED-19 or intracellular loading of Syt7.

Significance was assessed with Kruskal-Wallis and post hoc Dunn's tests. Error bars represent SEM. See also Figure S7 and Movie S2.

by a return to basal fluorescence within 65 s (n = 5 cells; fluorescence at 65 s versus baseline; Mann-Whitney test, $p = 0.85$; Figures 5B and 5C; Movie S2), likely reflecting the surface retrieval and re-acidification of LAMP-2. Fusion of the lysosome with the plasma membrane in several cell types has previously been shown to require the actions of synaptotagmin 7 (Syt7) and can be blocked by loading cells with the cytosolic domain of Syt7 (Martinez et al., 2000; Luzio et al., 2007b). Therefore, to confirm that increases in LAMP2-SEP fluorescence reflected bona fide fusion of the lysosome with the plasma membrane, we loaded cells with the cytosolic domain of recombinant Syt7 (100 μg/mL). Under these conditions, we detected no increases in fluorescence following K^+ treatment (n = 5 cells; Syt7 versus control, post hoc Dunn's test, $p < 0.05$; Figures 5C and 5D). Increases in fluorescence were also abolished by NED-19 (n = 7 cells; versus control, post hoc Dunn's test, $p < 0.05$; Figures 5C and 5D), even when Ca^{2+} influx from VGCCs was increased by elevating extracellular Ca^{2+} from 2 mM to 4 mM and by adding the voltage-gated K^+ channel blocker 4-AP (100 μM) (n = 7 cells; NED-19 with high Ca^{2+} versus control, post hoc Dunn's test, $p < 0.05$; Figures 5C and 5D). These findings confirm that LAMP2-SEP can detect fusion of lysosomes with the plasma membrane and that Ca^{2+} release from lysosomal stores is integral for the fusion of the lysosome with the plasma membrane even when Ca^{2+} influx from VGCCs is augmented.

LAMP2-SEP gave us the added advantage of examining whether lysosome fusion could be triggered by dendritic depolarization evoked by bpAPs. Because we could not reliably detect increases in LAMP2-SEP fluorescence to a single bpAP, we made use of the alkaline trap method (Grubb and Burrone, 2009), which blocks vesicle re-acidification with the V-ATPase inhibitor bafilomycin. This would enable fusion-mediated increases in LAMP2-SEP fluorescence, if present, to accumulate to measurable levels during repeated stimulation despite LAMP-2 surface retrieval (Figure 5E). It is important to mention that, although chronic incubation (>60 min) of bafilomycin inhibited lysosomal Ca^{2+} signaling (Figure 2B), acute applications had no effect, likely because of the poor membrane permeability of the drug. In keeping with this, brief application of bafilomycin

(<10 min) in the absence of any electrical stimulation produced no change in LAMP2-SEP fluorescence (n = 6 cells; z test versus 0, p = 0.93; Figure 5G), suggesting that the drug alone had no effect on lysosomal pH. However, we did observe robust increases in fluorescence when bafilomycin application was followed by low-frequency (5-Hz) stimulation of 300 bpAPs, which was achieved using field electrodes (27%ΔF/F ± 6%ΔF/F; n = 6 cells; versus no stimulation; post hoc Dunn's test, p < 0.01; Figures 5F and 5G). No such increases were observed when stimulation was delivered in the presence of NED-19 (n = 7 cells; versus no stimulation; post hoc Dunn's test, p = 0.99; Figure 5G) or following intracellular loading of Syt7 (n = 5 cells; versus no stimulation; post hoc Dunn's test, p = 0.44; Figure 5G). Our findings suggest that bpAP-evoked lysosomal Ca^{2+} release triggers lysosomal fusion with the plasma membrane.

Lysosomal Fusion Results in the Release of Cathepsin B

The fusion of the lysosome with the plasma membrane likely results in the release of its contents into the extracellular space. We hypothesized that the release of lysosomal proteases may be required for the activity-dependent restructuring of the extracellular matrix (ECM). This would be of potential relevance for long-term synaptic changes, which are known to require ECM remodelling (Wlodarczyk et al., 2011; Dziembowska and Wlodarczyk, 2012). In this regard, Cathepsin B was of particular interest to us. The cathepsins are a family of lysosomal proteases that generally show preferential activation in acidic environments. Cathepsin B, however, exhibits enzymatic activity in both acidic and pH-neutral environments, suggesting that, upon lysosomal fusion, Cathepsin B would likely retain some form of enzymatic activity in the pH-neutral extracellular space (Mort et al., 1984; Linebaugh et al., 1999).

We first examined whether Cathepsin B could, in fact, be released by neuronal lysosomes in an activity-dependent manner. We incubated dissociated hippocampal neurons with Magic Red Cathepsin B fluorogenic substrate, which fluoresces upon Cathepsin B-mediated cleavage. Application of the substrate resulted in labeling of fluorescent puncta that co-localized with LysoTracker, confirming that active Cathepsin B was ubiquitously present in neuronal lysosomes (Figure 6A; Figure S8). Magic Red fluorescence also co-localized with Cathepsin B-GFP fluorescence, confirming that it was labeling Cathepsin-B containing compartments (Figure S8). We then stimulated cultures with 45 mM K^+ and found that this resulted in a loss of Magic Red fluorescence in some of the imaged puncta, consistent with the notion of lysosomal exocytosis, and the loss of Cathepsin B activity from these sites (Figure 6A). A similar loss of fluorescence was seen with LysoTracker (Figure 6A; Figure S8). The average loss of Magic Red fluorescence across puncta (−15.6%ΔF/F ± 1.6%ΔF/F; n = 226 puncta from 6 cells; Figure 6B) was significantly greater than in unstimulated cultures (n = 254 puncta from 5 cells; post hoc Dunn's test, p < 0.01; Figure 6B), and in cultures pre-treated with NED-19 (n = 152 puncta from 5 cells; post hoc Dunn's test, p < 0.01; Figure 6B). We defined puncta undergoing fusion as those exhibiting a loss of Magic Red fluorescence of >3 SD from the average fluorescent change measured in unstimulated control experiments. Using this metric, we found that, with K^+ stimulation, the fraction of Cathepsin B puncta undergoing fusion (8.4% ± 1.8%; n = 226 puncta from 6 cells; Figure 6C) was significantly greater than that found in unstimulated controls (n = 254 puncta from 5 cells; corrected z test, p < 0.01; Figure 6C) or in cultures pre-treated with NED-19 (n = 152 puncta from 5 cells; versus K^+ stimulation; corrected z test, p < 0.01; Figure 6C). Live-cell immunolabeling of synapses with anti-GluA1 antibodies additionally revealed that >95% of Cathepsin B puncta, both the total pool of puncta and those undergoing activity-dependent fusion, were located within 1 μm of a GluA1-labeled punctum (Figure S8), suggesting that Cathepsin B release occurs in the vicinity of synapses.

Cathepsin B Release Activates MMP-9

ECM remodelling is canonically associated with matrix metalloproteinases (MMPs). One means by which Cathepsin B can promote ECM remodelling is by recruiting MMP activity. It does so by cleaving TIMP-1, an endogenous and potent inhibitor of MMP signaling (Kostoulas et al., 1999; Murphy and Lynch, 2012). We therefore examined whether activity-dependent release of lysosomal Cathepsin B could contribute to ECM remodeling in cultured hippocampal slices. Using an ELISA, we first confirmed whether we could detect activity-dependent elevations of Cathepsin B in the extracellular fluid. Similar to our findings in dissociated neurons, we found that K^+ treatment elevated extracellular Cathepsin B in hippocampal slices (in a.u.; control,0.19 ± 0.011; n = 8 slices; K^+, 0.31 ± 0.006; n = 5 slices; post hoc Bonferroni test, p < 0.01; Figure 6D). Pre-treatment with NED-19 greatly reduced this activity-dependent increase (n = 7 slices; NED-19 + K^+ versus K^+; post hoc Bonferroni test, p < 0.01; Figure 6D), although it did not quite abolish it (n = 7 and 5 slices; NED-19 versus NED-19 + K^+; post hoc Bonferroni test, p < 0.05, Figure 6D). We next examined whether extracellular Cathepsin B was enzymatically active. To do so, we incubated slices with a Cathepsin B fluorogenic substrate (Z-Arg-Arg-AMC) and monitored increases in extracellular fluorescence as a readout of enzymatic activity (Figure 6E). Cathepsin B activity was minimal under control conditions (77 ± 502 AFU; n = 5 slices; z test versus 0, p = 0.87; Figure 6E), greatly enhanced after K^+ stimulation (1,717 ± 850 AFU; n = 5 slices; K^+ versus control; post hoc Bonferroni test, p < 0.05; Figure 6E), and abolished by NED-19 (n = 5 slices/condition; NED-19 versus NED-19 + K^+; post hoc Bonferroni test, p = 0.99; Figure 6E). Collectively, these findings are consistent with the activity-dependent release of active Cathepsin B from lysosomal stores.

Cathepsin B is able to activate MMP signaling by cleaving the MMP inhibitor TIMP-1 (Kostoulas et al., 1999; Murphy and Lynch, 2012). We therefore examined whether activity-dependent release of lysosomal Cathepsin B could mediate an increase in extracellular MMP activity in hippocampal tissue. To examine this, we incubated hippocampal slices with a broadly selective MMP fluorogenic substrate (PEPDAB0502), which fluoresces upon MMP-mediated cleavage (Miller et al., 2011). We examined MMP-associated fluorescence either in the presence or absence of the membrane-impermeable Cathepsin B inhibitor CA-074 (1 μM), which we used to selectively inhibit extracellular Cathepsin B activity (Linebaugh et al., 1999; Mitrović et al., 2016). Notably, we saw no effect of CA-074 on MMP activity under basal conditions (in AFU; control, 2,083 ± 207; n = 29 slices;

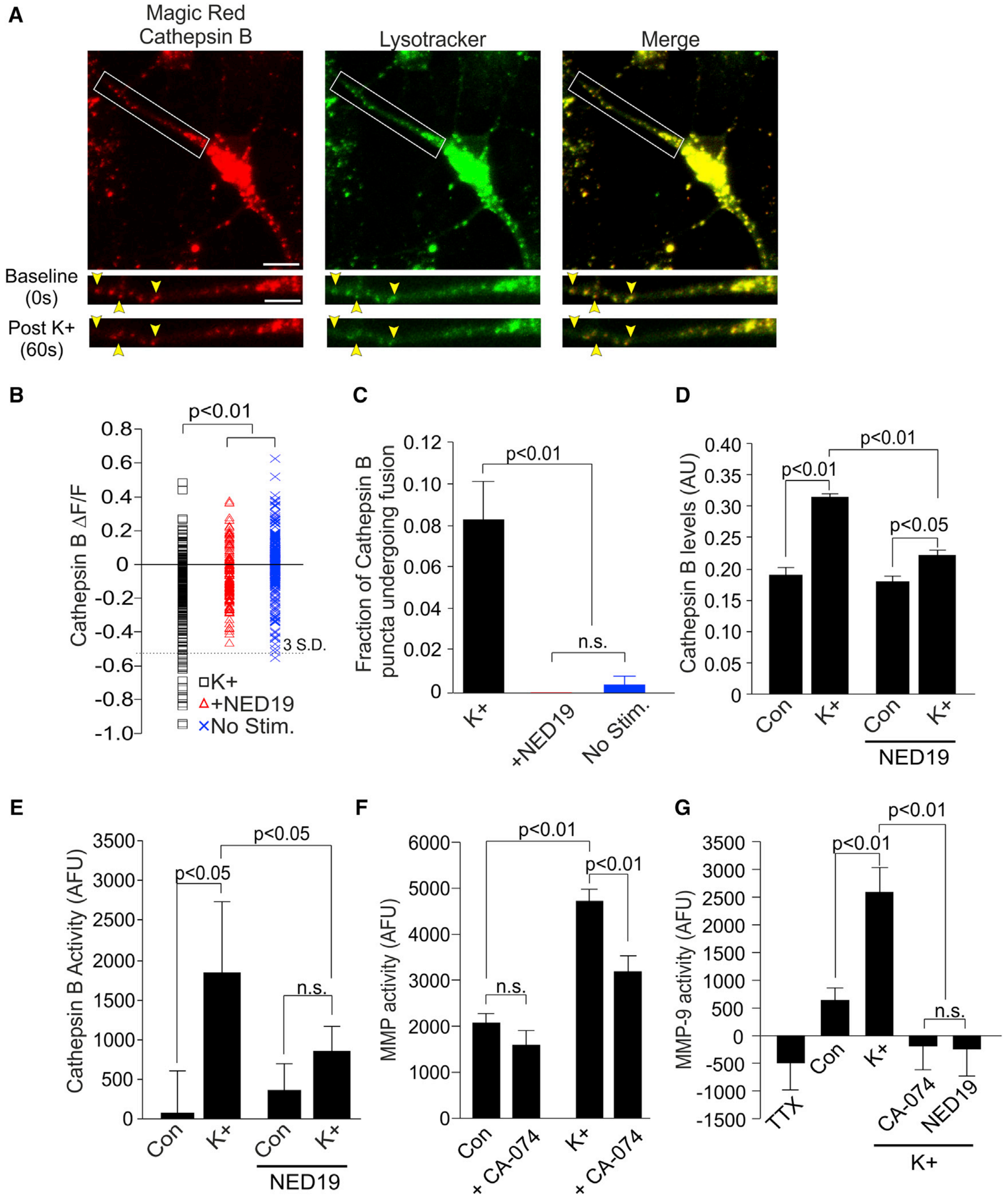

Figure 6. Lysosomal Fusion Results in the Release of Cathepsin B, which Activates MMP-9 Signaling

(A) Confocal image of a dissociated hippocampal neuron (scale bar, 10 μm) loaded with Magic Red Cathepsin B fluorogenic substrate (red) and LysoTracker (green). The dendrite in the white box is magnified below (scale bar, 5 μm) Cathepsin B-associated fluorescence co-localized with LysoTracker-labeled puncta (yellow). Application of 45 mM K^+ resulted in de-staining of some of the imaged puncta (yellow arrowheads), consistent with lysosomal fusion and loss of Cathepsin B activity.

(B) Activity-dependent change in Cathepsin B-associated fluorescence of imaged puncta across experiments (n = 152–254 puncta from 5–6 cells/condition). The dashed line marks 3 SD from average fluorescent changes recorded in unstimulated controls (no stim.). Significance was assessed with Kruskal-Wallis and post hoc Dunn's tests.

(C) Fraction of Cathepsin B puncta undergoing fusion, as defined by a fluorescence loss of >3 SD (dashed line in B). NED-19 prevented activity-dependent loss of Cathepsin B activity. Significance was assessed with z tests (Bonferroni correction).

(D) Extracellular Cathepsin B expression levels in hippocampal slices measured using ELISA (n = 5–7 slices/condition). Activity was stimulated by elevating extracellular K^+ to 30 mM. Activity-dependent increases in Cathepsin B secretion were abolished with NED-19.

(legend continued on next page)

CA-074, 1,601 ± 317; n = 15 slices; post hoc Bonferroni test, $p = 0.40$; Figure 6F). However, given our finding that Cathepsin B release is activity-dependent, we repeated the experiment under high K^+ conditions to promote Cathepsin B secretion from lysosomal stores. Under these conditions we found that MMP activity was significantly greater than that of controls (AFU = 4,725 ± 259; n = 13 slices; K^+ versus control; post hoc Bonferroni test, $p < 0.01$; Figure 6F) and now sensitive to Cathepsin B inhibition by CA-074 (AFU = 3,187 ± 317; n = 12 slices; CA-074 versus K^+; post hoc Bonferroni test, $p < 0.01$; Figure 6F). These findings are consistent with the idea that activity-dependent release of Cathepsin B stimulates MMP activity in hippocampal slices.

Several studies have demonstrated that MMP-9, in particular, is necessary for the maintenance of long-term potentiation (LTP) and dendritic spine growth (Wang et al., 2008; Wlodarczyk et al., 2011; Dziembowska and Wlodarczyk, 2012). We were therefore interested in examining how Cathepsin B affected MMP-9 activity in hippocampal slices. For this purpose, we again incubated slices with the MMP fluorogenic substrate but did so either in the presence or absence of an MMP-9 inhibitor (100 nM MMP-9 inhibitor I). We took the reduction in MMP-associated fluorescence that occurred in the presence of the inhibitor as a readout of the levels of MMP-9 activity present under a particular condition. Under control conditions, we detected a small but significant level of MMP-9 activity (AFU = 635 ± 220; n = 14 slices; z test, $p < 0.01$; Figure 6G). Given that MMP-9 activation is driven by neuronal activity and not normally detectable under baseline conditions (Nagy et al., 2006; Dziembowska et al., 2012), we reasoned that the MMP-9 activity in our experiment was likely driven by spontaneous neuronal activity in our slices. Indeed, we found that MMP-9 activity was abolished in the presence of TTX (n = 9 slices; z test versus 0, $p > 0.05$; Figure 6G), although this did not reach significance compared with control levels of activity (post hoc Bonferroni test, $p = 0.21$). Conversely, elevating neuronal activity by K^+ stimulation greatly enhanced MMP-9 activity from control levels (AFU = 2,592 ± 439; n = 10 slices; K^+ versus control; post hoc Bonferroni test, $p < 0.01$; Figure 6G). Given that K^+ stimulation also enhances Cathepsin B release, we asked whether activity-dependent release of Cathepsin B was necessary for the activity-dependent increase in MMP-9 activity. We found that addition of either CA-074, to inhibit extracellular Cathepsin B, or NED-19, to prevent activity-dependent Cathepsin B release, abolished MMP-9 activity in K^+ stimulated slices (CA-074, n = 11 slices; versus K^+; post hoc Bonferroni test, $p < 0.01$; NED-19, n = 5 slices; post hoc Bonferroni test, versus K^+, $p < 0.01$; Figure 6G). Therefore, activity-dependent increase of MMP-9 activity in hippocampal slices requires activity-dependent release of Cathepsin B.

MMP-9 and its endogenous inhibitor TIMP-1 co-localize to secretory vesicles and are co-released with neuronal activity (Nagy et al., 2006; Sbai et al., 2008; Dziembowska et al., 2012). MMP-9 activity, therefore, will not necessarily depend on the absolute levels of MMP-9 in tissue but on the ratio of MMP-9 to TIMP-1. Therefore, to provide additional evidence that Cathepsin B is capable of recruiting MMP-9 activity, we examined the MMP-9/TIMP-1 ratio in hippocampal slices. We reasoned that this ratio should be increased by neuronal activity and in a manner dependent on lysosomal Cathepsin B. In agreement with this, western blot analysis showed a marked increase in the MMP-9/TIMP-1 ratio in slices treated with K^+ (Figure S9). Inhibiting Cathepsin B release with NED-19 or Cathepsin B activity with CA-074 inhibited these increases (Figure S9).

Cathepsin B Release Is Necessary for MMP-9-Mediated Long-Lasting Structural Plasticity

Long-lasting changes in synaptic function are thought to ultimately require stable, structural changes in the pre- or post-synaptic elements, with LTP induction being associated with long-lasting expansion of the postsynaptic density and a stable enlargement of the dendritic spine head. Such changes require ECM remodelling by MMP-9 (Wang et al., 2008; Wlodarczyk et al., 2011; Dziembowska and Wlodarczyk, 2012). Given that lysosomal-mediated release of Cathepsin B provides a means by which neuronal activity can trigger activation of MMP-9, we hypothesized that the lysosome would play a pivotal role in the maintenance of structural plasticity. We therefore examined the effect that inhibiting lysosomal Ca^{2+} signaling and fusion with NED-19 would have on structural enlargements of dendritic spines induced by Hebbian pairing. For this purpose, we filled CA1 neurons with Alexa Fluor 488 dye to visualize spine structure and induced LTP by pairing brief (1 ms) photolysis of caged glutamate on a target spine with 3–5 bpAPs (Figure 7A). The pairing was repeated 60 times at 5 Hz. This protocol produced a rapid and long-lasting expansion of the target spine that was present at 1 min following stimulation (foldΔ = 1.75 ± 0.12; n = 9 cells; z test versus 1.00, $p < 0.01$; Figures 7B and 7E) and remained stable for at least 60 min thereafter (n = 9 cells; foldΔ at 60 min versus 1 min; Mann-Whitney, $p = 0.61$; Figure 7B). No enlargements were observed at neighboring spines (n = 9 cells; z test versus 1.00, $p = 0.37$; Figures 7B and 7E). Remarkably, NED-19 abolished long-lasting changes in structural growth (n = 6 cells; foldΔ at 60 min; z test versus 1.00, $p = 0.78$; Figures 7B and 7E) despite having no effect on the initial spine growth recorded 1 min after paired stimulation (n = 6 cells; versus control; Kruskal-Wallis, $p = 0.29$; Figures 7B and 7E); similar results were obtained when lysosomal Ca^{2+} release was instead inhibited with desensitizing concentrations of NAADP in the recording electrode (n = 6 cells; foldΔ at 1 min versus

(E) Extracellular Cathepsin B activity levels measured in hippocampal slices using the fluorogenic Cathepsin B substrate Z-Arg-Arg-AMC (n = 5 slices/condition). Activity-dependent increases in Cathepsin B activity were abolished by NED-19.

(F) Average MMP activity in hippocampal slices measured using an MMP fluorogenic substrate (n = 12–29 slices/condition). Cathepsin B inhibition with CA-074 reduced MMP signaling in an activity-dependent manner.

(G) Average MMP-9 fluorogenic activity in hippocampal slices (n = 5–14 slices/condition; Experimental Procedures). MMP-9 activity was driven by K^+ stimulation and abolished by NED-19 and CA-074. Significance was assessed with ANOVA and post hoc Bonferroni tests.

Error bars represent SEM. See also Figures S8 and S9.

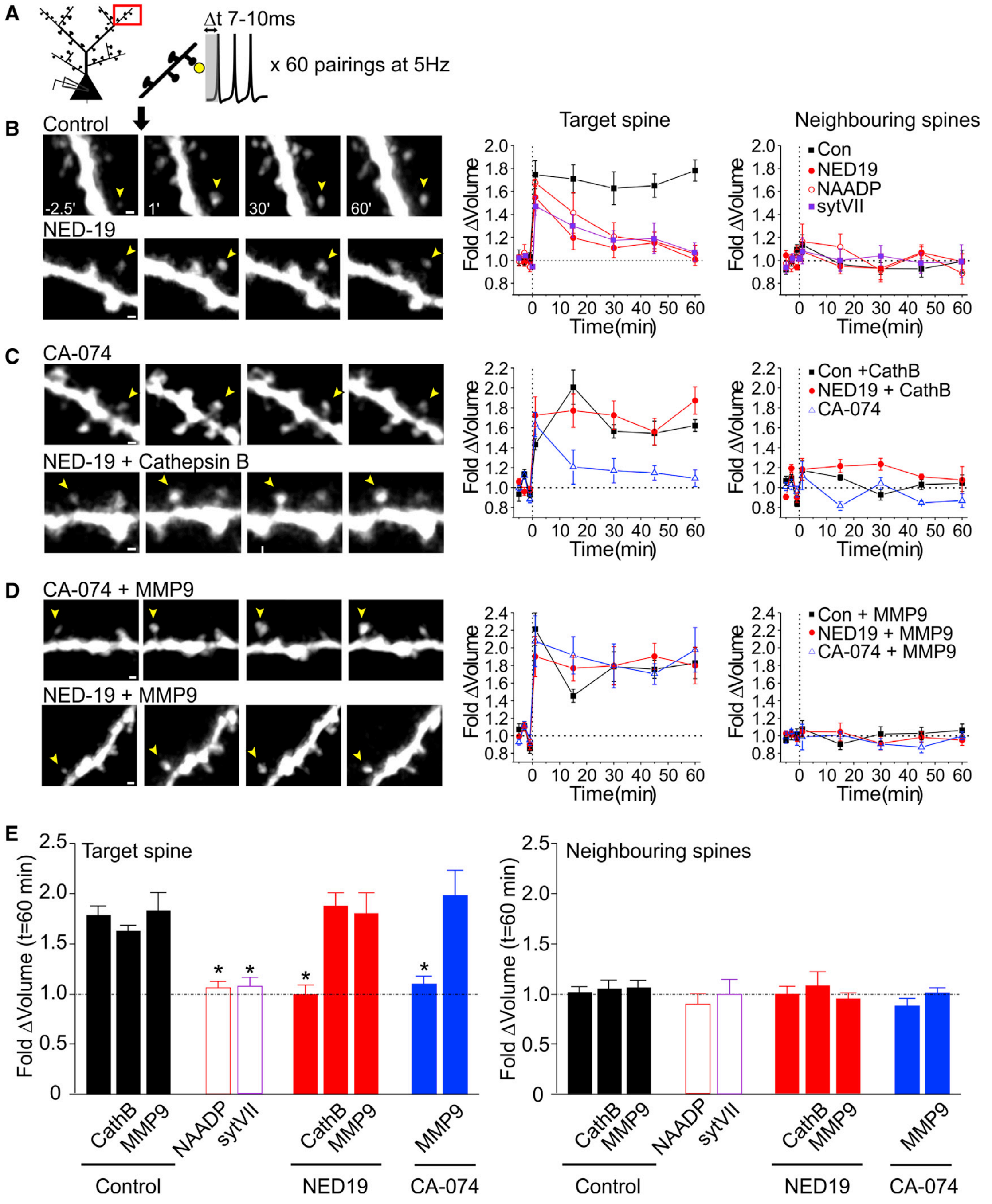

Figure 7. Cathepsin B Release Is Necessary for MMP-9-Mediated Long-Lasting Structural Plasticity

(A) Schematic of the experiment. Glutamate photolysis at a target spine was paired with bpAPs to induce long-lasting structural plasticity.

(B–D) Left: sample images of CA1 dendritic spines (scale bars, 1 μm). Images of target (yellow arrow) and neighboring spines are shown at −2.5, +1, +30, and +60 min following paired stimulation. Right: average change in spine volume of target and neighboring spines across time (n = 5–9 cells/condition). Long-lasting structural plasticity was abolished by inhibiting lysosomal function with NED-19, desensitizing concentrations of NAADP, or intracellular loading of Syt7 (B). Structural plasticity was also abolished by inhibiting Cathepsin B activity with CA-074 (C). Bath application of either active Cathepsin B (C) or active MMP-9 (D) for 10 min after the start of paired stimulation rescued long-lasting structural plasticity. Neighboring spines remained unchanged across experimental conditions.

(E) Average change in spine volume at 60 min post-pairing for target and neighboring spines (n = 5–9 cells/condition).

Significance was assessed with Kruskal-Wallis and post hoc Dunn's tests. Asterisks denote the significant differences ($p < 0.05$) from control conditions. All other comparisons with control groups are not significant. Error bars represent SEM.

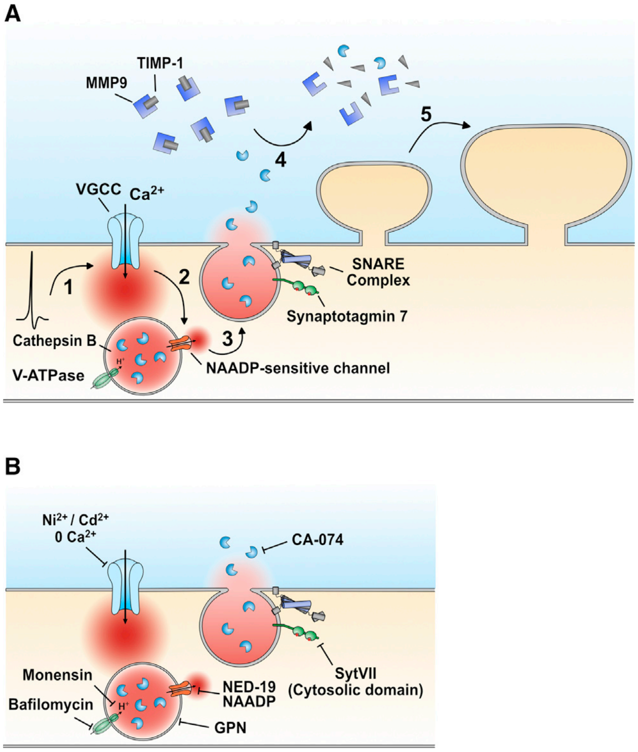

Figure 8. Proposed Model

(A) 1: bpAPs activate dendritic VGCCs. 2: VGCC-mediated Ca^{2+} influx triggers Ca^{2+} release from the lysosome via an NAADP-sensitive channel. 3: lysosomal Ca^{2+} release triggers fusion of the lysosome with the plasma membrane, resulting in the release of Cathepsin B. 4: Cathepsin B cleaves TIMP-1, releasing MMP-9 from inhibition. 5: MMP-9 activity maintains the long-lasting structural plasticity of dendritic spines.

(B) Targets of pharmacological reagents used in this study.

control; Kruskal-Wallis, p = 0.29; foldΔ at 60 min, z test versus 1.00, p = 0.46; Figures 7B and 7E) or when lysosomal fusion was inhibited with intracellular loading of the cytosolic domain of Syt7 (n = 5 cells; foldΔ at 1 min versus control; Kruskal-Wallis, p = 0.29; foldΔ at 60 min; z test versus 1.00, p = 0.46; Figures 7B and 7E). These findings suggest that lysosomal Ca^{2+} release and fusion are specifically involved in the maintenance, as opposed to the induction, of structural plasticity; a very similar role has been reported for MMP-9 (Wang et al., 2008). We hypothesized that the reason why pharmacological impairment of lysosomal function prevented structural plasticity was a failure of the postsynaptic neuron to release Cathepsin B during neuronal activity. Consistent with this idea, we found that inhibition of extracellular Cathepsin B with CA-074, was able to phenocopy the effects of NED-19 (n = 5 cells; foldΔ at 1 min; CA-074 versus NED-19; post hoc Dunn's test, p = 0.66; foldΔ at 60 min; CA-074 versus NED-19; post hoc Dunn's test, p = 0.99; Figures 7C and 7E), whereas exogenous application of active Cathepsin B (5 μg/mL applied for 10 min after the start of pairing) rescued the NED-19 phenotype, restoring persistent expansion of the target spine to control levels (foldΔ at 60 min = 1.87 ± 0.14; n = 5 cells; versus control; post hoc Dunn's test, p = 0.99; Figures 7C and 7E) without affecting neighboring spines (n = 5 cells; Kruskal-Wallis test, p = 0.72; Figures 7C and 7E). In contrast, addition of active Cathepsin B in control experiments did not further augment spine expansion (n = 5 cells; versus control; post hoc Dunn's test, p = 0.99; Figures 7C and 7E), suggesting that Cathepsin B activity was not a limiting factor under normal conditions of plasticity induction. To then test whether the function of lysosome fusion and Cathepsin B release, in the context of structural plasticity, was to ultimately activate MMP-9, we attempted to rescue the plasticity deficits induced by CA-074 and NED-19 by exogenous application of active MMP-9 (5 μg/mL applied for 10 min after the start of pairing). We found that MMP-9 did indeed restore persistent expansion of the target spine to control levels in both NED-19- and CA-074-treated slices (NED-19 + MMP-9, n = 5 cells; foldΔ at 60 min versus control; post hoc Dunn's test, p = 0.99; CA-074 + MMP-9, n = 5 cells; foldΔ at 60 min; versus control; post hoc Dunn's test, p = 0.99; Figures 7D and 7E) without affecting neighboring spines (NED-19 + MMP-9, n = 5 cells; Kruskal-Wallis test, p = 0.72; CA-074 + MMP-9, n = 5 cells; Kruskal-Wallis test, p = 0.72; Figures 7D and 7E). Notably, addition of active MMP-9 in control experiments did not further augment spine expansion (n = 5 cells; foldΔ at 60 min versus control; post hoc Dunn's test, p = 0.99; Figures 7D and 7E), suggesting that the activity of MMP-9, like that of Cathepsin B, was not a limiting factor under normal conditions of plasticity induction.

DISCUSSION

Here we found that dendritic Ca^{2+} influx from VGCCs during bpAPs triggered Ca^{2+} release from the lysosome. This Ca^{2+} release led to the fusion of the lysosome with the plasma membrane, resulting in the release of Cathepsin B and the subsequent recruitment of MMP-9 activity. We found that activity-dependent enhancement of MMP-9 signaling by Cathepsin B was necessary for the maintenance of the long-lasting dendritic spine growth that accompanies Hebbian pairing of synaptic activity with postsynaptic bpAPs (Figure 8A).

Our finding that bpAPs can trigger Ca^{2+} release from the lysosome is unexpected because it has long been thought that bpAP-evoked Ca^{2+} influx is mediated exclusively by VGCCs with little or no contribution from intracellular stores. The evidence for this comes from the fact that bpAP-evoked Ca^{2+} signals are essentially abolished following VGCC inhibition or removal of extracellular Ca^{2+} (Jaffe et al., 1992; Christie et al., 1995; Spruston et al., 1995; Helmchen et al., 1996), whereas inhibition of ER or mitochondrial Ca^{2+} signaling is without effect (Markram et al., 1995; Emptage et al., 1999; Kovalchuk et al., 2000). We found similar effects in our own study when blocking VGCC-, ER-, and mitochondrion-mediated Ca^{2+} signaling; however, we were also able to unmask a role for lysosomal stores. Notably, we found no evidence of lysosomal Ca^{2+} release under conditions in which VGCC-mediated Ca^{2+} influx was inhibited, suggesting that lysosomal Ca^{2+} release was likely triggered by Ca^{2+} influx through VGCCs. Others have demonstrated that Ca^{2+} from other sources, such as the ER, can trigger lysosomal Ca^{2+} release (Patel et al., 2001; Morgan et al., 2013), potentially by directly activating TPCs (Pitt et al., 2010, 2014); although the exact mechanism remains unclear, it does suggest that TPCs are

regulated both by Ca^{2+} and by NAADP. To the best of our knowledge, we are the first to report evidence of a functional coupling between VGCCs and lysosomes, which may potentially be a feature unique to excitable tissue. Certainly, such a coupling is an efficient means of endowing lysosomes with voltage sensitivity, thereby enabling lysosomal Ca^{2+} release to be driven by neuronal activity.

Although Ca^{2+} release from the lysosomes may serve a number of functions, in this study, we found that it drove the fusion of lysosomes with the plasma membrane. Why Ca^{2+} release from the lysosome, as opposed to other sources, is particularly important for its fusion with the plasma membrane is not clear, but a similar mechanism is thought to underlie lysosomal exocytosis in other cell types (Davis et al., 2012; Galione, 2015) and suggests that the lysosomal fusion machinery is sensitive to nanodomain Ca^{2+} signaling generated locally by the organelle itself. It is, however, important to note that, from out data alone, we cannot determine whether the lysosomes releasing Ca^{2+} in response to bpAPs are those that subsequently undergo fusion with the plasma membrane; it is possible that distinct subpopulations of lysosomes may differentially mediate these functions.

We found that fusion of the lysosome was accompanied by the release of Cathepsin B into the extracellular space. Cathepsin B release has generally been reported in the context of pathological conditions. In neural tissue, excessive levels of extracellular Cathepsin B release have been associated with cell death in traumatic brain injury, brain aneurysm, and several neurologic diseases such as aminotrophic lateral sclerosis (ALS), Alzheimer's disease, Parkinson's disease, and Huntington's disease (Pišlar and Kos, 2014; Hook et al., 2015). Potential mediators of Cathepsin B release under these conditions include reactive astrocytes, microglia, and/or macrophages (Ryan et al., 1995; Linebaugh et al., 1999; Kingham and Pocock, 2001; Gan et al., 2004; Murphy and Lynch, 2012). Although the exact mechanism by which these cells release Cathepsin B is not clear, if it involves lysosomal fusion with the plasma membrane, as reported in this study, then pharmacological inhibition of lysosomal Ca^{2+} signaling may provide a novel means for preventing excessive Cathepsin B release under pathological conditions.

We showed that Cathepsin B release was essential for the maintenance of long-term structural plasticity, likely by recruiting MMP-9 activity via cleavage of TIMP-1 (Kostoulas et al., 1999; Murphy and Lynch, 2012). Although several studies have demonstrated a role for MMP-9 in long-term synaptic plasticity, the mechanism by which neuronal activity triggers an increase in MMP-9 activity is not completely understood (Wlodarczyk et al., 2011; Dziembowska and Wlodarczyk, 2012). In the context of LTP, it is known that neuronal stimulation triggers the transport and translation of MMP-9 mRNA in neuronal dendrites, followed by MMP-9 release into the ECM (Nagy et al., 2006; Dziembowska et al., 2012), where it is converted into its active form, likely via plasmin-mediated cleavage of the MMP-9 pro-domain (Wlodarczyk et al., 2011). However, upon secretion, MMP-9 is complexed with its inhibitor TIMP-1 (Sbai et al., 2008), which is capable of inhibiting both active and inactive forms of MMP-9 (Ogata et al., 1995). Inhibition of TIMP-1 signaling, therefore, is likely to be crucial for the maintenance of LTP. Based on our results, we would suggest that the activity-dependent secretion of Cathepsin B from neuronal lysosomes is one possible means by which TIMP-1 signaling can be inhibited (Figure 8).

Although traditionally viewed as the degradative compartment of the cell, a growing body of evidence suggests that the lysosome functions as a Ca^{2+} store that is capable of mediating a diverse array of physiological functions across numerous cell types (Galione et al., 2010, 2015). In this study, we examined the role of lysosomes in neurons. Our findings reveal that lysosomes mediate activity-dependent Ca^{2+} signaling in the dendrites and, via the release of Cathepsin B, play an important and unexpected role in the long-term maintenance of structural plasticity.

EXPERIMENTAL PROCEDURES

Hippocampal Tissue Preparation

All animal work was carried out in accordance with the Animals (Scientific Procedures) Act, 1986 (UK) and under project and personal licenses approved by the Home Office (UK). Acute coronal hippocampal slices (350–400 μm) were prepared from male Wistar rats (post-natal day 14 [P14]–P21) and perfused (3 mL/min) with ACSF (120 mM NaCl, 2.5 mM KCl, 2 mM $CaCl_2$, 1 mM $MgCl_2$, 1.2 mM NaH_2PO_4, 26 mM $NaHCO_3$, 11 mM glucose, 0.2 mM ascorbic acid, and 1 mM Trolox; pH = 7.2–7.4; 32°C–34°C; 95% O_2 and 5% CO_2). Cultured hippocampal slices (350 μm) were prepared from male Wistar rats (P7). Slices (days in vitro [DIV] 10–15) were perfused (1–2 mL/min) with heated ACSF (32°C–34°C). For dissociated cultures, hippocampal cells from P1 male Wistar rats were plated onto 18-mm coverslips (3–6 × 10^4 cells/coverslip), pre-coated with poly-D-lysine (0.1 mg/mL) and fibronectin (0.05 mg/mL), and imaged on DIV 10–21 in Tyrode's buffer (128 mM NaCl, 5 mM KCl, 1 mM $MgCl_2$, 2 mM $CaCl_2$, 4.2 mM $NaHCO_3$, 20 mM glucose, and 15 mM HEPES; pH = 7.2–7.4; 22°C–24°C). For K^+ stimulation, 30 or 45 mM NaCl was replaced with KCl. See Supplemental Experimental Procedures for further details.

Ca^{2+} Imaging and Electrophysiology

CA3 or CA1 cells in hippocampal slices were recorded using either sharp microelectrodes (70–120 MΩ) or high-resistance patch electrodes (16–25 MΩ) to minimize intracellular dialysis. For Ca^{2+} imaging, sharp microelectrodes contained 0.5–1 mM OGB-1 (Thermo Fisher Scientific) dissolved in 200–400 mM potassium acetate. Patch electrodes contained OGB-1 (0.1–0.2 mM) dissolved in internal solution (135 mM KGluconate, 10 mM KCl, 10 mM HEPES, 2 mM $MgCl_2$, 2 mM Na_2ATP, and 0.4 mM Na_3GTP; pH = 7.2–7.4). Apical dendrites were imaged using confocal laser-scanning microscopy and a 488-nm argon laser. bpAPs were triggered with somatic current injection (1–2 nA, 5–10 ms) and recorded with an AxocLAMP-2B amplifier (Axon Instruments). See Supplemental Experimental Procedures for further details.

Cathepsin B and MMP Assays

Assays were done on slice culture supernatant using a plate reader. Cathepsin B levels were assessed using a colorimetric human Cathepsin B ELISA kit (ab119684, Abcam)according to the manufacturer's instructions. Cathepsin B and MMP activity was assessed by incubating slices either with the Cathepsin B fluorogenic substrate Z-Arg-Arg-AMC (1:200, JA7740, Merck Millipore) or a MMP fluorogenic substrate (5 μM PEPDAB0502, Biozyme) for 2 hr and examining fluorescence in the supernatant. MMP-9 activity levels were calculated as the difference in MMP fluorescence obtained in the absence and presence of an MMP-9 inhibitor (100 nM of MMP-9 inhibitor I). See Supplemental Experimental Procedures for further details.

Structural Plasticity and Spine Imaging

CA1 cells in slice cultures were patched (16–25 MΩ) with KGluconate internal solution containing Alexa Fluor 488 (0.1–0.2 mM, Thermo Fisher Scientific). Small dendritic spines (<1 μm) were targeted for MNI-glutamate spot photolysis using a 405-nm laser (Photonics). LTP was induced by pairing photolysis (1 ms) with three to five action potentials. Pairing was repeated 60 times at 5 Hz. Fractional changes in fluorescence of the spine head, standardized to

that of the underlying dendrite, were used to estimate fractional changes in spine volume. See Supplemental Experimental Procedures for further details.

Statistical Analysis

Significance was assessed using parametric tests (two-tailed paired and unpaired t test) or non-parametric tests (two-tailed Mann-Whitney test or two-tailed Wilcoxon matched-pairs test) in which a normal distribution of data could not be confirmed. One-sample z tests were used for comparisons against a known value. The Pearson correlation coefficient was used for assessing the significance of linear trends. For multiple comparisons, ANOVA and post hoc Bonferroni's tests (parametric) or the Kruskal-Wallis test with post hoc Dunn's tests (non-parametric) were used. In the text, values are quoted as mean ± SEM, and significance is quoted either at the $p < 0.05$ or $p < 0.01$ level.

SUPPLEMENTAL INFORMATION

Supplemental Information includes Supplemental Experimental Procedures, nine figures, and two movies and can be found with this article online at http://dx.doi.org/10.1016/j.neuron.2016.11.013.

AUTHOR CONTRIBUTIONS

Z.P. and N.J.E. designed the experiments. Z.P., L.M., S.J.B., M.R., R.T., A.H., and M.L.H performed and analyzed the experiments. Z.P. and L.M. wrote the manuscript. Z.P. and N.J.E. revised the manuscript. N.J.E. supervised the project.

ACKNOWLEDGMENTS

The authors thank Professor Fran Platt and Dr. Sridhar Vasudevan and their respective labs for allowing us access to their plate readers. This work was funded by the MRC (UK) (G0501572) and BBSRC (UK) (BB/5018724/1). Z.P. was funded by a junior research fellowship from Magdalen College, Oxford. M.R. was funded by a scholarship from the Rhodes Trust, and S.J.B. was funded by a scholarship from the British Pharmacological Society.

Received: April 15, 2016
Revised: September 2, 2016
Accepted: October 31, 2016
Published: December 15, 2016

REFERENCES

Arantes, R.M., and Andrews, N.W. (2006). A role for synaptotagmin VII-regulated exocytosis of lysosomes in neurite outgrowth from primary sympathetic neurons. J. Neurosci. *26*, 4630–4637.

Bak, J., White, P., Timár, G., Missiaen, L., Genazzani, A.A., and Galione, A. (1999). Nicotinic acid adenine dinucleotide phosphate triggers Ca2+ release from brain microsomes. Curr. Biol. *9*, 751–754.

Berg, I., Potter, B.V., Mayr, G.W., and Guse, A.H. (2000). Nicotinic acid adenine dinucleotide phosphate (NAADP(+)) is an essential regulator of T-lymphocyte Ca(2+)-signaling. J. Cell Biol. *150*, 581–588.

Blott, E.J., and Griffiths, G.M. (2002). Secretory lysosomes. Nat. Rev. Mol. Cell Biol. *3*, 122–131.

Brailoiu, E., Patel, S., and Dun, N.J. (2003). Modulation of spontaneous transmitter release from the frog neuromuscular junction by interacting intracellular Ca(2+) stores: critical role for nicotinic acid-adenine dinucleotide phosphate (NAADP). Biochem. J. *373*, 313–318.

Brailoiu, E., Hoard, J.L., Filipeanu, C.M., Brailoiu, G.C., Dun, S.L., Patel, S., and Dun, N.J. (2005). Nicotinic acid adenine dinucleotide phosphate potentiates neurite outgrowth. J. Biol. Chem. *280*, 5646–5650.

Brailoiu, E., Churamani, D., Pandey, V., Brailoiu, G.C., Tuluc, F., Patel, S., and Dun, N.J. (2006). Messenger-specific role for nicotinic acid adenine dinucleotide phosphate in neuronal differentiation. J. Biol. Chem. *281*, 15923–15928.

Brailoiu, G.C., Brailoiu, E., Parkesh, R., Galione, A., Churchill, G.C., Patel, S., and Dun, N.J. (2009). NAADP-mediated channel 'chatter' in neurons of the rat medulla oblongata. Biochem J. *419*, 91–97.

Calcraft, P.J., Ruas, M., Pan, Z., Cheng, X., Arredouani, A., Hao, X., Tang, J., Rietdorf, K., Teboul, L., Chuang, K.T., et al. (2009). NAADP mobilizes calcium from acidic organelles through two-pore channels. Nature *459*, 596–600.

Cang, C., Bekele, B., and Ren, D. (2014). The voltage-gated sodium channel TPC1 confers endolysosomal excitability. Nat. Chem. Biol. *10*, 463–469.

Chazotte, B. (2011). Labeling lysosomes in live cells with LysoTracker. Cold Spring Harb. Protoc. *2011*, pdb.prot5571.

Christie, B.R., Eliot, L.S., Ito, K., Miyakawa, H., and Johnston, D. (1995). Different Ca2+ channels in soma and dendrites of hippocampal pyramidal neurons mediate spike-induced Ca2+ influx. J. Neurophysiol. *73*, 2553–2557.

Davis, L.C., Morgan, A.J., Chen, J.L., Snead, C.M., Bloor-Young, D., Shenderov, E., Stanton-Humphreys, M.N., Conway, S.J., Churchill, G.C., Parrington, J., et al. (2012). NAADP activates two-pore channels on T cell cytolytic granules to stimulate exocytosis and killing. Curr. Biol. *22*, 2331–2337.

Dziembowska, M., and Wlodarczyk, J. (2012). MMP9: a novel function in synaptic plasticity. Int. J. Biochem. Cell Biol. *44*, 709–713.

Dziembowska, M., Milek, J., Janusz, A., Rejmak, E., Romanowska, E., Gorkiewicz, T., Tiron, A., Bramham, C.R., and Kaczmarek, L. (2012). Activity-dependent local translation of matrix metalloproteinase-9. J. Neurosci. *32*, 14538–14547.

Emptage, N., Bliss, T.V., and Fine, A. (1999). Single synaptic events evoke NMDA receptor-mediated release of calcium from internal stores in hippocampal dendritic spines. Neuron *22*, 115–124.

Erecińska, M., Dagani, F., Nelson, D., Deas, J., and Silver, I.A. (1991). Relations between intracellular ions and energy metabolism: a study with monensin in synaptosomes, neurons, and C6 glioma cells. J. Neurosci. *11*, 2410–2421.

Galione, A. (2015). A primer of NAADP-mediated Ca(2+) signalling: From sea urchin eggs to mammalian cells. Cell Calcium *58*, 27–47.

Galione, A., Morgan, A.J., Arredouani, A., Davis, L.C., Rietdorf, K., Ruas, M., and Parrington, J. (2010). NAADP as an intracellular messenger regulating lysosomal calcium-release channels. Biochem. Soc. Trans. *38*, 1424–1431.

Gan, L., Ye, S., Chu, A., Anton, K., Yi, S., Vincent, V.A., von Schack, D., Chin, D., Murray, J., Lohr, S., et al. (2004). Identification of cathepsin B as a mediator of neuronal death induced by Abeta-activated microglial cells using a functional genomics approach. J. Biol. Chem. *279*, 5565–5572.

Grubb, M.S., and Burrone, J. (2009). Using pHluorins to Investigate Synaptic Function (eLS).

Guse, A.H. (2012). Linking NAADP to ion channel activity: a unifying hypothesis. Sci. Signal. *5*, pe18.

Helmchen, F., Imoto, K., and Sakmann, B. (1996). Ca2+ buffering and action potential-evoked Ca2+ signaling in dendrites of pyramidal neurons. Biophys. J. *70*, 1069–1081.

Hook, G., Jacobsen, J.S., Grabstein, K., Kindy, M., and Hook, V. (2015). Cathepsin B is a New Drug Target for Traumatic Brain Injury Therapeutics: Evidence for E64d as a Promising Lead Drug Candidate. Front. Neurol. *6*, 178.

Hui, L., Geiger, N.H., Bloor-Young, D., Churchill, G.C., Geiger, J.D., and Chen, X. (2015). Release of calcium from endolysosomes increases calcium influx through N-type calcium channels: Evidence for acidic store-operated calcium entry in neurons. Cell Calcium *58*, 617–627.

Jaffe, D.B., Johnston, D., Lasser-Ross, N., Lisman, J.E., Miyakawa, H., and Ross, W.N. (1992). The spread of Na+ spikes determines the pattern of dendritic Ca2+ entry into hippocampal neurons. Nature *357*, 244–246.

Kingham, P.J., and Pocock, J.M. (2001). Microglial secreted cathepsin B induces neuronal apoptosis. J. Neurochem. *76*, 1475–1484.

Kostoulas, G., Lang, A., Nagase, H., and Baici, A. (1999). Stimulation of angiogenesis through cathepsin B inactivation of the tissue inhibitors of matrix metalloproteinases. FEBS Lett. *455*, 286–290.

Kovalchuk, Y., Eilers, J., Lisman, J., and Konnerth, A. (2000). NMDA receptor-mediated subthreshold Ca(2+) signals in spines of hippocampal neurons. J. Neurosci. *20*, 1791–1799.

Linebaugh, B.E., Sameni, M., Day, N.A., Sloane, B.F., and Keppler, D. (1999). Exocytosis of active cathepsin B enzyme activity at pH 7.0, inhibition and molecular mass. Eur. J. Biochem. *264*, 100–109.

Luzio, J.P., Bright, N.A., and Pryor, P.R. (2007a). The role of calcium and other ions in sorting and delivery in the late endocytic pathway. Biochem. Soc. Trans. *35*, 1088–1091.

Luzio, J.P., Pryor, P.R., and Bright, N.A. (2007b). Lysosomes: fusion and function. Nat. Rev. Mol. Cell Biol. *8*, 622–632.

Markram, H., Helm, P.J., and Sakmann, B. (1995). Dendritic calcium transients evoked by single back-propagating action potentials in rat neocortical pyramidal neurons. J. Physiol. *485*, 1–20.

Martinez, I., Chakrabarti, S., Hellevik, T., Morehead, J., Fowler, K., and Andrews, N.W. (2000). Synaptotagmin VII regulates Ca(2+)-dependent exocytosis of lysosomes in fibroblasts. J. Cell Biol. *148*, 1141–1149.

Masgrau, R., Churchill, G.C., Morgan, A.J., Ashcroft, S.J., and Galione, A. (2003). NAADP: a new second messenger for glucose-induced Ca2+ responses in clonal pancreatic beta cells. Curr. Biol. *13*, 247–251.

McGuinness, L., Bardo, S.J., and Emptage, N.J. (2007). The lysosome or lysosome-related organelle may serve as a Ca2+ store in the boutons of hippocampal pyramidal cells. Neuropharmacology *52*, 126–135.

Miller, M.A., Barkal, L., Jeng, K., Herrlich, A., Moss, M., Griffith, L.G., and Lauffenburger, D.A. (2011). Proteolytic Activity Matrix Analysis (PrAMA) for simultaneous determination of multiple protease activities. Integr. Biol. (Camb.) *3*, 422–438.

Mitrović, A., Mirković, B., Sosič, I., Gobec, S., and Kos, J. (2016). Inhibition of endopeptidase and exopeptidase activity of cathepsin B impairs extracellular matrix degradation and tumour invasion. Biol. Chem. *397*, 165–174.

Morgan, A.J., and Galione, A. (2014). Two-pore channels (TPCs): current controversies. BioEssays *36*, 173–183.

Morgan, A.J., Platt, F.M., Lloyd-Evans, E., and Galione, A. (2011). Molecular mechanisms of endolysosomal Ca2+ signalling in health and disease. Biochem. J. *439*, 349–374.

Morgan, A.J., Davis, L.C., Wagner, S.K., Lewis, A.M., Parrington, J., Churchill, G.C., and Galione, A. (2013). Bidirectional Ca^{2+} signaling occurs between the endoplasmic reticulum and acidic organelles. J. Cell Biol. *200*, 789–805.

Morgan, A.J., Davis, L.C., and Galione, A. (2015a). Imaging approaches to measuring lysosomal calcium. Methods Cell Biol. *126*, 159–195.

Morgan, A.J., Davis, L.C., Ruas, M., and Galione, A. (2015b). TPC: the NAADP discovery channel? Biochem. Soc. Trans. *43*, 384–389.

Mort, J.S., Recklies, A.D., and Poole, A.R. (1984). Extracellular presence of the lysosomal proteinase cathepsin B in rheumatoid synovium and its activity at neutral pH. Arthritis Rheum. *27*, 509–515.

Murphy, N., and Lynch, M.A. (2012). Activation of the $P2X_7$ receptor induces migration of glial cells by inducing cathepsin B degradation of tissue inhibitor of metalloproteinase 1. J. Neurochem. *123*, 761–770.

Nagy, V., Bozdagi, O., Matynia, A., Balcerzyk, M., Okulski, P., Dzwonek, J., Costa, R.M., Silva, A.J., Kaczmarek, L., and Huntley, G.W. (2006). Matrix metalloproteinase-9 is required for hippocampal late-phase long-term potentiation and memory. J. Neurosci. *26*, 1923–1934.

Ogata, Y., Itoh, Y., and Nagase, H. (1995). Steps involved in activation of the pro-matrix metalloproteinase 9 (progelatinase B)-tissue inhibitor of metalloproteinases-1 complex by 4-aminophenylmercuric acetate and proteinases. J. Biol. Chem. *270*, 18506–18511.

Pandey, V., Chuang, C.C., Lewis, A.M., Aley, P.K., Brailoiu, E., Dun, N.J., Churchill, G.C., and Patel, S. (2009). Recruitment of NAADP-sensitive acidic Ca2+ stores by glutamate. Biochem. J. *422*, 503–512.

Patel, S., Churchill, G.C., Sharp, T., and Galione, A. (2000). Widespread distribution of binding sites for the novel Ca2+-mobilizing messenger, nicotinic acid adenine dinucleotide phosphate, in the brain. J. Biol. Chem. *275*, 36495–36497.

Patel, S., Churchill, G.C., and Galione, A. (2001). Coordination of Ca2+ signalling by NAADP. Trends Biochem. Sci. *26*, 482–489.

Penny, C.J., Kilpatrick, B.S., Han, J.M., Sneyd, J., and Patel, S. (2014). A computational model of lysosome-ER Ca2+ microdomains. J. Cell Sci. *127*, 2934–2943.

Pišlar, A., and Kos, J. (2014). Cysteine cathepsins in neurological disorders. Mol. Neurobiol. *49*, 1017–1030.

Pitt, S.J., Funnell, T.M., Sitsapesan, M., Venturi, E., Rietdorf, K., Ruas, M., Ganesan, A., Gosain, R., Churchill, G.C., Zhu, M.X., et al. (2010). TPC2 is a novel NAADP-sensitive Ca2+ release channel, operating as a dual sensor of luminal pH and Ca2+. J. Biol. Chem. *285*, 35039–35046.

Pitt, S.J., Lam, A.K., Rietdorf, K., Galione, A., and Sitsapesan, R. (2014). Reconstituted human TPC1 is a proton-permeable ion channel and is activated by NAADP or Ca2+. Sci. Signal. *7*, ra46.

Pryor, P.R., Mullock, B.M., Bright, N.A., Gray, S.R., and Luzio, J.P. (2000). The role of intraorganellar Ca(2+) in late endosome-lysosome heterotypic fusion and in the reformation of lysosomes from hybrid organelles. J. Cell Biol. *149*, 1053–1062.

Reddy, A., Caler, E.V., and Andrews, N.W. (2001). Plasma membrane repair is mediated by Ca(2+)-regulated exocytosis of lysosomes. Cell *106*, 157–169.

Ryan, R.E., Sloane, B.F., Sameni, M., and Wood, P.L. (1995). Microglial cathepsin B: an immunological examination of cellular and secreted species. J. Neurochem. *65*, 1035–1045.

Rybalchenko, V., Ahuja, M., Coblentz, J., Churamani, D., Patel, S., Kiselyov, K., and Muallem, S. (2012). Membrane potential regulates nicotinic acid adenine dinucleotide phosphate (NAADP) dependence of the pH- and Ca2+-sensitive organellar two-pore channel TPC1. J. Biol. Chem. *287*, 20407–20416.

Sandow, A. (1952). Excitation-contraction coupling in muscular response. Yale J. Biol. Med. *25*, 176–201.

Sbai, O., Ferhat, L., Bernard, A., Gueye, Y., Ould-Yahoui, A., Thiolloy, S., Charrat, E., Charton, G., Tremblay, E., Risso, J.J., et al. (2008). Vesicular trafficking and secretion of matrix metalloproteinases-2, -9 and tissue inhibitor of metalloproteinases-1 in neuronal cells. Mol. Cell. Neurosci. *39*, 549–568.

Spruston, N., Schiller, Y., Stuart, G., and Sakmann, B. (1995). Activity-dependent action potential invasion and calcium influx into hippocampal CA1 dendrites. Science *268*, 297–300.

Wang, X.B., Bozdagi, O., Nikitczuk, J.S., Zhai, Z.W., Zhou, Q., and Huntley, G.W. (2008). Extracellular proteolysis by matrix metalloproteinase-9 drives dendritic spine enlargement and long-term potentiation coordinately. Proc. Natl. Acad. Sci. USA *105*, 19520–19525.

Wlodarczyk, J., Mukhina, I., Kaczmarek, L., and Dityatev, A. (2011). Extracellular matrix molecules, their receptors, and secreted proteases in synaptic plasticity. Dev. Neurobiol. *71*, 1040–1053.

Wu, G.Y., Deisseroth, K., and Tsien, R.W. (2001a). Activity-dependent CREB phosphorylation: convergence of a fast, sensitive calmodulin kinase pathway and a slow, less sensitive mitogen-activated protein kinase pathway. Proc. Natl. Acad. Sci. USA *98*, 2808–2813.

Wu, G.Y., Deisseroth, K., and Tsien, R.W. (2001b). Spaced stimuli stabilize MAPK pathway activation and its effects on dendritic morphology. Nat. Neurosci. *4*, 151–158.

Zhang, Z., Chen, G., Zhou, W., Song, A., Xu, T., Luo, Q., Wang, W., Gu, X.S., and Duan, S. (2007). Regulated ATP release from astrocytes through lysosome exocytosis. Nat. Cell Biol. *9*, 945–953.

Neuron, Volume *93*

Supplemental Information

Activity-Dependent Exocytosis of Lysosomes Regulates the Structural Plasticity of Dendritic Spines

Zahid Padamsey, Lindsay McGuinness, Scott J. Bardo, Marcia Reinhart, Rudi Tong, Anne Hedegaard, Michael L. Hart, and Nigel J. Emptage

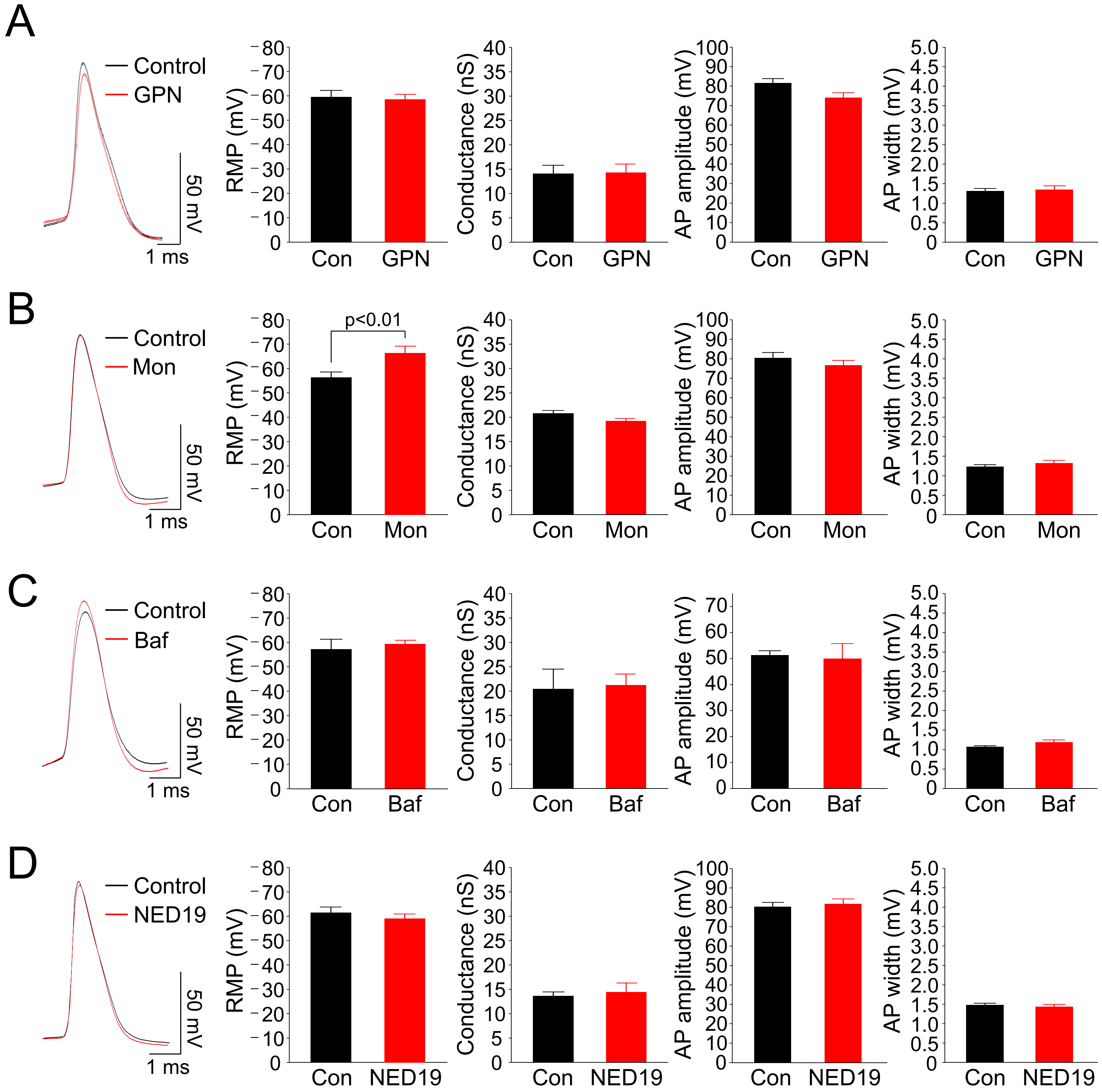

Supplementary Figure 1

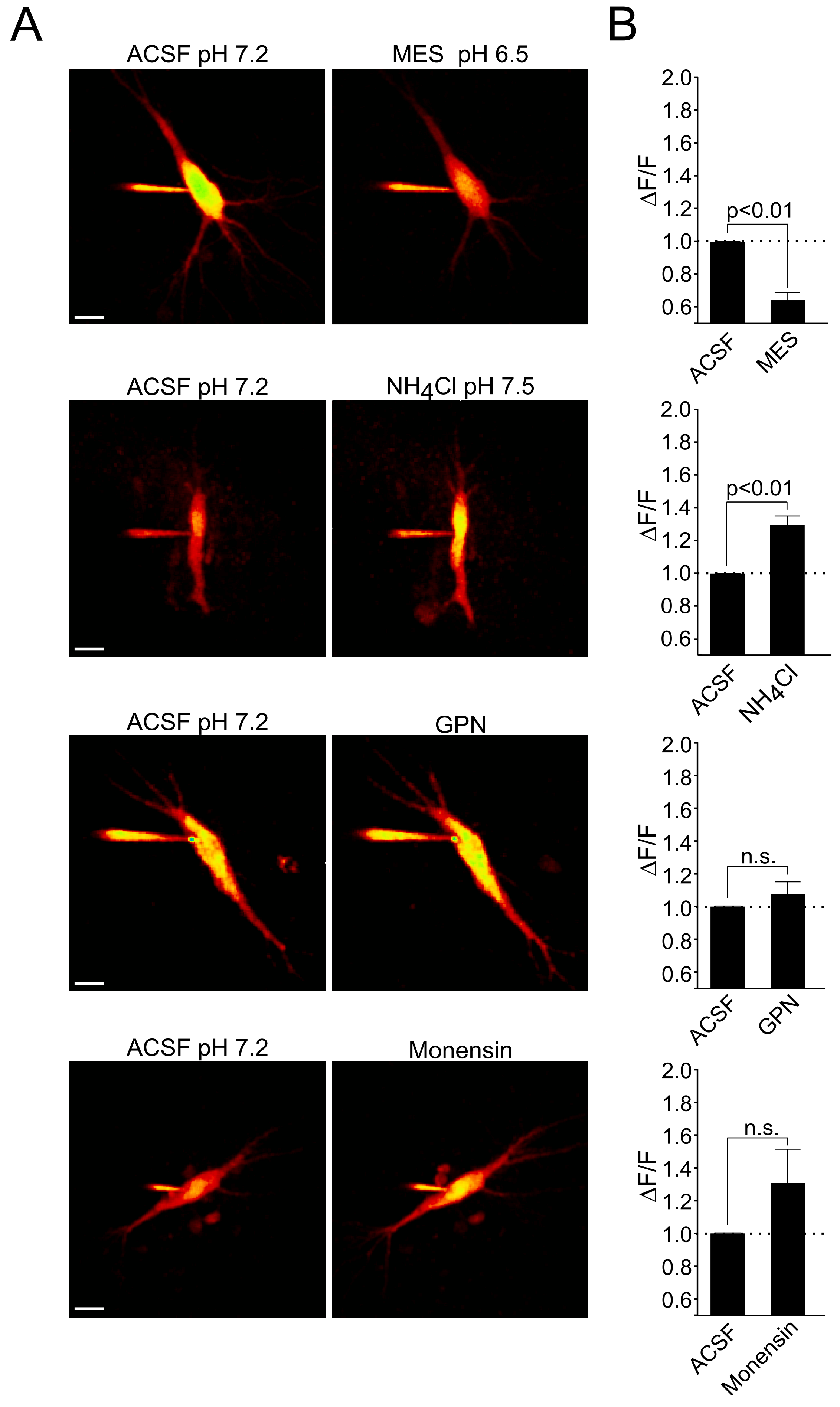

Supplementary Figure 2

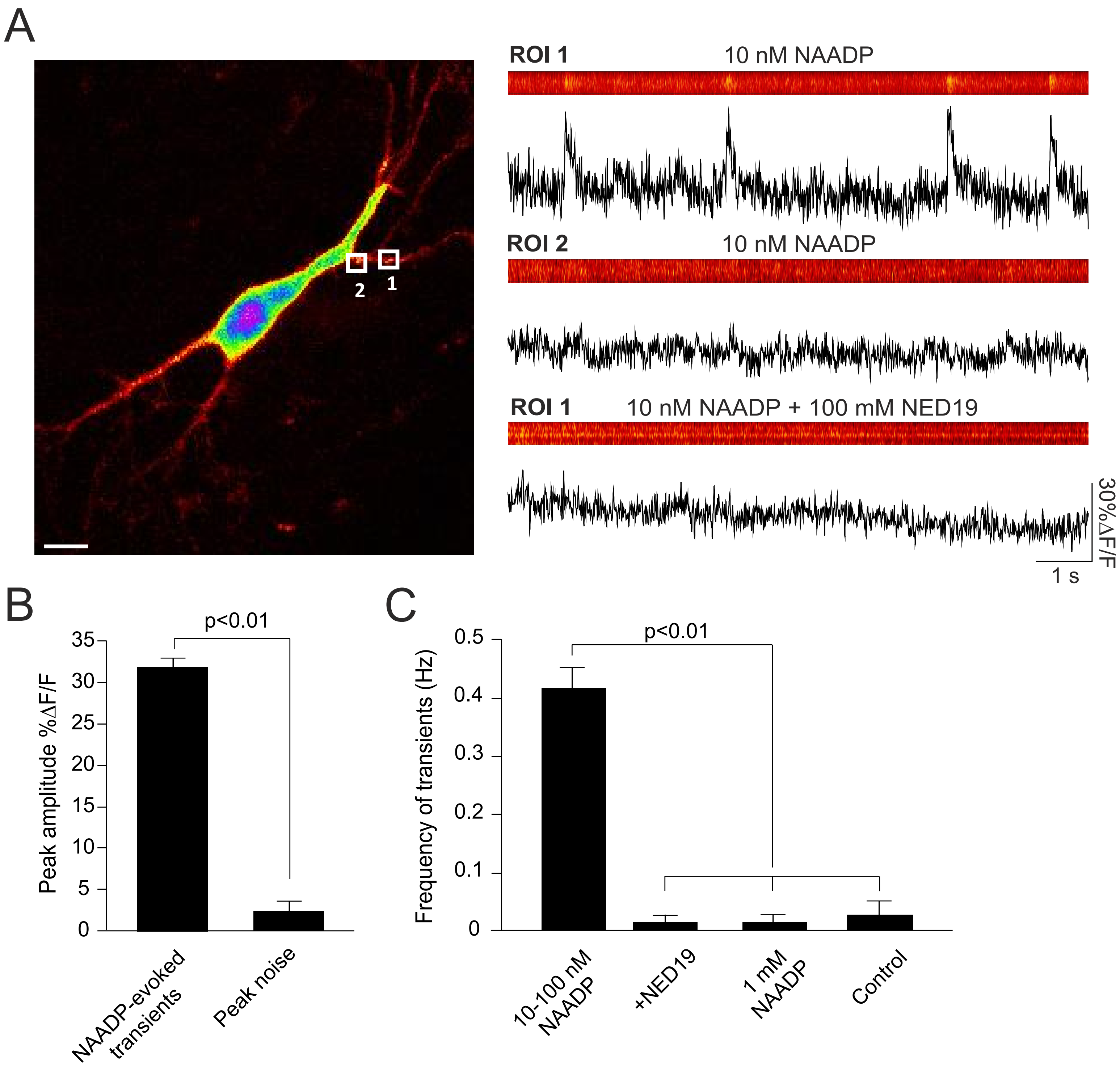

Supplementary Figure 3

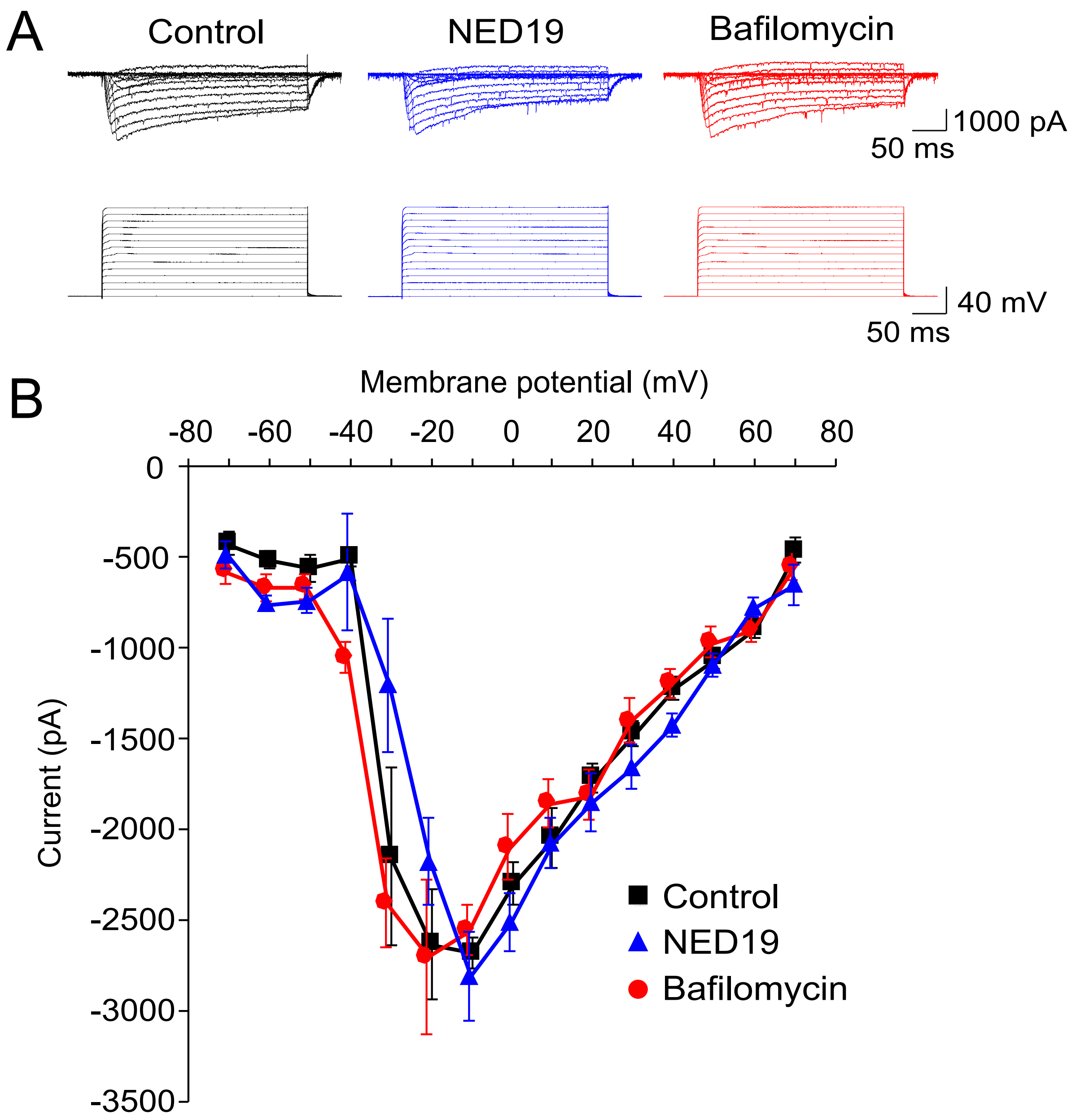

Supplementary Figure 4

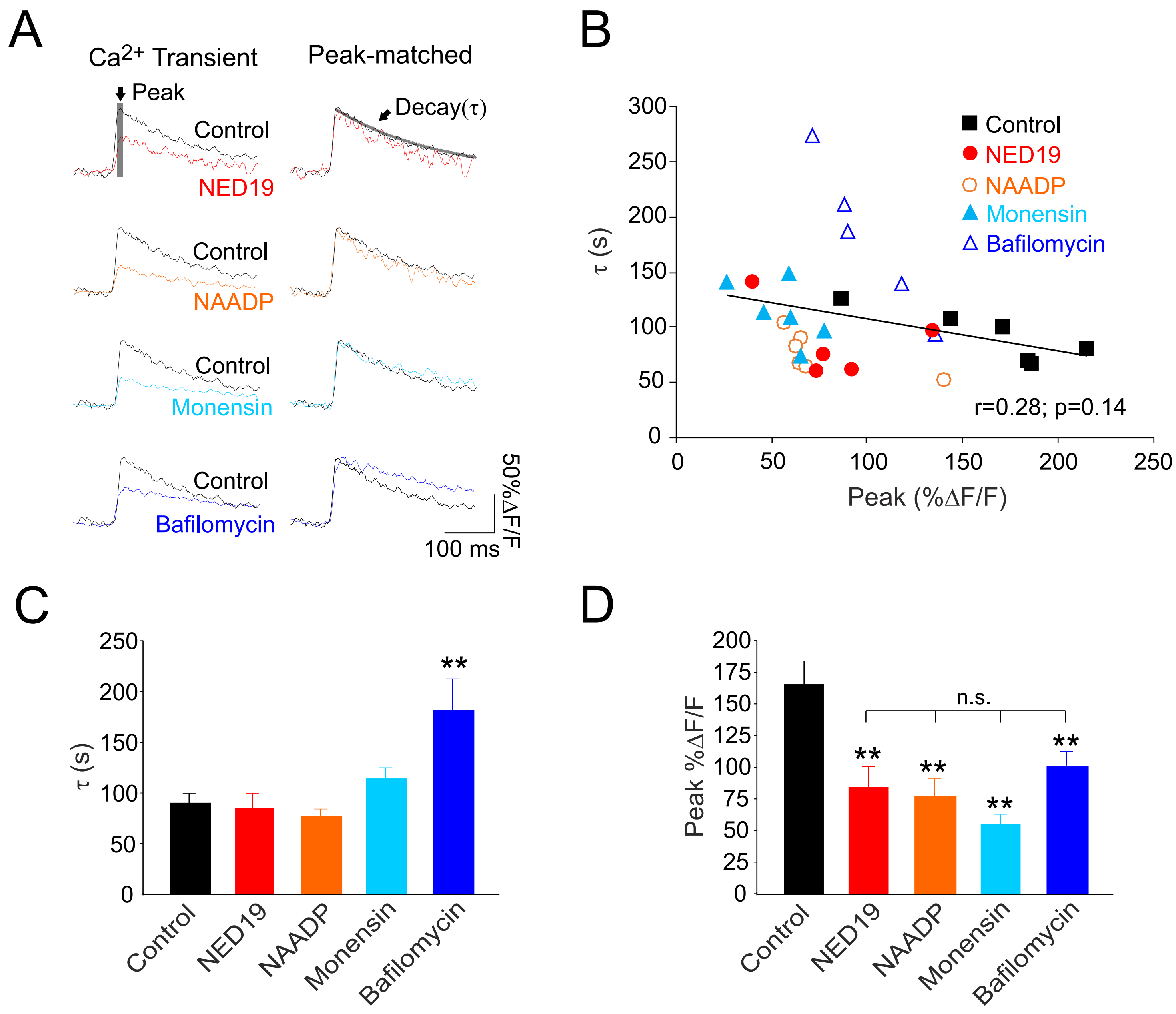

Supplementary Figure 5

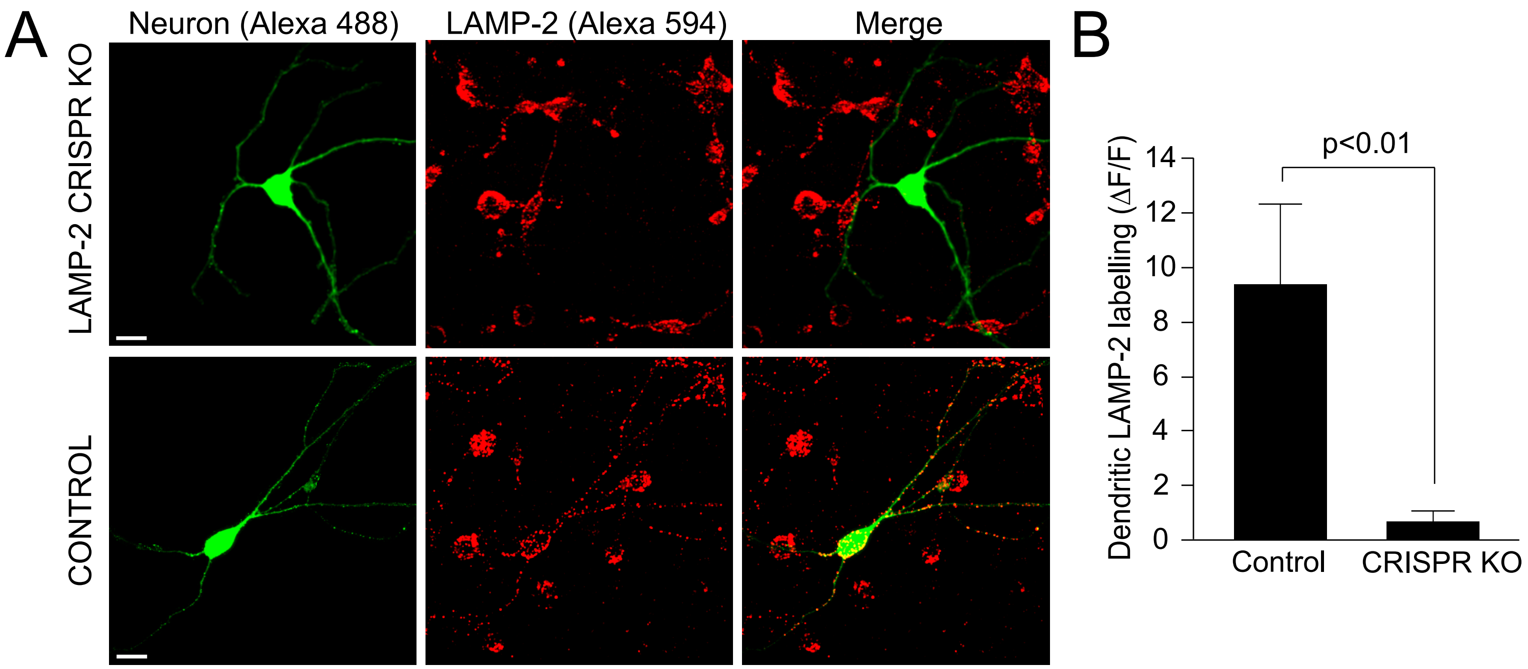

Supplementary Figure 6

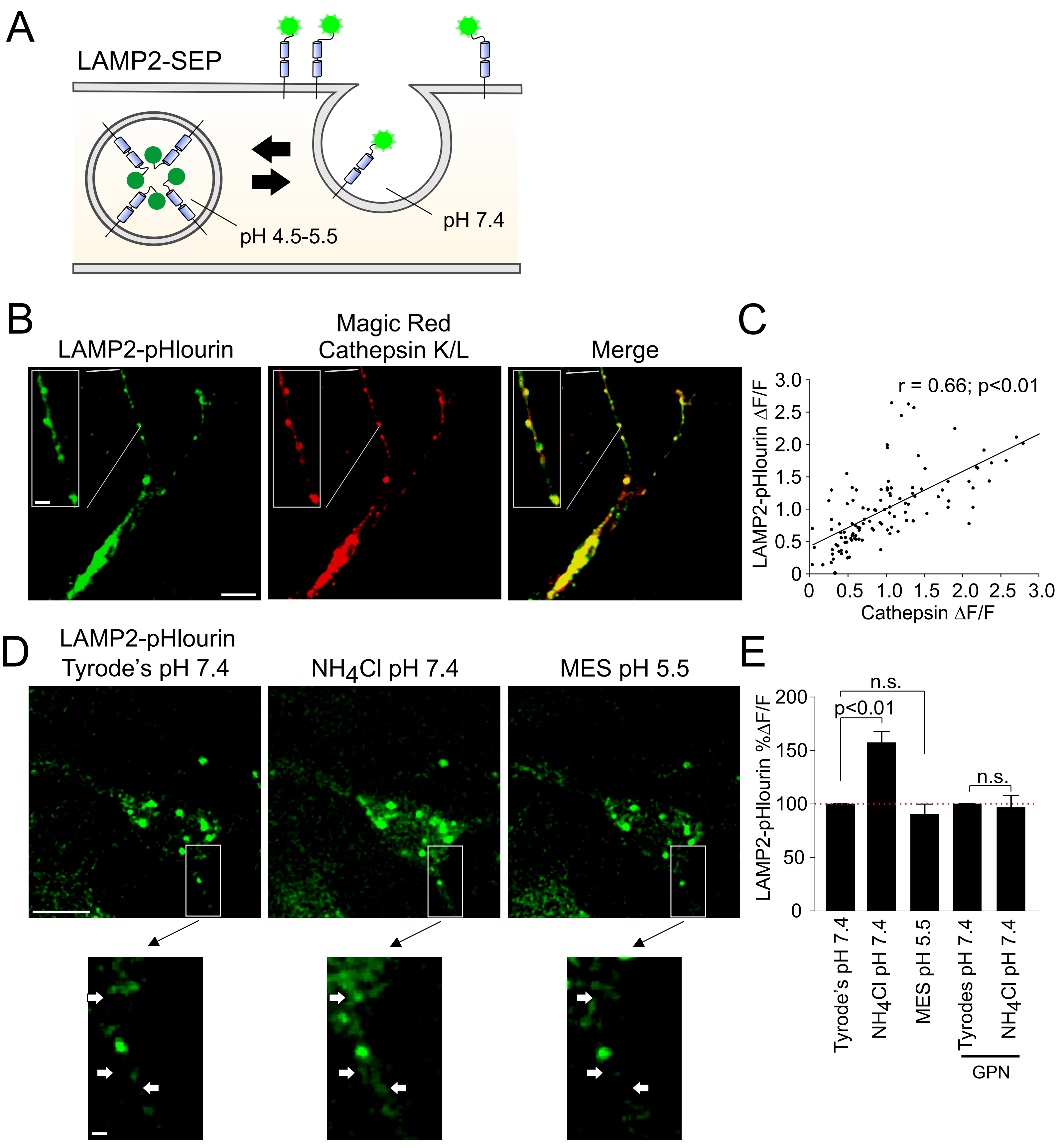

Supplementary Figure 7

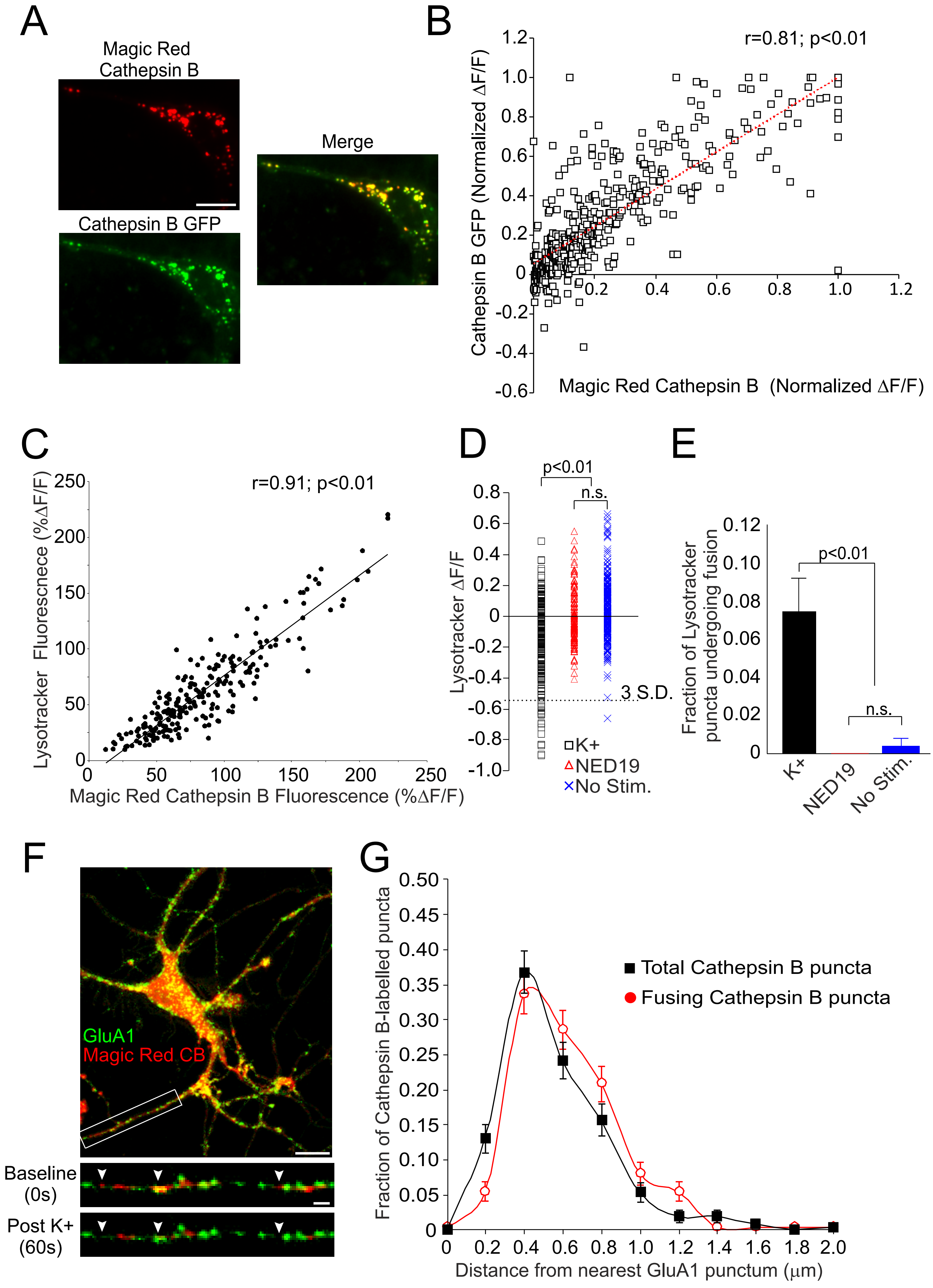

Supplementary Figure 8

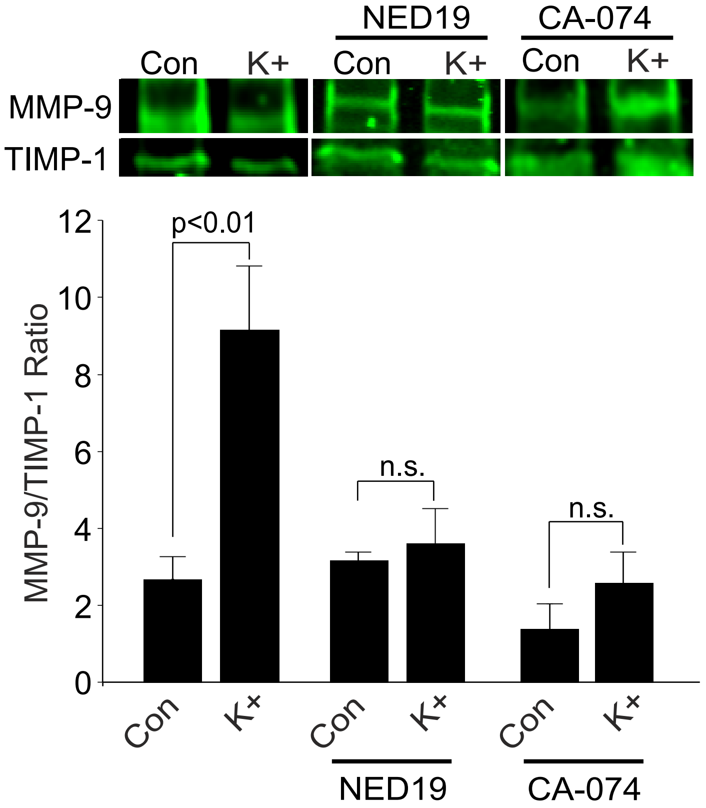

Supplementary Figure 9

Figure S1. Electrophysiological characterization of hippocampal neurons and action potential waveform in the presence of pharmacological inhibitors of lysosomal function. Related to Figure 1 and 2. Electrophysiological analysis of **(A)** GPN, **(B)** monensin (mon), **(C)** bafilomycin (baf), and **(D)** NED-19 are shown. For each drug, a sample action potential waveform is shown, along with group averages of cell resting membrane potential (RMP), membrane conductance, action potential (AP) amplitude, and AP width, calculated at AP half-height. None of these parameters are altered by the drug, with the exception of monensin, which results in significant hyperpolarization of the resting membrane potential. All pair-wise comparisons are non-significant, unless otherwise noted (n=5-7 cells/condition). Error bars represent S.E.M. Significance was assessed with Mann-Whitney tests.

Figure S2. Characterization of intracellular pH in the presence of pharmacological inhibitors of lysosomal function. Related to Figure 1 and 2. Drug-induced changes in intracellular pH were assessed using fluorescein, the fluorescence of which is pH sensitive. **(A)** Sample images of neurons patch-loaded with fluorescein (scale bars=10 µm). Images were taken in ACSF (pH=7.2) and following bath application of various solutions. Bath application of an acidic MES-buffered ACSF solution (pH 6.5) resulted in a significant reduction in fluorescence, whereas bath application of an alkaline NH_4Cl-buffered ACSF solution (pH 7.5) resulted in a significant increase in fluorescence. Fluorescein fluorescence was therefore bidirectionally sensitive to changes in pH. Bath application of GPN, which pharmacologically permeablizes the lysosome, had no substantial effect on fluorescein fluorescence. Bath application of the Na^+/H^+ ionophore monensin, however, had a tendancy an increase in fluorescence, consistent with an increase in intracellular pH that has previously been reported with the drug (Erecinska et al., 1991). This increase, however, was not significant. **(B)** Group averages are shown (n=5-6 cells/condition). Error bars represent S.E.M. Non-significant group comparisons are denoted by n.s. Significance was assessed with Mann-Whitney tests.

Figure S3. Imaging of lysosomal Ca^{2+} release in response to NAADP. Related to Figure 2 and 3. **(A)** Right. Representative image of a hippocampal pyramidal neuron (scale bar=10 µm) loaded with Oregon Green BAPTA-1 (OGB-1) and with NAADP (10-100 nM). Two regions of interest (ROI 1 and ROI 2) were examined (white boxes) along the apical dendrite by restricting laser scanning to a line along both ROIs. Left. The resulting line scans, along with their quantified fluorescence (%ΔF/F), are shown. In ROI 1, small and rapid Ca^{2+} transients were observed. These transients were often rhythmic, highly localized, and did not spread to ROI 2. The addition of the NAADP-antagonist NED-19 abolished these transients. **(B)** Average amplitude of NAADP-evoked Ca^{2+} transients (n=36 events recorded across 3 cells in 6 regions of interest), compared with peak fluorescence amplitude of noise. **(C)** Average frequency of NAADP-evoked Ca^{2+} transients (n= 6 regions of interest recorded across 3 cells/condition). In the presence of high concentrations of NAADP (1 mM), NED-19, or in the absence of NAADP (control), Ca^{2+} transients were rarely observed (<2 across experiments), and when detected, were isolated events showing no rhythmicity.

Figure S4. Voltage-gated Ca^{2+} channel currents are unaffected by inhibition of lysosomal Ca^{2+} signalling. Related to Figure 2. **(A)** Sample of voltage-gated Ca^{2+} channel (VGCC) currents recorded from CA1 neurons during 10 mV step depolarizations between -70 and +70 mV in control, NED-19 treated, and bafilomycin treated slices. **(B)** Group average current-voltage plots for peak VGCC currents (n=7-8 cells/conditions). Significance was assessed using a two-way ANOVA. VGCC currents recorded in control, NED-19, and bafilomycin treated cells were not significantly different from one another.

Figure S5. Characterization of Ca^{2+} handling in the presence of pharmacological inhibitors of lysosomal signalling. Related to Figure 2. **(A)** Ca^{2+} handling was quantified by examining the decay of back-propagating action potential (bpAP)-evoked Ca^{2+} transients recorded in CA1 dendrites. Left. Sample Ca^{2+} transients recorded across experimental conditions. Shaded box and arrow denote the window in which the peak value of the transient was calculated. The control trace (black) has been

duplicated in each example. Right. Ca^{2+} transients have been peak-matched to controls in order to calculate the time constant of decay (τ). **(B)** The peak amplitude and τ are plotted for each experiment across experimental conditions. There is no significant correlation between the two variables suggesting that measurements of drug-induced effects on peak Ca^{2+} influx are not likely to be effected by potential drug-induced effects on Ca^{2+} handling. Significance was assessed using the Pearson correlation coefficient. **(C)** Average τ is plotted across experimental conditions (n=5-7/condition). Bafilomycin led to a significant increase in τ, suggesting that it impaired the ability of the cell to extrude Ca^{2+} following the bpAP. **(D)** Average peak Ca^{2+} amplitude is plotted across experimental conditions (n=5-7/condition). All pharmacological inhibitors of lysosomal Ca^{2+} signalling reduced peak Ca^{2+} influx relative to control, independent of their effects on Ca^{2+} handling (C). Error bars represent S.E.M. Asterisks (**) denote significance at p<0.01 from control levels, unless otherwise noted. Significance was assessed using ANOVA and post-hoc Bonferroni tests.

Figure S6. Confirmation of anti-LAMP2 antibody specificity. Related to Figure 4. **(A)** (Green) Sample image of a neuron targeted for CRISPR/Cas9-mediated knock-out of LAMP-2 and a control neuron. (Red) Cultures were live-cell immunolabelled with anti-LAMP2 antibodies. Neurons expressing the CRIPSR/Cas9 plasmid showed negligible levels of surface labelling, confirming the specificity of the antibody in our experimental setup. **(B)** Quantification of LAMP-2 immunolabelling in control and CRISPR/Cas9-transfected neurons. Error bars reflect S.E.M. Significance was assessed using a Mann-Whitney test.

Figure S7. Characterization of LAMP2-SEP. Related to Figure 5. **(A)** Schematic of LAMP2-SEP. Superecliptic pHlourin (SEP) was tagged to the lumenal domain of lysosome-associated protein LAMP-2. At rest, LAMP2-SEP is quenched by the acidic intralumenal pH of the lysosome (pH=4.5-5.5), but fluoresces following lysosomal fusion with the plasma membrane, when it is exposed to the pH-neutral environment of the extracellular fluid. During endocytosis, the retrieval and re-acidification of the lysosome quenches LAMP2-SEP fluorescence. **(B)** *In vitro* characterization of LAMP2-SEP localization. Representative image of dissociated hippocampal neuron (scale bar=20 µm) transfected with LAMP2-SEP (green channel) and loaded with red Cathepsin K and L fluorogenic substrates (red channel) to identify lysosomal compartments; the use of LysoTracker red is not recommended for lysosomal labelling in co-localization studies (Freundt et al., 2007). Magnified images (scale bar=4 µm) of the dendrite (white) are shown. LAMP2-SEP co-localized with lysosomes (yellow channel). **(C)** Analysis of LAMP-2-colocalization with lysosomal compartments shown in (B) across cells (n=5 cells). There is a significant correlation (r=0.66; n=115 puncta; p<0.01) between Cathepsin-associated fluorescence and LAMP-2-pHlouin associated fluorescence. **(D)** *In vitro* assessment of LAMP2-SEP pH-sensitivity. Representative images of a dissociated hippocampal neuron transfected with LAMP2-SEP (scale bar=20 µm) along with magnified images (scale bar=2 µm) of a dendrite (white box). Application of NH_4Cl (pH 7.4) to neutralize acidic compartments increased LAMP2-SEP fluorescence and resulted in the emergence of new fluorescent puncta in the dendrites (white arrows), whereas application of acidic MES (pH 5.5) returned fluorescence to baseline levels, previously recorded in neutral Tyrode's solution (pH 7.4). These data confirm that LAMP2-SEP is pH-sensitive and trafficked to acidic organelles. **(E)** Group averages (n=5 cells) of data shown in (D). Cultures were also pre-treated with GPN, which specifically permeabilizes lysosomal stores, resulting in the dissipation of their proton gradient. Under these conditions, application of NH_4Cl (pH 7.4) had no effect on LAMP2-SEP fluorescence, confirming that increases in LAMP2-SEP fluorescence required de-acidification specifically of lysosomal compartments. Non-significant group comparisons are denoted by n.s.

Figure S8. Magic Red Cathepsin B labels Cathepsin B-containing puncta, which co-localize with LysoTracker staining and undergo fusion in the vicinity of synapses. Related to Figure 6. **(A)** Sample image of a neuron loaded with Magic Red Cathepsin B (red) and expressing Cathepsin B-GFP (scale bar=10 µm). Magic Red fluorescence localized well with GFP-labelled puncta. **(B)** Plot of Magic Red fluorescence against GFP fluorescence for labelled puncta (440 puncta imaged across 8

cells). There is a strong and significant correlation between Magic Red fluorescence and Cathepsin B-GFP fluorescence, suggesting that the intensity of Magic Red fluorescence accurately reflects the levels of Cathepsin B for a given puncta. Significance was assessed using the Pearson correlation co-efficient. **(C)** Plot of LysoTracker Green fluorescence against Magic Red fluorescence for labelled puncta (254 puncta from 5 cells) from experiments shown in Figure 6A. There is a strong and significant correlation between Magic Red fluorescence and LysoTracker Green fluorescence. Significance was assessed using the Pearson correlation co-efficient. **(D)** Activity-dependent LysoTracker de-staining is similar to that of Magic Red Cathepsin B de-staining (shown in Figure 6B,C). Change in LysoTracker fluorescence was examined after 1) K^+ treatment of cultures 2) K^+ treatment in cultures pre-treated with NED-19 and 3) unstimulated cultures (no stim.) (n=152-254 puncta from 5-6 cells/condition). Dashed line marks 3 standard deviations from average fluorescent changes recorded in unstimulated controls (no stim.). K+ stimulated cultures showed greater levels of destaining than NED-19-treated cultures or unstimulated cultures. Significance was assessed with Kruskal-Wallis and post-hoc Dunn's tests. **E)** Fraction of LysoTracker-labelled puncta undergoing fusion, as defined by a loss of fluorescence greater or equal to 3 standard deviations from the average fluorescent changes recorded in unstimulated controls (no stim.). K+ stimulated cultures showed greater fraction of fusing vesicles than NED-19-treated cultures or unstimulated cultures. Significance was assessed with z-tests (Boneferroni correction). **(F)** Confocal image of a dissociated neuron (scale bar=5 µm) labelled with Magic Red Cathepsin B substrate (red) and immunostained for GluA1 (green) to label synapses. The dendrite in the white box is magnified below (scale bar=2 µm). K^+ treatment resulted in loss of Cathepsin B-associated fluorescence from several puncta in the vicinity of GluA1 labelled puncta (white arrow heads). **(G)** Distribution of Cathepsin B puncta from GluA1 labelled puncta. The majority of Cathepsin B puncta (approx. 95%), including those undergoing activity-dependent fusion, were found within 1 µm of a GluA1 puncta.

Figure S9. Neuronal depolarization increases MMP-9/TIMP-1 ratios in a manner dependent on lysosomal Cathepsin B. Related to Figure 6. Top. Sample western blots of MMP-9 and TIMP-1 expression levels in cultured hippocampal slices. Bottom. Quantification of the MMP-9/TIMP-1 ratio (n=5-7 slices/condition). Inhibition of lysosomal Ca^{2+} signalling and fusion with NED-19 or inhibition of extracellular Cathepsin B activity with CA-074 prevented activity-induced increases in the MMP-9/TIMP-1 ratio. Error bars represent S.E.M. Significance was assessed with ANOVA and post-hoc Bonferroni tests.

Movie S1. Imaging activity-dependent mobilization of lysosomes at the cell surface. Related to Figure 4. Sample video clip of TIRF imaging experiments of lysosomes shown in Figure 4A. Two dissociated hippocampal neurons (soma are on the bottom right) were loaded with LysoTracker (green fluorescence) to label lysosomes. The experiment starts with a baseline recording. After which, the culture is stimulated with 45 mM K^+; this period is denoted with a label reading "K^+" on the top left corner of the video. During stimulation, there is a rapid emergence of fluorescence puncta throughout the dendritic arbour, which are not seen during baseline imaging. Frames were acquired at the rate of 1/s and are played at a rate of 4/s.

Movie S2. Imaging activity-dependent lysosomal fusion using LAMP2-SEP. Related to Figure 5. Sample video clip from experiments shown in Figure 5B. A dissociated hippocampal neuron (soma on the top left) was transfected with LAMP2-SEP (green fluorescence). The experiment starts with a period of photobleaching (denoted by the label "photobleach" on the top right corner of the clip), in order to eliminate baseline fluorescence of LAMP-2 that is already present on the cell surface. Following photobleaching, there is a baseline recording, after which the culture is stimulated with 45 mM K^+ (denoted by the label "K^+" on the top right corner of the clip). During stimulation, there is a rapid emergence of fluorescence puncta throughout the dendritic arbour, which are not seen during baseline imaging. Frames were acquired at the rate of 1/s and are played at a rate of 4/s.

Supplemental Experimental Methods

Acute hippocampal slices

Acute hippocampal slices (350-400 µm) were prepared from 2-3 week old male Wistar rats (Harlan). A sucrose-based ACSF solution (in mM: 85 NaCl, 65 sucrose, 26 $NaHCO_3$, 10 glucose, 7 $MgCl_2$, 2.5 KCl, 1.2 NaH_2PO_4, and 0.5 $CaCl_2$; pH=7.2-7.4) was used for the dissection. The whole brain was sliced along a coronal plane using a Microm HM 650V vibratome (Thermo Scientific), and dorsal hippocampi was dissected out from each slice. Hippocampal slices were recovered for at least 1 hour prior to use at room temperature in ACSF (in mM: 120 NaCl, 2.5 KCl, 2 $CaCl_2$, 1 $MgCl_2$, 1.2 NaH_2PO_4, 26 $NaHCO_3$, and 11 glucose; pH=7.2-7.4), bubbled with 95% O_2 and 5% CO_2. Slices were then transferred to the recording chamber and perfused (3 mL/min) with heated (32-34°C) and carbogenated (95% O_2 and 5% CO_2) ACSF, containing ascorbic acid (0.2 mM) and Trolox (1 mM) to minimize photodynamic damage during imaging.

Cultured hippocampal slices

Transverse 350µm hippocampal slice cultures were prepared from male Wistar rat pups, postnatal day 7 (Harlan UK) as previously described (Emptage et al., 1999). Slices were maintained 7-14 days prior to use at 37°C and 5% CO_2. Culture medium was replaced every 2-3 days and was composed of 50% Minimum Essential Media, 25% heat-inactivated horse serum, 23% Earl Balanced Salt Solution, and 2% B-27 (Invitrogen) with added glucose (6.5 g/L). During experimentation, slices were perfused (1-2mL/min) with heated (32-34°C) and carbogenated (95% O_2 and 5% CO_2) ACSF composed of (in mM): 145 NaCl, 16 $NaHCO_3$, 11 glucose, 2.5 KCl, 2-3 $CaCl_2$, 2 $MgCl_2$, 1.2 NaH_2PO_4 (pH=7.2-7.4). Additionally, 0.2 mM ascorbic acid and 1 mM Trolox were added to minimize photodynamic damage during imaging

Dissociated hippocampal cultures

Dissociated rat hippocampal neurons were prepared from E18 or P1 male Wistar pups. Hippocampi were dissected in cold Hank's Balanced Salt Solution (ThermoFisher) containing no Mg^{2+} or Ca^{2+}. Meninges were removed and tissue was incubated with trypsin (0.05%) at 37°C for 10-15 minutes. Hippocampi was then rinsed and re-suspended in serum-containing culture medium, comprising of NeuroBasal-A supplemented with 2% heat-denatured fetal calf serum, 2% B27 supplement, 1% GlutaMAX (200 mM stock), and 1% penicillin/streptomycin (10 000U/mL stock) (Thermo Fisher). The tissue was then gently triturated 30 times using a standard P1000 pipette tip, and viable cells counted in Trypan Blue (0.2%) using a hemocytometer. Cells were plated onto 18mm coverslips, pre-coated with poly-D-lysine (0.1 mg/mL) and fibronectin (0.05 mg/mL), at a density of 30 000-60 000 cells/coverslip. Hippocampal neurons were left to adhere to the coverslips for approximately 2 hours, before being placed in 12-well plates with 2mL/well of fresh serum-containing medium. Cultures were fed twice a week by replacing half of the medium with serum-free medium comprising of NeuroBasal-A supplemented with 2% B27 supplement and 1% GlutaMAX (200 mM stock). Cultures were used at DIV 10-21 and imaged at room temperature in Tyrode's buffer (in mM: 128 NaCl, 5 KCl, 1 $MgCl_2$, 2 $CaCl_2$, 4.2 $NaHCO_3$, 20 glucose and 15 HEPES buffer; pH=7.2-7.4). K^+ stimulation was carried out using a Tyrode's buffer containing either 30 or 45 mM K^+ (in mM: 88 or 103 NaCl, 45 or 30 KCl, 1 $MgCl_2$, 2 $CaCl_2$, 4.2 $NaHCO_3$, 20 glucose, and 25 HEPES buffer; pH=7.2-7.4).

Confocal imaging

Confocal imaging was conducted on an Olympus BX50WI microscope equipped with a 60x water immersion objective (NA=0.9 or 1.1) and a BioRad Radiance 2000 confocal scanhead (BioRad/Zeiss) using a 488 nm argon laser and/or a 543 nm helium-neon laser. Images were acquired using Lasersharp software either as xy images, z-stacks, or line scans (xt). Quantitative assessment of the image data was performed offline in Image J (NIH). Fluorescence were either calculated as absolute changes measured

in arbitrary units after background subtraction, or as fractional changes (%ΔF/F=100 x (F-$F_{initial}$)/($F_{initial}$-$F_{background}$)).

Total Internal Reflection Fluorescence Microscopy (TIFRM)

Dissociated hippocampal neurons (DIV 14-21) were labelled with LysoTracker or LAMP2-SEP and imaged using TIRFM (Cairn Research UK) with a 60X water immersion objective (Olympus; NA=0.90), CCD camera (Andor Luca) and Andor SOLIS software. TIRFM images were recorded over a time-course of 45-75 seconds with an exposure taken each second, during which a 45 mM K^+ Tyrode's solution was applied. Images were analysed using Image J as fractional changes (%ΔF/F=100 x (F-$F_{initial}$)/($F_{initial}$-$F_{background}$)).

Ca^{2+} imaging and electrophysiology

Hippocampal CA3 or CA1 pyramidal cells in acute or cultured hippocampal slices were recorded from using either sharp microelectrodes (70-120MΩ) or high-resistance patch electrodes (16-25 MΩ) to minimize intracellular dialysis. Sharp microelectrodes were tip filled with the Ca^{2+}-sensitive dye Oregon Green 488 BAPTA-1 (0.5-1 mM; Thermo Fisher) dissolved in 200 mM potassium acetate, and back-filled with 400 mM potassium acetate. Patch electrodes contained Oregon Green 488 BAPTA-1 (0.1-0.2 mM) dissolved in standard internal solution (in mM: 135 KGluconate, 10 KCl, 10 HEPES, 2 $MgCl_2$, 2 Na_2ATP and 0.4 Na_3GTP; pH=7.2-7.4). Cells were filled with fluorescent indicator, and regions of apical dendrite, within 100 µm of the soma, were visualised by confocal laser scanning microscopy using a 488 nm argon laser. The scan axis was aligned to bisect a dendrite of interest and line-scans consisting of 256 successive sweeps, at 2 ms intervals, were acquired, during which back-propagating action potentials (bpAPs) were triggered with somatic current injection (1-2 nA, 5-10 ms) and recorded with an AxocLAMP-2B amplifier (Axon Instruments). At least 3 bpAPs, evoked at 15-30 second intervals, were sampled both before and following pharmacological manipulation. In some experiments, 3 line scans were additionally acquired during a 200 ms depolarization pulse of 2-3nA, which depolarized the cell to >20 mV. Quantitative assessment of the image data was performed off-line in Image J (NIH). The amplitude of bpAP-evoked Ca^{2+} transients were calculated as percentage fractional change in fluorescence: %ΔF/F=100 x (F-$F_{initial}$)/($F_{initial}$-$F_{background}$). The decay constant (tau) was calculated by first peak-matching Ca^{2+} transients to control, and then fitting the falling phase of the transient (200 ms time window following its peak) with an exponential decay function. Membrane conductance was measured from a current pulse that was delivered throughout the experiment. Action potential amplitude and width at half-height were calculated using Axoclamp software.

VGCC current recordings

VGCC currents were recorded in whole-cell patch clamp from CA1 pyramidal neurons. Patch electrodes (4-6 MΩ electrodes) contained a cesium based internal solution (in mM: 115 Cs-methanesulphonate, 25 TEA-Cl, 10 HEPES, 4 MgATP, 1 EGTA and 0.4 Na_3GTP; pH=7.2-7.4). Recordings were conducted in standard ACSF solution, containing 2 mM $CsCl_2$, 1 mM 4-AP, and 2 µM TTX in order to block voltage-gated K^+ and voltage-gated Na^+ channels. Currents were recorded during 10 mV voltage steps, 400 ms in duration, from -70 mV to + 70 mV. For each voltage step, leak and capacitive currents were determined by averaging the currents recorded during 4 negative voltage steps, each with 1/4th the amplitude of the test pulse, and subtracted from the test pulse. The liquid junction potential was experimentally measured and subtracted online. Capacitance was compensated by 70%. Access resistance (<20 MΩ) was monitored throughout the recordings. Experiments were omitted if access resistance increased greater than 25 MΩ or varied more than 20% during the recording.

NAADP-evoked Ca^{2+} transients

Lysosomal Ca^{2+} transients were evoked in hippocampal pyramidal neurons in slice culture. CA3 or CA1 neurons were loaded with NAADP (10-1000 nM) and Ca^{2+}-sensitive dye Oregon Green 488 BAPTA-1 (50-100 µM). Cells were imaged with confocal microscopy using a 488 nm argon line.

Regions of dendrite were scanned for 10 seconds at a time to record spontaneous NAADP-evoked Ca^{2+} transients. Experiments were conducted in the presence of 1 µM TTX to prevent activity-dependent Ca^{2+} signalling from contaminating lysosomal Ca^{2+} release.

Synaptogmin VII recombinant protein

The cytosolic domain (C-350 amino acids) of human synaptotagmin VII (sytVII) recombinant protein was commercially obtained (513376; Novoprolabs). The protein was purified and concentrated (100-200 µg/mL) in standard patch internal solution using protein concentrators (10kDa cut off; Thermofisher). sytVII was loaded into neurons by dialysis through a patch electrode (7-10 MΩ) for 5-10 minutes prior to the experiment. The patch was then removed except in structural plasticity experiments.

Structural plasticity and spine imaging

Structural plasticity was examined in hippocampal slice cultures. CA1 pyramidal neurons were recorded using a sharp patch electrode (16-25 MΩ) to minimize intracellular dialysis, and loaded with Alexa Fluor 488 (0.1-0.2 mM; Thermo Fisher) for visualization purposes. A dendritic spine with a head diameter of <1 µm within 100 µm of the soma was targeted for glutamate photolysis using a 405 nm laser (Photonics) equipped with a rapid shutter (LS6; Uniblitz). MNI glutamate (10 mM; Tocris) was focally delivered through a glass pipette (4-8 MΩ) using a picosprtizer (custom made) and uncaged in a ~1 µm-diameter spot above the spine head with a ~1 ms laser pulse. Laser intensity (0.5-2 mW) was adjusted to trigger a ~1 mV EPSP. Photolysis was paired with a 50 ms somatic current injection (2nA) in order to trigger 3-5 action potentials at 50-100 Hz, with the first action potential following uncaging by 7-10 ms. Pairing was repeated 60 times at 5 Hz. Images of the target and neighbouring spines were acquired with 512 x 512 resolution as a z-stack with a step size of 0.5 µm at -5, -2.5, 0, 1, 15, 30, 45, and 60 minutes post-pairing. In some experiments, freshly prepared natural human Cathepsin B (5 µg/mL; abcam) or recombinant human MMP-9 (5 µg/mL; Merck Millipore) was applied 10 minutes after the start of paired stimulation. Cathepsin B was bath applied whilst MMP-9 was locally perfused through a glass pipette (4-8 MΩ) using a picosprtizer (custom made). Fractional changes ($\%\Delta F/F = 100 \times (F-F_{initial})/(F_{initial}-F_{background})$) in fluorescence of the spine head, standardized to that of the underlying dendrite, were used to estimate fractional changes in spine volume, as previously described (Matsuzaki et al., 2004, Kasai et al., 2008).

Fluorescein-based pH imaging

To examine drug-induced changes in intracellular pH, CA1 pyramidal neurons in hippocampal slice cultures were patched and loaded with 50 µM fluorescein (Sigma), which exhibits pH-dependent changes in fluorescence. Fluorescence was imaged in normal ACSF and following the addition of either a NH_4Cl buffer (in mM: 95 NaCl, 50 NH_4Cl, 16 $NaHCO_3$, 11 glucose, 2.5 KCl, 2-3 $CaCl_2$, 1-2 $MgCl_2$, 1.2 NaH_2PO_4; pH=7.5), a MES buffer (in mM: 130 NaCl, 30 MES, 16 $NaHCO_3$, 11 glucose, 2.5 KCl, 2-3 $CaCl_2$, 1-2 $MgCl_2$, 1.2 NaH_2PO_4; pH=6.5), or ACSF containing either GPN or monensin.

Lipofectamine transfection

Dissociated primary hippocampal cell cultures (DIV 7) were transfected with genetic constructs using Lipofectamine-2000 (Thermo Fisher) that had been pre-incubated with DNA at a ratio of 2:1. The complex of Lipofectamine and DNA was applied to cells for 1 hour in 1 mL of medium before being replaced with conditioned media. Cells were imaged at DIV 10-14.

Lysosomal labelling

To label lysosomes, dissociated neuronal cells were incubated with 50-75 nM LysoTracker Green or Red (Thermo Fisher) and/or 0.4% of fluoregenic Magic Red Cathepsin K and L substrates (ImmunoChemistry Technologies) in the dark for 1-2 hours at 37°C and 5% CO_2 prior to imaging, as per manufacturer's instructions. For visualization in cultured slice, single CA3 or CA1 neurons were loaded with Alexa Fluor 488 (0.1-0.2 mM) using a patch electrode.

Live-cell LAMP-2 immunolabelling

Live-cell immunolabelling of LAMP-2 was used to assess activity-dependent lysosomal fusion. Dissociated neuronal cultures (DIV 14-21) were first incubated with an unlabelled rabbit polyclonal antibody targeting the lumenal epitope of LAMP-2 (H-207; Santa Cruz Biotechnology) for 1-2 hours in order to block any LAMP-2 epitopes already present at the cell surface. The antibody was used at a concentration of 1:20, diluted in Tyrode's buffer with 2% fetal calf serum (FCS). Cultures were then washed with the same Tyrode's buffer with 2% FCS before a fluorescently-labelled variant of the same LAMP-2 antibody was applied at the same concentration (1:20). Fluorescent labelling was achieved by biotinylating the antibody (biotinylation kit; Abcam) and pre-treating it with streptavidin conjugated to Alexa Fluor 488 (1:100) (Thermo Fisher). Cultures were exposed to either standard Tyrode's buffer with 2% FCS, or to Tyrode's buffer containing 45 mM K^+ for 60 seconds. Cultures were then immediately placed on ice to prevent the endocytosis of fused lysosomes and subsequent enzymatic degradation of internalized antibodies. Cells were washed again with Tyrodes buffer containing 2% FCS and subsequently imaged using confocal microscopy in standard, ice-cold Tyrode's buffer.

The specificity of the LAMP-2 antibody was assessed by examining immunolabelling LAMP-2 knock-out (KO) cells. KO cells were generated by transfecting primary hippocampal cultures (DIV 7) with a CRISPR/Cas9 lentivector (K6833205; NBS Biologicals LTD) containing a targeting sequence (AGCCCGTAACCGGAGAGAGG) for rat LAMP-2. Cells were imaged using confocal microscopy 10 days later.

LAMP2-SEP

Superecliptic pHluorin (SEP) was amplified from template DNA, kindly provided by Dr Colin Akerman, (University of Oxford). NheI and BspE1 restriction sites were used to clone the SEP tag into the pEGFP-C1 vector (Clontech) in place of EGFP, creating a pSEP-C1 vector. LAMP-2 DNA was amplified from a mouse brain cDNA library (Life technologies) and subcloned into pSEP-C1 vector using EcoRI/SacII restriction sites tagging SEP to the N-terminus of LAMP-2.

Dissociated primary hippocampal cell cultures were transfected with LAMP2-SEP. The localization of LAMP2-SEP was assessed by loading cells with Magic Red Cathespin K and L fluorescent substrates (ImmunoChemistry Technologies) as per manufactures instructions, and examining the co-localization of red and green fluorescence with confocal microscopy. To assess the pH sensitivity of the construct, transfected cells were imaged in standard Tyrode's buffer, MES-based buffer (in mM: 128 NaCl, 5 KCl, 1 $MgCl_2$, 2 $CaCl_2$, 4.2 $NaHCO_3$, 20 glucose and 25 MES; pH=5.5), and NH_4Cl-based buffer (in mM: 78 NaCl, 50 NH_4Cl, 5 KCl, 1 $MgCl_2$, 2 $CaCl_2$, 4.2 $NaHCO_3$, 20 glucose and 25 HEPES buffer; pH=7.4).

For assessment of activity-dependent lysosome fusion, LAMP2-SEP transfected neurons were imaged either using TIRFM or confocal microscopy with a 60X water immersion objective (NA=0.90), CCD camera (Andor Luca) and Andor SOLIS software. TIRFM images were recorded over a time-course of 75 seconds with an exposure taken each second, during which a 45 mM K^+ Tyrode's solution was applied. Prior to imaging, LAMP2-SEP fluorescence was photobleached to minimize basal fluorescence in the dendrites. In some experiments, activity was driven electrically by using field stimulation (40 V, 1 ms) to deliver 300 action potentials at 5Hz via a pair of platinum bipolar electrodes, spaced 1 cm apart. Bafilomycin (1 µM) was acutely applied during stimulation to prevent re-acidification of retrieved lysosomes.

Cathepsin B assays

To assess Cathepsin B release in dissociated hippocampal neurons, cells were first loaded with 0.4% Magic Red Cathepsin B fluoregenic substrate (ImmunoChemistry Technologies) and incubated for 1-2 hours at 37°C and 5% CO_2, as per manufacturer's instructions. To confirm correct loading of the

substrate, the distribution of labelled puncta was examined in neurons transfected with Cathepsin B-GFP (pCT-Lyso-GFP; Cambridge Bioscience), and were found to co-localize strongly with GFP labelled compartments. Following loading, cultures were then stimulated with 45 mM K^+ Tyrode's solution and puncta were examined for loss of Magic Red fluorescence using confocal microscopy. Puncta exhibiting a fluorescence loss greater than 3 standard deviations from the average fluorescence recorded in unstimulated control cultures were said to have undergone fusion. To examine the distribution of Cathepsin B puncta in the vicinity of synapses, experiments were repeated following live-cell immunolabelling of GluA1-containg receptors. Dissociated neurons were incubated with rabbit anti-GluA1 antibody (PC246 Merck Millipore; 1:50 in Tyrode's solution containing 2% FCS) for 1 hour. Cultures were then washed with Tyrode's solution containing 2% FCS and incubated with goat anti-rabbit secondary antibodies conjugated to Alexa Fluor 488 (Abcam: ab150129; 1:50 in Tyrode's solution containing 2% FCS) for 1 hour, and then washed with Tyrode's solution containing 2% FCS. The shortest distance of Cathepsin B puncta to the nearest GluA1-stained puncta was calculated using ImageJ and Excel macros.

To examine activity-dependent Cathepsin B release in hippocampal slice cultures, slices were first washed in Tyrode's solution and then incubated for 2 hours at 37°C and 5% CO_2 with 200 µL Tyrodes solution. The Tyrodes solution contained either TTX (2 µm) to minimize neuronal activity or 30 mM K^+ to augment neuronal activity. Following the incubation, Cathepsin B levels were assessed in 100µL of the supernatant using a colourimetric human Cathepsin B ELISA kit (ab119684; Abcam), as per manufacture's instructions. Absorbance was measured at 450 nm using a plate reader and expressed in arbitrary units after subtraction of blank controls.

To examine Cathepsin B activity in hippocampal slice cultures, slices were first washed in Tyrode's solution and then incubated for 2 hours at 37°C and 5% CO_2 in 200 µL Tyrodes solution containing the Cathepsin B fluoregenic substrate Z-Arg-Arg-AMC (1:200; JA7740; Merck Millipore). During the incubation, slices were treated either with TTX (2 µm) to minimize neuronal activity or 30 mM K^+ to augment neuronal activity. Following the incubation, fluorescence of 100 µL of the supernatant was assessed using a plate reader (360 nm excitation/ 460 nm emission). Fluorescence was expressed in arbitrary fluorescent units (AFU) after subtraction of blank controls.

MMP activity assay

Matrix metalloproteinase (MMP) activity was assessed using a MMP fluorogenic substrate (PEPDAB0502; Biozyme), which despite being advertised as a specific MMP-9 fluoregenic substrate, actually shows relatively broad selectivity for a number of MMPs (see (Miller et al., 2011)). Cultured hippocampal slices were incubated with 5 µM of the substrate at 37°C and 5% CO_2 for 2 hours in 200 µL of normal Tyrode's solution or a 30 mM K^+ Tyrode's solution. Fluorescence of 100 µL of solution was assessed using a plate reader (480 nm excitation/520 nm emission). Fluorescence was expressed in arbitrary fluorescent units (AFU) after subtraction of blank controls. For assessment of MMP-9 activity under a particular experimental condition, 100 nM of MMP-9 Inhibitor-I was included in half of the experiments. The average fluorescence in the absence and presence of the inhibitor was subtracted to obtain a measure of MMP-9-associated activity. Although MMP-9 Inhibitor I is a potent inhibitor of MMP-9 (K_d=5 nM), it has been reported to act on MMP-13, albeit with 20-fold less affinity (Levin et al., 2001). To rule out the confounding effects of MMP-13, we repeated our experiments in the presence of an MMP-13 selective inhibitor (100 nM WAY 170523). Under these conditions we were still able to robustly detect MMP-9 activity (AFU=1317.91±318.87; n=11 slices; $p<0.01$) following K^+ stimulation, which we were still able to abolish this activity using either CA-074 (AFU=-235.70±377.55; n=8 slices; vs. K^+: $p<0.01$) or NED-19 (AFU=152.32±286.55; n=16 slices; vs. K^+: $p<0.01$).

Western Blot

MMP-9 and TIMP-1 expression levels were examined in cultured hippocampal slices. Slices were first washed in Tyrode's solution and then incubated for 2 hours at 37°C and 5% CO_2 in 200 µL Tyrodes solution, either containing TTX (2 µm) to minimize neuronal activity or 30 mM K^+ to augment neuronal activity. Slices were then homogenised in 24 µL of RIPA buffer. The lysate was boiled at 95 ˚C in 4x Laemmli sample buffer (Biorad) containing 10% 2-mercaptoethanol for 5 minutes and loaded onto a 4-20% polyacrylamide precast gel (Biorad) in standard SDS-containing Tris-glycine running buffer. Since MMP-9/TIMP-1 ratios were being analyzed instead of absolute MMP-9 or TIMP-1 levels, the concentration of protein loaded per well was not controlled for. Gels were then transferred onto nitrocellulose membranes (Whatman) in a Tris-glycine transfer buffer (ThermoFisher). Membranes were blocked with 3% BSA in TBS-T (Tris-buffered saline + Tween 20) and incubated with rabbit primary antibodies against MMP-9 (1:300; ab13458; Merck Millipore) and TIMP-1 (1:200; H-150; Santa Cruz) for 1 hour at room temperature. Proteins were detected with a goat-anti-rabbit IgG conjugated to IRDye® 800 (Li-Cor, 1:20000, 1 h at room temperature). Images were analyzed ImageJ software (National Institutes of Health). Fluorescence intensity of TIMP-1 and MMP-9 protein bands were calculated following background subtraction, and expressed as a ratio (MMP-9/TIMP-1).

Drugs

Ryanodine (20 µM final; Abcam), thapsigargin (15 µM final; Abcam), FCCP (1 µM final; Tocris), GPN (200 µM final; Sigma), trans-NED-19 (10-100 µM final; Tocris), bafilomycin (1-4 µM final; Abam), CA-074 (1 µM final; Tocris), WAY 170523 (100 nM final; Tocris), and MMP-9 Inhibitor-1 (100 nM final; Calbiochem) were dissolved in dimethyl sulfoxide (DMSO). Monensin (20 µM final; Sigma) was dissolved in 50% DMSO/50% methanol. Trolox (1 mM final; Sigma) was dissolved in 100% ethanol. NAADP (10 µM-10 mM final; Sigma), TTX (1-2 µM final; abcam), 4-AP (100 µM final; Sigma); $NiCl_2$ (100 µM final; Sigma), and $CdCl_2$ (100 µM final; Sigma) were dissolved in water. EGTA (1 mM final; Sigma) and ascorbic acid (0.2 mM) were dissolved directly in ACSF. Drugs were bath applied for 10 minutes prior to imaging with the exception of NAADP, which was applied in the internal solution and allowed to dialyze into the cell for 10-20 minutes prior to imaging, and bafilomycin and NED-19, which were applied for at least 1 hours prior to imaging unless otherwise indicated. 0 Ca^{2+} conditions were achieved by washing slices with ACSF solution comprising of 145 mM NaCl, 16 mM $NaHCO_3$, 11 mM glucose, 2.5 mM KCl, 0 mM $CaCl_2$, 4 mM $MgCl_2$, and 1.2 mM NaH_2PO_4, buffered with 1 mM EGTA.

Supplemental References

Erecinska M, Dagani F, Nelson D, Deas J, Silver IA (1991) Relations between intracellular ions and energy metabolism: a study with monensin in synaptosomes, neurons, and C6 glioma cells. J Neurosci 11:2410-2421.

Freundt EC, Czapiga M, Lenardo MJ (2007) Photoconversion of Lysotracker Red to a green fluorescent molecule. CELL RESEARCH-ENGLISH EDITION- 17:956.

Kasai H, Tanaka JI, Horiike Y, Matsuzaki M, Miyazaki T, Ellis-Davies GCR (2008) Protein synthesis and neurotrophin-dependent structural plasticity of single dendritic spines. Science 319:1683-1687.

Levin JI, Chen J, Du M, Hogan M, Kincaid S, Nelson FC, Venkatesan AM, Wehr T, Zask A, DiJoseph J, Killar LM, Skala S, Sung A, Sharr M, Roth C, Jin G, Cowling R, Mohler KM, Black RA, March CJ, Skotnicki JS (2001) The discovery of anthranilic acid-Based MMP inhibitors. Part 2: SAR of the 5-position and P11 groups. Bioorganic & Medicinal Chemistry Letters 11:2189-2192.

Matsuzaki M, Honkura N, Ellis-Davies GC, Kasai H (2004) Structural basis of long-term potentiation in single dendritic spines. Nature 429:761-766.

Miller MA, Barkal L, Jeng K, Herrlich A, Moss M, Griffith LG, Lauffenburger DA (2011) Proteolytic Activity Matrix Analysis (PrAMA) for simultaneous determination of multiple protease activities. Integrative biology : quantitative biosciences from nano to macro 3:422-438.
